# Oxidized Resveratrol Metabolites as Potent Antioxidants and Xanthine Oxidase Inhibitors

**DOI:** 10.3390/antiox11091832

**Published:** 2022-09-17

**Authors:** Orinamhe G. Agbadua, Norbert Kúsz, Róbert Berkecz, Tamás Gáti, Gábor Tóth, Attila Hunyadi

**Affiliations:** 1 Institute of Pharmacognosy, University of Szeged, H-6720 Szeged, Hungary; 2 Institute of Pharmaceutical Analysis, University of Szeged, H-6720 Szeged, Hungary; 3 Servier Research Institute of Medicinal Chemistry (SRIMC), H-1031 Budapest, Hungary; 4 NMR Group, Department of Inorganic and Analytical Chemistry, Budapest University of Technology and Economics, H-1111 Budapest, Hungary; 5 Interdisciplinary Centre of Natural Products, University of Szeged, H-6720 Szeged, Hungary

**Keywords:** resveratrol, antioxidant metabolism, scavengome, biomimetic oxidation, bioactivity-guided isolation, NMR spectroscopy, xanthine oxidase

## Abstract

Resveratrol is a well-known natural polyphenol with a plethora of pharmacological activities. As a potent antioxidant, resveratrol is highly oxidizable and readily reacts with reactive oxygen species (ROS). Such a reaction not only leads to a decrease in ROS levels in a biological environment but may also generate a wide range of metabolites with altered bioactivities. Inspired by this notion, in the current study, our aim was to take a diversity-oriented chemical approach to study the chemical space of oxidized resveratrol metabolites. Chemical oxidation of resveratrol and a bioactivity-guided isolation strategy using xanthine oxidase (XO) and radical scavenging activities led to the isolation of a diverse group of compounds, including a chlorine-substituted compound (**2**), two iodine-substituted compounds (**3** and **4**), two viniferins (**5** and **6**), an ethoxy-substituted compound (**7**), and two ethoxy-substitute,0d dimers (**8** and **9**). Compounds **4**, **7**, **8**, and **9** are reported here for the first time. All compounds without ethoxy substitution exerted stronger XO inhibition than their parent compound, resveratrol. By enzyme kinetic and in silico docking studies, compounds **2** and **4** were identified as potent competitive inhibitors of the enzyme, while compound **3** and the viniferins acted as mixed-type inhibitors. Further, compounds **2** and **9** had better DPPH scavenging activity and oxygen radical absorbing capacity than resveratrol. Our results suggest that the antioxidant activity of resveratrol is modulated by the effect of a cascade of chemically stable oxidized metabolites, several of which have significantly altered target specificity as compared to their parent compound.

## 1. Introduction

Resveratrol (*E*-3,5,4′-trihydroxystilbene; molecular formula: C_14_H_12_O_3_) is found naturally in many common foods including a variety of berries, tomato skin, peanuts, pistachios, and cocoa [1]. It is probably the most popular dietary polyphenol, with a myriad of reported pharmacological activities including anticancer, antioxidant, anti-inflammatory, cardio-protective, and neuroprotective effects [2,3]. The complex pharmacology of resveratrol involves its ability to interact with many important enzymes and receptor signaling pathways, as well as its ability to prevent damage connected to oxidative stress. Resveratrol is known to scavenge a variety of free radicals [4], i.e., reactive oxygen and nitrogen species (ROS and RNS, respectively, collectively referred to as RONS). Such reactive species are normal byproducts of the aerobic metabolism, and, in physiological conditions, their levels are under tight enzymatic control. The imbalance between the production and detoxification of RONS may, however, lead to the production of toxic free radicals that are at the hallmark of a variety of pathological conditions [5]. Xanthine oxidase (XO), a molybdenum-containing metalloenzyme, is an enzyme that serves as an important biological source of ROS. XO catalyzes the oxidation of hypoxanthine to xanthine and, finally, to uric acid in the presence of molecular oxygen that acts as an electron acceptor, producing superoxide anion radical (O_2_^•−^) and hydrogen peroxide (H_2_O_2_) [6]. High XO activity plays a major role in the onset of oxidative stress; therefore, this enzyme is a relevant target for not only gout treatment, but also to the prevention and/or perhaps treatment of a wide range of pathological conditions [7]. Resveratrol has been reported to inhibit the XO enzyme by decreasing uric acid formation and superoxide radical production [8,9].

Despite its high oral absorption rate (at least 70%) and much valued bioactivities, resveratrol has very poor systemic bioavailability (≈0.5%) due to extensive phase I and II biotransformation in the enterocytes and liver, and to a minor extent by gut microbiota [10]. These transformations have made it difficult to identify the metabolites responsible for the observed bioactivities and, thus, resveratrol metabolism and the bioactivity of the resulting metabolites has attracted great interest in recent years [11]. While there have been numerous reports on the bioactivities of glucuronidated and sulfated conjugates of resveratrol [12], limited knowledge is available on the metabolites formed via peroxidation and oxidation. Considering the free radical scavenging properties of resveratrol, however, the biological relevance of such oxidized metabolites may have been underestimated. It is an intriguing notion that the structure and function of polyphenolic antioxidants change upon RONS scavenging, and, depending on the antioxidant’s chemical properties and type of ROS/RNS scavenged, a complex mixture of chemically stable oxidized metabolites may be formed from such an interaction. In some cases, such metabolites may have a dramatically altered bioactivity pattern when compared to the parent antioxidant [11]. Concerning resveratrol, a study by Shingai et al. reported that biomimetic, Fe-catalyzed oxidation led to a mixture containing a variety of minor products, and, despite the rather low conversion, this mixture showed potent lipoxygenase (LOX) inhibitory activity in contrast with the inactive resveratrol itself. Several active metabolites were also obtained that, however, did not fully explain the very high increase in bioactivity [13].

These are well in line with the ‘scavengome’ concept that considers any small molecule antioxidants as pro-drug oxidizable by ROS/RNS to a broad chemical space of bioactive metabolites in a biological environment under oxidative stress [11,14]. To expand related knowledge on resveratrol, in the current study, it was our objective to use a chemical-oxidation-driven, diversity-oriented synthetic strategy to obtain its new bioactive derivatives. To simultaneously aim at obtaining biologically relevant resveratrol metabolites and their semi-synthetic analogs, we decided to involve both biomimetic and non-biomimetic approaches. To explore the biomedical potential of these oxidized resveratrol metabolites/analogues, XO was selected as the first target of our interest.

## 2. Materials and Methods

### 2.1. General Information

Resveratrol (**1**) with a purity of >98% was purchased from Career Henan Chemical Co., Ltd. (Zhengzhou, China). HPLC solvents were purchased from ChemLab (Zedelgem, Belgium), and organic solvents and reagents (bis(trifluoroacetoxy)iodo)benze (PIFA), (diacetoxyiodo)benzene (PIDA), 2,2′-azobis(2-amidinopropane) dihydrochloride (AAPH), oxone, anhydrous, monobasic, potassium phosphate (P5379), and xanthine (0626) were purchased from Sigma Aldrich (Budapest, Hungary). Periodic acid, iron (III) chloride hexahydrate and sodium periodate were purchased from Reanal Laboratory Chemicals (Budapest, Hungary).

### 2.2. General Procedures for Resveratrol Oxidation

Several oxidative reactions were carried out on resveratrol, as shown in Table 1. The reactions were monitored by thin-layer chromatography, solid phase: Silica 60 F254 (250 µm, Merck Co., Ltd., Darmstadt, Germany); liquid phase: chloroform–ethyl acetate–formic acid (2.5:1:0.1, *v/v*/*v*) at regular intervals with visualizations performed under UV light, λ_1_ = 254 nm, and λ_2_ = 365 nm. At the end of each reaction, the mixtures were evaporated, extracted with ethyl acetate, and evaporated in vacuo. As a pre-purification step, each residue was filtrated through silica with hexane–acetone (1:1, *v/v*), and thereafter evaporated in vacuo. The mixtures were dissolved in CH_3_CN, and a 10 µL aliquot of each mixture was analyzed by HPLC (PU-2080 pumps; AS-2055 Plus autosampler; MD-2010 Plus PDA detector, Jasco Co., Ltd., Tokyo, Japan) under the following conditions: column, Kinetex XB-C18 (250 × 4.6 mm, 5 µm); solvent system, water (solvent A) and CH_3_CN (solvent B): elution, linear gradient from 25% solvent B to 75% solvent B for 25 min, and then isocratic mode for 75% solvent B for 2 min; flow rate, 1 mL/min; detection, 199–650 nm. The purity of the isolated metabolites was also determined using the same analytical HPLC condition.

Isolation and purification of metabolites from mixtures was conducted using an Armen Spot Prep II integrated HPLC purification system (Gilson, Middleton, WI, USA) using a Kinetex XB-C18 or Biphenyl column (250 × 21.2 mm, 5 µm) and appropriately chosen eluents.

### 2.3. Reaction with PIFA in Acetonitrile (Ox1)

Resveratrol (300 mg) was dissolved in acetonitrile (150 mL), an acetonitrile solution of PIFA (565.14 mg/150 mL) was added, and the mixture was stirred for 5 h at room temperature. The solvent was evaporated using a rotary evaporator, and the residue was partitioned between water (150 mL) and ethyl acetate (3 × 100 mL). Dry residue of the combined organic layers was purified by silica gel column chromatography eluted with acetone/hexane (1:1, *v/v*), and thereafter evaporated in vacuo. The residue was subsequently purified by preparative HLPC using an isocratic elution of CH_3_CN-H_2_O (28:72, *v/v*) on a biphenyl column to obtain compound **6** (39.80 mg). Compound **6** was further purified by HPLC on a Luna Silica column (250 × 4.6 mm, 5 µm, 100Å) using an elution of cyclohexane–isopropanol (86:14, *v/v*) to obtain 10.13 mg of the pure compound.

### 2.4. Reaction of Resveratrol with AAPH and NaIO_4_ (Ox2)

To a solution of resveratrol (500 mg) in acetonitrile (250 mL), an aqueous solution of AAPH (890 mg/250 mL) and an acetonitrile solution of NaIO_4_ (468.75 mg/250 mL) were added, and the mixture was stirred for 23 h at 65 °C. The reaction was stopped by adding an aqueous solution of reduced glutathione (541.12 mg/100 mL), keeping it in the same conditions for 5 more min as before. Then, it was cooled down in an ice bath. The solvent was evaporated on a rotary evaporator, and the residue was partitioned between water (250 mL) and ethyl acetate (4 × 200 mL) and evaporated to provide a combined dry residue (753.96 mg). The residue was purified by preparative HLPC on a C18 column with isocratic elution of CH_3_OH-H_2_O (50:50, *v/v*) to provide a fraction containing **3** (46.57 mg) and **5** (24.00 mg). The purified fractions were further separated using CH_3_OH-H_2_O (45:55, *v/v*) on the same column to obtain compounds **3** (12.93 mg) and **5** (11.38 mg).

### 2.5. Reaction of Resveratrol with PIDA in Acetonitrile (Ox3)

Resveratrol (100 mg) was dissolved in acetonitrile (25 mL), an acetonitrile solution of PIDA (282.20 mg/75 mL) was added, and the mixture was stirred for 2 h at room temperature. The reaction was stopped by adding an aqueous solution of reduced glutathione (269.25 mg/30 mL). The solvent was evaporated on a rotary evaporator, and the residue was partitioned between water (100 mL) and ethyl acetate (3 × 100 mL). Dry residue of the combined organic layers was purified by preparative HLPC using the biphenyl column and solvent, CH_3_OH-H_2_O (52:48, *v/v*), resulting in compound **5** (6.37 mg).

### 2.6. Reaction with PIFA in Ethanol (Ox4)

To a solution of resveratrol (250 mg) in ethanol (50 mL), an ethanol solution of PIFA (471 mg/200 mL) was added, and the mixture was stirred for 90 min at room temperature. The reaction was stopped with an aqueous solution of reduced glutathione (673.75 mg/187.5 mL). The solvent was evaporated on a rotary evaporator, and the residue was partitioned between water (250 mL) and ethyl acetate (3 × 300 mL). The dry residue of the combined organic layers was purified by preparative HLPC on a biphenyl column with an isocratic elution of CH_3_CN-H_2_O (31:69, *v*/*v*) to produce compounds **7** (24.80 mg), **8** (33.98 mg), and **9** (13.30 mg). Further purification was carried out on the compounds on the same column but using an elution of CH_3_OH-H_2_O (52:48, *v*/*v*) to obtain compounds **7** (11.93 mg) and **8** (22.62 mg). Compound **9** was further purified by HPLC on a Luna Silica column (250 × 4.6 mm, 5 µm, 100 Å) using an elution of cyclohexane-isopropanol (85:15, *v*/*v*) to obtain 8.38 mg of pure compound.

### 2.7. Reaction of Resveratrol with Periodic Acid and Oxone in Ethanol (Ox5)

Resveratrol (600 mg) was dissolved in ethanol (300 mL), an ethanol solution of oxone (4.05 mg/150 mL) was added, and the mixture was stirred for 5 min at room temperature. An ethanol solution of periodic acid (396 mg/180 mL) was subsequently added, and the mixture was stirred for further 7 h under the same conditions. The reaction was stopped by adding an aqueous solution of reduced glutathione (1615.50 mg/150 mL). The solvent was evaporated on a rotary evaporator, and the residue was partitioned between water (250 mL) and ethyl acetate (3 × 200 mL). Dry residue of the combined organic layers was purified by preparative HLPC using a biphenyl column with an isocratic elution using CH_3_OH-H_2_O (54:46, *v/v*) to obtain compounds **3** (167.30 mg), **4** (61.94 mg), and a mixture containing **7** (60.21 mg). Further purification was carried out on the fractions under the same conditions as above to obtain **3** (167.30 mg), **4** (18.30 mg), and **7** (18.66 mg) in pure form.

### 2.8. Reaction of Resveratrol with FeCl_3_ and H_5_IO_6_ in Acetonitrile (Ox6)

Resveratrol (480 mg) was dissolved in acetonitrile (240 mL), an acetonitrile solution of iron chloride hexahydrate (17.04 mg/80 mL) was added, and the mixture was stirred for 5 min at room temperature. An acetonitrile solution of periodic acid (384 mg/450 mL) was subsequently added, and the mixture was stirred for a further 17 h. The reaction was stopped by adding an aqueous solution of reduced glutathione (1293 mg/240 mL). The solvent was evaporated on a rotary evaporator, and the residue was partitioned between water (250 mL) and ethyl acetate (3 × 300 mL). Dry residue of the combined organic layers was purified by preparative HLPC using a biphenyl column and an isocratic elution of CH_3_OH-H_2_O (51:49, *v/v*) to obtain compounds **2** (29.94 mg), **3** (27.06 mg), and **4** (63.61 mg). Further purification was carried out on the C18 column with isocratic elution CH_3_OH-H_2_O (42:58, *v/v*) to obtain compounds **2** (12.01 mg), **3** (9.13 mg), and **4** (22.27 mg).

### 2.9. Reaction of Resveratrol with FeCl_3_ and H_5_IO_6_ in Ethanol (Ox7)

Resveratrol (360 mg) was dissolved in ethanol (180 mL), an ethanol solution of iron chloride hexahydrate (12.78 mg/60 mL) was added, and the mixture was stirred for 5 min at room temperature. An ethanol solution of periodic acid (396 mg/180 mL) was subsequently added, and the mixture was stirred for a further 17 h. The reaction was stopped by adding an aqueous solution of reduced glutathione (969.9 mg/180 mL). The solvent was evaporated on a rotary evaporator, and the residue was partitioned between water (250 mL) and ethyl acetate (2 × 250 mL). Dry residue of the combined organic layers was purified by preparative HLPC using a biphenyl column and an isocratic elution of CH_3_OH-H_2_O (51:49, *v/v*) to obtain a fraction mixture containing compound **3** (107.99 mg). This fraction was further purified by preparative HPLC using a C18 with an isocratic elution of CH_3_OH-H_2_O (42:58, *v/v*) and a semi-preparative HPLC Gemini-NX C18 column (250 × 10.0 mm, 5 µm) with CH_3_OH-H_2_O (42:58, *v/v*) as the eluent to obtain **3** (3.11 mg) in pure form.

### 2.10. Structure Elucidation

Structure elucidation of the isolated compounds was based on their molecular formulas obtained by high-resolution mass spectrometry (HRMS) and on detailed nuclear magnetic resonance (NMR) studies. HRMS spectra were acquired on an FTHRMS-Orbitrap (Thermo-Finnigan) mass spectrometer equipped with an ESI ion source typically used in positive ionization mode, except for compound **2** for which both positive- and negative-mode spectra were taken (see the Supplementary Material, Figures S8–S15). ^1^H NMR, ^13^C, APT, HSQC, HMBC, ^1^H,^1^H-COSY, NOESY, and one-dimensional selective NOE spectra were recorded in acetone-*d*_6_ and DMSO-*d*_6_ on a Bruker Avance III 500 NMR equipped with a cryo-probehead and on Bruker Avance spectrometers. Chemical shifts (δ) are provided on the δ-scale and referenced to the solvents (acetone-*d*_6_: δH = 2.05 and δC = 29.9 ppm; DMSO-*d*_6_: δH = 2.50 and δC = 39.5 ppm); coupling constant (*J*) values are expressed in Hz. The pulse programs were taken from the Bruker software library (TopSpin 3.5). Full ^1^H and ^13^C signal assignment was performed by means of comprehensive one- and two-dimensional NMR methods using widely accepted strategies [15,16].

^1^H assignments were accomplished using general knowledge of chemical shift dispersion with the aid of the ^1^H-^1^H coupling pattern. To facilitate the understanding of NMR signal assignments in the Supplementary Material, the stereo structures with the δ^1^H and δ^13^C and *J* values are also depicted on the NMR spectra (Figures S16–S56).

### 2.11. Bioactivity Studies

#### 2.11.1. Xanthine Oxidase Inhibitory Activity

XO inhibitory activity was tested using the protocol described previously by Noro et al. with slight modifications [17]. Briefly, reagents were 50 mM phosphate buffer (pH 7.5), 0.15 mM xanthine solution dissolved in the phosphate buffer (pH 7.5), and 0.1 U/mL XO enzyme-buffer solution. Compound solutions were prepared by dissolving in DMSO and subsequently buffer. In a 96-well UV plate, 100 µL xanthine solution was added to 150 µL solution of compounds, before adding the enzyme solution (50 µL) by pump to initiate the formation of uric acid. The increase in uric acid was determined at 290 nm for approximately 150 s at 37 °C, with allopurinol as the reference using a FluoStar Optima plate reader (BMG Labtech, Ortenberg, Germany). The XO activity of a blank and allopurinol used as negative and positive controls, respectively, was also determined. The slope (m) for the compounds and blank and positive controls was obtained, and the XO inhibitory activity was expressed as the percentage inhibition of XO in the above-mentioned assay mixture system, calculated as follows:

% Inhibition = 100 − (m_compounds_/m_blank_ × 100)

Dose–effect studies on the most bioactive compounds (1.5625–200 µM) and resveratrol (25–400 µM) were used to determine the concentration that inhibits 50% of the XO enzyme activity. Enzyme kinetic studies of the bioactive compounds was also performed. The sigmoidal dose–response model, dose–response inhibition curve models, and Lineweaver–Burk transform plots were obtained by using the software GraphPad Prism 8.0 (La Jolla, CA, USA), and these were used to determine the IC_50_ values of the compounds and to determine their mode of inhibition.

#### 2.11.2. DPPH Radical Scavenging Activity

The DPPH free radical scavenging assay was performed on the basis of the method of Fukumoto et al. [18] with some modification. One hundred microliters of 0.1 mM solution of DPPH reagent prepared in methanol was added to 100 µL solution of compounds at concentrations ranging from 2 to 500 µM. Absorbance of each well including the blank (containing 100 µL methanol) and standard (ascorbic acid) was measured at 550 nm for 30 min using a FluoStar Optima plate reader (BMG Labtech, Ortenberg, Germany). A sigmoidal dose–response model of the blank corrected values was used to calculate IC_50_ values of compounds using GraphPad Prism 8.0 (La Jolla, CA, USA).

#### 2.11.3. Oxygen Radical Absorbance Capacity (ORAC) Assay

The oxygen radical absorbance capacity was determined as described by Dávalos et al. [19] with slight modifications. Briefly, the reaction was carried out in 75 mM sodium phosphate buffer (pH 7.4), and the final volume was 200 µL in each well in a 96-well polystyrene microplate with black sides and flat clear bottom. Each well contained 20 µL of sample at 100 µM concentration (compound dissolved in DMSO), 70 nM fluorescein, and 12 mM AAPH solution. A blank containing sodium phosphate buffer, fluorescein, and AAPH, as well as a range of calibration solutions using Trolox ^TM^ (from 3.125 to 100 µM) as the standard antioxidant, were also used in each assay. All reaction mixtures were prepared in triplicate. The fluorescence was read with an excitation wavelength of 485 nm and an emission filter of 520 nm every 90 s cycle time for 120 cycles using a FluoStar Optima plate reader (BMG Labtech, Ortenberg, Germany). The antioxidant abilities, expressed as Trolox equivalents, were obtained by calculating the area under the fluorescence decay curve (AUC) and interpolating the net AUC against the Trolox standard curve performed using GraphPad Prism (GraphPad version 8.0 Software, La Jolla, CA, USA).

## 3. Results and Discussion

### 3.1. Preparation and Evaluation of Oxidized Resveratrol Metabolites

Resveratrol was transformed by a variety of oxidative reagents. These included hypervalent iodine (III) reagents PIFA/PIDA and AAPH that were found in our previous studies to reasonably mimic reactions that could occur via ROS scavenging by an antioxidant [20,21]. To further increase the explorable chemical complexity, periodic acid was also selected with oxone or iron (III) chloride as a co-oxidant. As a biomimetic element in the work-up procedure, most reactions were terminated by adding reduced glutathione (GSH), an abundant intracellular antioxidant [22]. The reaction mixtures (Ox1–Ox7) were then analyzed by HPLC-PDA for their chromatographic fingerprints (Supplementary Material, Figures S1–S7) and subjected to XO inhibitory assay that also served as a guidance for isolation of major bioactive metabolites. Results, along with a listing of compounds isolated from each mixture, are compiled in Table 1. 

### 3.2. Structure Elucidation of the Isolated Compounds

The ^1^H NMR spectrum of our starting material **1** *E*-resveratrol (3,5,4′-trihydroxystilbene, C_14_H_12_O_3_) consists of one set of three aromatic hydrogens coupled in an AX_2_ system (δ 6.54 d, 2H, ^4^*J* = 2 Hz and δ 6.28 t, 1H, 2 Hz), another set of four aromatic hydrogens coupled in an AA′XX′ system (δ 7.24 dm, 2H, ^3^*J* = 8.4 Hz and δ 6.79 dm, 2H, 8.4 Hz), and two doublet signals (δ 7.03 d, 1H, ^3^*J* = 16 Hz and δ 6.89 d, 1H, 16 Hz) indicating the presence of a HC = CH double bond with *E* configuration [23].

For compound **2**, HRMS revealed an elemental composition of C_14_H_11_O_3_Cl (Supplementary Material, Figures S8 and S9) indicating that one hydrogen is substituted with a Cl. The ^1^H NMR spectrum of **2** is rather similar to that of **1**, with only one characteristic change, i.e., the original AX_2_ system of the 3,5-dihydroxyphenyl moiety turned into an AX system (δH-6 6.79 d, 1H, ^4^*J* = 2 Hz and δH-4 6.48 d, 1H, 2 Hz), indicating that compound **2** is 2-chloro-*E*-resveratrol. This corroborates with previous studies by Li et al. [24]. To achieve complete ^1^H and ^13^C NMR assignment, ^1^H, ^13^C-APT, HSQC, HMBC, COSY, and NOESY measurements were utilized (Figures S16–S19). 

The molecular formula of both compounds **3** and **4** was established as C_14_H_11_O_3_I (Figures S10 and S11). In the ^1^H NMR spectrum of **3** (Figure S20), the signals of 3,5-dihydroxyphenyl moiety appeared at δ6.70 as a singlet with 2H intensity corresponding to the isochronic H-2,6 atoms, revealing the iodination at C-4. It is remarkable that in the ^13^C spectrum, the effect of an iodine substituent is accompanied by a characteristic diamagnetic shift, as shown by the δC-4 signal at 73.8 ppm (Figure S21). Compound **3** was previously reported by Lee et al. [25,26] as products formed from the halogenation of resveratrol. 

Compound **4** had a similar aromatic substitution pattern as that of **2**, i.e., an AX system (δH-6 6.78 d, 1H, ^4^*J* = 2 Hz and δH-4 6.48 d, 1H, 2 Hz). In the 2-iodinated-*E*-resveratrol product, the δC-2 signal appeared at 79.1 ppm. The utilized NMR spectra are compiled in Figures S24–S27.

**Figure 1:**
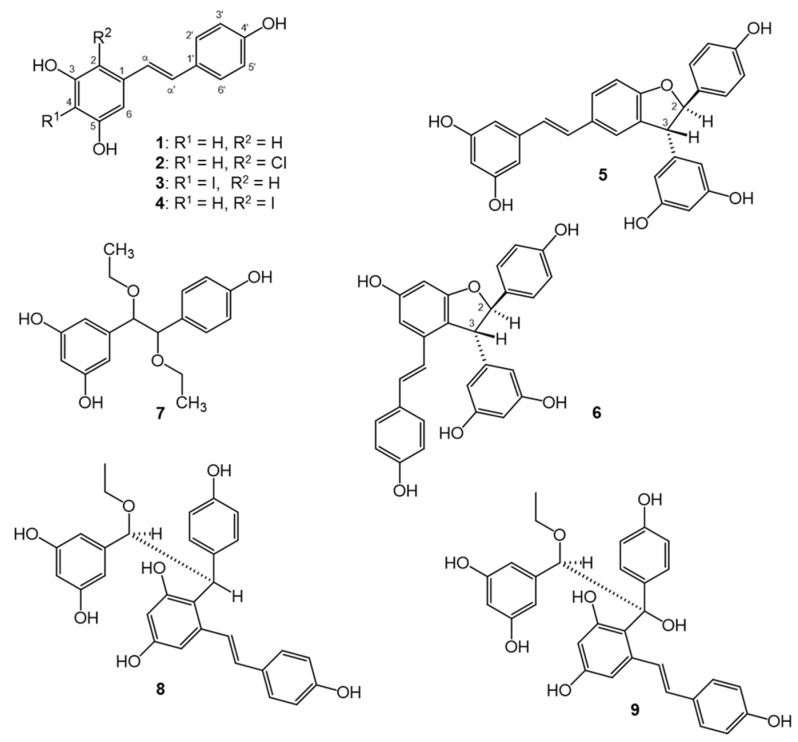
Structures of resveratrol (**1**) and its metabolites obtained by chemical oxidation (**2**–**9**). Each optically active compound (**5**, **6**, **8**, **9**) is racemate; for simplicity, only one enantiomer is presented. For compounds **7** and **9**, the relative configuration could not be determined.

In the case of both compounds **5** and **6**, HRMS indicated the molecular formula of C_28_H_22_O_6_ (Figures S12 and S13), and their ^1^H NMR spectra were in good correspondence with those previously reported for δ-viniferin (**5**) and ε-viniferin (**6**) [27,28,29]. However, the use of non-exact names in the literature may lead to confusions. In our study, both 2,3-dihyro-benzofuran derivatives were racemic mixtures. Considering the relative positions of the 2,3-substituents, one should differentiate between *cis* and *trans* diastereomers. For the sake of simplicity, only the enantiomeric forms with 2*R* configuration are depicted in Figure 1. Compound **5**, i.e., (*E*)-(±)-2,3-*trans*-δ-viniferin, is structurally (±)-(*E*)-5-(3,5-dihydroxystyryl)-3-(3,5-dihydroxyphenyl)-2-(4-hydroxyphenyl)-*trans*-dihydrobenzofuran, whereas compound **6**, (*E*)-(±)-2,3-*trans*-ε-viniferin, corresponds to the (±)-6-hydroxy-(*E*)-4-(4-hydroxystyryl)-3-(3,5-dihydroxyphenyl)-2-(4-hydroxyphenyl)-*trans*-dihydrobenzofuran structure. A recent study on a series of 2,3-disubstituted dihydrobenzofuran derivatives demonstrated the usefulness of the *J*(H-2,H-3) coupling constant to distinguish between *cis*/*trans* isomers [30]. Even though both compounds **5** and **6** are *trans*-substituted at the 2,3 positions of the nearly planar dihydrobenzofuran ring, the peri-effect, caused by the 4 substituent of compound **6**, alters the geometry of the dominant conformer as compared to that of compound **5**. This manifests in differences in the *J*(H-2,H-3) coupling constants, i.e., 8 Hz and 5 Hz for compounds **5** and **6**, respectively. The NMR spectra utilized for the unambiguous structure elucidation of **5** and **6** are compiled in Figures S28–S37.

On the basis of the HRMS data, an elemental composition of C_16_H_22_O_5_ (Figure S14) was established for compound **7,** indicating the incorporation of two ethoxy groups into the structure of resveratrol. In the ^1^H and ^13^C NMR spectra of **7** (Figures S38 and S39), signals of the 3,5-dihydroxyphenyl and 4-hydroxyphenyl moieties remained well identifiable, but instead of the H–C=C–H double bond, the signals of a diethoxy substituted chiral H–C–C–H group appeared (δH = 4.14 d/δC = 85.9 and δH = 4.18 d/δC = 85.6, *J*(H,H) = 6.6 Hz). Their differentiation was supported by the NOESY and HMBC measurements (Figures S40 and S41). In the uniform NMR spectra, nothing indicated the presence of a diastereomeric mixture, but neither the *J*(H,H) coupling nor the results of the NOESY experiment allowed for the identification of *threo* or *erithro* configuration.

The molecular formula of **8** was established as C_30_H_28_O_7_ by means of HRMS ([M + H]^+^, calculated: 501.19078, found 501.19189), suggesting an ethoxy group attached to a dimer. The ^1^H NMR spectrum of **8** (Figure S43) identified the presence of four groups of aromatic hydrogens, namely, two sets of four aromatic hydrogens coupled in an AA′XX′ system (δ 7.24 dm, 2H, ^3^*J* = 8.6 Hz, δ 6.79 d, 2H, and δ 7.10 dm, 2H, ^3^*J* = 8.6 Hz, δ 6.71 dm, 2H), corresponding to 4-hydroxyphenyl moieties, and, in addition, one set of three aromatic hydrogens coupled in an AX_2_ system at δ 6.36 d, 2H, ^4^*J* = 2 Hz, δ 6.27 t, 1H (3,5-dihydroxyphenyl group) and at the and an AX system (δ 6.40 d, 1H, ^4^*J* = 2.5 Hz, δ 6.23 d, 1H). Appearance of the two doublet signals (δ 6.58 d, 1H, ^3^*J* = 16 Hz, δ 6.89 d, 1H) proved the presence of a HC = CH double bond with *E* configuration. The signals at *δ* 3.65 q, 2H, *J =* 6.8 Hz, *δ* 1.23 t, 3H are unique to the ethoxy group, whereas the δH 5.36 d, 1H, ^3^*J* = 3 Hz and δ4.62 br, 1H signals correspond to an ethoxy and 2-substituted *E*-resveratrol substituted chiral H–C–C–H group.

The partial broadening of several ^1^H signals refers to hindered rotation. ^1^H, ^13^C-APT, HSQC, HMBC, ^1^H,^1^H-COSY, and NOESY spectra were utilized for signal assignment (Figures S43–S48). In exploring the order of connection of different structural units of compound **8**, the characteristic long-range HMBC correlations, marked with black arrows (Figure S32), were extremely effective. Considering the uniform NMR spectra, nothing indicated the presence of a diastereomeric mixture for the racemic compound **8**.

To facilitate an understanding of the relative configurations of the two stereogenic centers, the *S* configuration was arbitrarily set for the O–CH- center. Examining the three staggered conformers along the H-C → C-H,O Et carbon bond, the ^3^*J* (H,H) = 3 Hz coupling suggested that the share of the *anti*-conformer in the equilibrium is negligible. Thorough evaluation of steric proximities detected in the NOESY spectrum (red double arrows in Figure S48) identified the only conformer that is consistent with NOESY results, i.e., *S*,*S* configuration of the depicted enantiomer of compound **8**.

In the case of compound **9**, HRMS measurements did not provide an identifiable m/z value for the molecular ion or any obvious fragments, neither in positive nor in negative mode. Nevertheless, the ^1^H-NMR, ^13^C-APT, HSQC, HMBC, and NOESY spectra (Figures S49–S53) taken in acetone-*d*_6_ clearly revealed that compound **9** is also an ethoxy-substituted dimer with a structure rather similar to that of **8**. The one characteristic difference between these compounds is that **9** contains a quaternary C–OH instead of the sp^3^ CH of **8**. Due to the increased steric crowding, the rotation along the H-C → C-OH carbon bond is hindered, resulting a partial broadening of several ^1^H and ^13^C signals (δ CH 5.24 s/81.5 and δ HO-C 4.53 br ppm, and even the δ C remained under the noise level). Due to the broad CH and OH signals, the NOESY measurement did not allow for the detection of all the sterically close hydrogen atoms, and thus the identification of the relative configuration of the two stereogenic centers was not possible. To overcome the hindered rotation, we raised the temperature to 80 °C in DMSO-*d*_6_ (Figures S54–S56), which made the signals sharper in the ^1^H-NMR, ^13^C-APT, and HSQC spectra. However, the CH and C-OH signals (5.16 and 4.54 ppm, respectively) remained relatively broad. The selective one-dimensional ROE experiment on these hydrogens revealed several characteristic steric proximities (Figure S56, see red arrows), but these did not allow a definite distinction between the possible relative configurations.

The structures of compounds **1**–**9** are shown in Figure 1.

The compounds obtained represent a structural diversity that was expectable from our chemical approach. Concerning their biological relevance, it may be worth stressing that compounds **2**, **5**, and **6** are also expectable products of a resveratrol molecule scavenging free radicals in a biological environment. For example, chlorine substitution (as in compound **2**) may be the result of a reaction of a resveratrol radical with chloride ions that are abundant in intra- and extracellular liquids. Further, resveratrol molecules have strong self-association through π–π stacking in aqueous medium [31,32]. This produces radical coupling reactions, leading to dimers such as **5** and **6**, possible in a biological environment under oxidative stress, regardless of the very low concentrations achievable in vivo. Compounds **3** and **4** are valuable to expand chemical space towards halogen-substituted derivatives related to the biologically more relevant compound **2**. In addition to this, the ethoxy substituted compounds **7–9** are of further interest for their potential formation when resveratrol becomes oxidized in the presence of ethanol, e.g., during the aging of red wine, which is connected to a gradual loss of resveratrol content [33]. Compounds **8** and **9** may also hold further pharmacological potential, considering that some related stilbene dimers from *Dracaena cochinchinensis* were previously reported as anti-Helicobacter pylori and thrombin inhibitory [34] agents, and as ErbB1/ErbB2 kinase inhibitors [35].

### 3.3. Bioactivity of the Isolated Compounds

#### 3.3.1. Xanthine Oxidase Inhibitory Activity

XO inhibitory activity of the compounds was estimated on the basis of their ability to prevent uric acid formation from xanthine. Biomimetic oxidation of resveratrol resulted in the formation of some metabolites with marked increase in the ability to inhibit this enzyme with regards to resveratrol, as shown in Table 2.

Unlike resveratrol, compounds **2**, **3**, and **4** exerted a nearly complete inhibition of XO at 100 µM. Subsequent determination of the dose–response curves revealed that compounds **2** and **4** showed similar efficacy in XO inhibition as the reference drug allopurinol. To evaluate the mode of inhibition of the isolated active compounds, enzyme inhibition kinetics studies were performed. Our results revealed that compounds **2** and **4** inhibit XO competitively, i.e., they bind to the active center of the enzyme. Compound **3** and the viniferins (**5** and **6**) were observed to be mixed-type inhibitors, and they are reported here for the first time as potent inhibitors of XO. Lineweaver–Burk transform plots of compounds **2**–**6** are presented in the Supplementary Material, Figures S57–S61.

**Figure 2:**
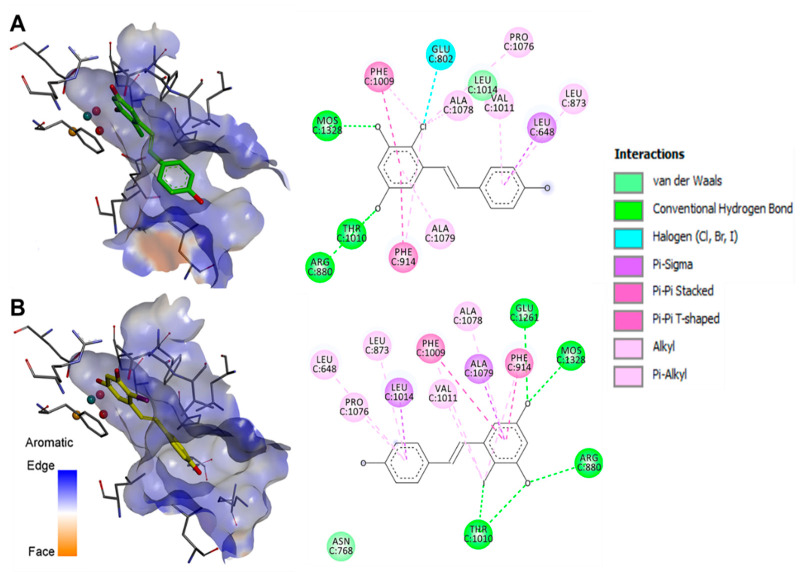
Best-docked poses of compounds **2** (**A**) and **4** (**B**). Three-dimensional orientation and aromatic edge/face receptor surface is shown for both compounds at identical viewing angle and zoom (**left**), along with the 2D interpretation of the ligand–residue interactions with the 3NVY protein (**right**).

Subsequently to the enzyme kinetic studies, in silico docking was performed with the most potent competitive inhibitors **2** and **4**. To this, we followed our previously published approach using the 3NVY protein, and the docking site was defined in a 10 Å radius around the experimental position of quercetin bound to the active site of XO [36]. Results for the best-docked poses are presented in Figure 2.

In the best docked orientation of **2** and **4** in the active site, several hydrogen bonds were observed between the phenolic ring containing the two meta-hydroxy groups and several amino acid residues. In both compounds, a H-bond was observed with the molybdopterin residue, Mos1328. The halogens found in **2** and **4** formed a H-bond with Glu802 and Thr1010, respectively. The orientation of **2** and **4** in the active site of XO was further stabilized by π–π interactions between the compounds’ A-ring and the aromatic ring of residues Phe914 and Phe1009. Interaction with these amino acids constrained the compounds to a well-defined plane perpendicular to the base plane of the molybdenum center in the active site.

Proton transfer from the molybdopterin residue’s hydroxyl group to Glu1261 is an initial step required for the conversion of xanthine to uric acid by XO. Glu802, Arg880, and Thr1010 have also been reported to be essential in the catalytic transformation of xanthine to uric acid [37,38]. Therefore, interactions observed between **2** and **4** and these residues provide a reasonable mechanistic background to the compounds’ XO inhibitory activity. The orientation and interactions of the hydroxy groups of **2** and **4** are similar to the 5-OH and 7-OH of quercetin and apigenin, both of which are well-studied as XO inhibitors [36]. Planarity, extended conjugation and, in the case of flavones, the presence of a 7-OH group were reported as the key features for small-molecule XO inhibitors [36,39,40]. These rules apply for several stilbenes and flavonoids, and XO inhibition is an activity that fits well to their widely acknowledged antioxidant effect in vivo [41].

#### 3.3.2. Free Radical Scavenging Activity

To assess the compounds’ potential direct antioxidant activity in comparison with that of resveratrol, their DPPH scavenging activity and oxygen-radical-absorbing capacity (ORAC) was also evaluated. These two antioxidant assays are somewhat complementary to each other, since DPPH may be scavenged through either single-electron transfer (SET) or hydrogen atom transfer (HAT), while reaction mechanism in the ORAC assay is mainly HAT [42]. Results are presented in Table 3.

In this study, the DPPH free radical scavenging activity of resveratrol corroborated with some earlier reports [43,44], and, expectably, we found a potent antioxidant also in the ORAC assay, as reported previously [45].

Notably, several of the oxidized derivatives of resveratrol were found to be similarly or more potent free radical scavengers than their parent compound in either or both bioassays. Concerning structure–activity relationships, it was found that substituting one of the aromatic rings with chlorine, as in compound **2**, increased the DPPH scavenging activity, unlike iodine substitution (**3** and **4**). Iodine substitution, however, significantly increased the compounds’ capacity for HAT. Substitution of a phenol ring with electron-donating or electron-withdrawing groups markedly alters the antioxidant activity of polyphenols [46], and substitution with electron-withdrawing halogens modulates free radical scavenging activity depending on the position and the number of halogen substituents [47]. The presence of halogens was reported to confer increased potential for HAT [48], which is in agreement with our results on the iodine-substituted compounds **3** and **4**. The ethoxy-substituted dimer **8** also had higher TE value than resveratrol, and similar antioxidant abilities were reported for other resveratrol dimers without the benzofuran ring [49]. 

(*E*)-(±)-2,3-*trans*-ε-Viniferin (**6**) was also more potent than resveratrol concerning its ability to neutralize peroxyl and alkoxyl radicals produced by AAPH [50], which is likely a direct consequence of its higher number of phenolic hydroxyl groups prone to HAT reactions.

## 4. Conclusions

The chemical oxidation of resveratrol led to the formation of a chemically diverse set of new derivatives. Several of the compounds obtained are expectably among those that may be formed when resveratrol scavenges ROS/RNS in a biological environment, such as compounds **2**, **5**, and **6**, and therefore these are suggested here as possible metabolites. Three ethoxy substituted compounds were also obtained in which the solvent ethanol reacted with resveratrol’s oxidized intermediates; these compounds may have relevance as possible constituents of aged red wines.

Except for the ethoxy derivatives, all compounds showed greater XO inhibitory activities than resveratrol, and the most potent chlorine substituted compound **2** by nearly two orders of magnitude. Further, when compared to resveratrol, most compounds were similarly or more potent free scavengers of DPPH radicals and/or ROO^•^/RO^•^ radicals produced by AAPH. Accordingly, our findings suggest that free radical scavenging by resveratrol leads to a wide range of valuable metabolites whose increased chemical complexity may also manifest in an unexpected increase in their overall antioxidant activity.

On the margin of our results, however, it needs to be stressed that our approach was purely chemical in this work, and none of the identified derivatives were confirmed as metabolites in an actual biological environment. We merely utilized a somewhat philosophical consideration, i.e., the ‘scavengome’ concept, as a guiding principle towards an antioxidant-inspired and diversity-oriented expansion of chemical space, which led to a very high hit-rate on a relevant biochemical target (XO). While this is strongly encouraging to such drug discovery initiatives, the true biological relevance of the scavengome of resveratrol remains to be evaluated by future studies.

## Figures and Tables

**Table 1:** Oxidation of resveratrol, XO inhibitory activity of the oxidized mixtures, and list of compounds isolated from each. Results are expressed as mean ± SEM, *n* = 3; *: *p* < 0.05 by one-way ANOVA using Dunnett’s multiple comparison test to the parent compound resveratrol. Resveratrol was tested at 100 µM, and mixtures Ox1–Ox7 at 100 µM of resveratrol equivalents.

ID	Oxidants	Experimental Conditions ^a^	XO Inh. (%)	Compound Isolated
Resveratrol	-	-	49.9 ± 6.9	-
Ox1	PIFA	CH_3_CN, r.t. 5 h	42.5 ± 3.3	**6**
Ox2	AAPH, NaIO_4_	CH_3_CN, 65 °C, 23 h ^b^	37.6 ± 5.3	**3**, **5**
Ox3	PIDA	CH_3_CN, r.t., 2 h ^b^	32.0 ± 2.1 *	**5**
Ox4	PIFA	EtOH, 2 h ^b^	47.1 ± 1.6	**7**, **8**, **9**
Ox5	H_5_IO_6_, Oxone	EtOH, r.t, 7 h ^b^	55.8 ± 0.9	**3**, **4**
Ox6	H_5_IO_6,_ FeCl_3_	CH_3_CN, r.t. 17 h ^b^	67.8 ± 1.8 *	**2**, **3**, **4**
Ox7	H_5_IO_6_, FeCl_3_	EtOH, r.t., 17 h ^b^	75.7 ± 4.9 *	**3**

^a ^Experimental conditions are provided as solvent, temperature, and time. ^b^ Reactions were terminated by adding reduced glutathione.

**Table 2:** XO inhibition percentage of compounds **1**–**9** at 100 µM, and their IC_50_ values. Results are expressed as mean ± SEM. *: *p* < 0.05 by one-way ANOVA using Dunnett’s post hoc test as compared to resveratrol, *n* = 4; due to the large differences, statistical significance was not evaluated for the IC_50_ values.

Compounds	XO Inh (%)	XO IC_50_ (µM)
Resveratrol (**1**)	55.6 ± 1.1	119.4 ± 2.0
**2**	90.6 ± 4.4	4.8 ± 0.8
**3**	97.2 ± 4.9 *	15.3 ± 1.4
**4**	93.8 ± 1.3 *	6.4 ± 0.5
**5**	77.4 ± 2.1	16.4 ± 1.3
**6**	69.1 ± 3.1	22.8 ± 6.3
**7**	14.4 ± 3.0 *	>1000
**8**	15.1 ± 1.7	233.3 ± 5.1
**9**	31.5 ± 4.8	234.1 ± 4.8
Allopurinol	97.3 ± 0.9 *	5.9 ± 0.9

**Table 3:** ORAC values and DPPH radical scavenging activity of compounds **1**–**9**. Results are expressed as mean ± SEM. *: *p* < 0.05 by one-way ANOVA using Dunnett’s multiple comparison test to the parent compound, resveratrol. *n* = 2 for DPPH and 3 = ORAC assay.

Compounds	DPPH IC_50_ (µM)	ORAC (Trolox Equivalents, TE)
Resveratrol (**1**)	27.7 ± 1.4	8.9 ± 0.2
**2**	15.8 ± 1.0	9.9 ± 0.5
**3**	41.0 ± 1.5	11.6 ± 0.2 *
**4**	51.7 ± 1.6	12.5 ± 0.7 *
**5**	340.7 ± 2.1	6.3 ± 0.3
**6**	92.1 ± 1.6	15.2 ± 0.5 *
**7**	>500	8.1 ± 0.5
**8**	53.1 ± 1.5	13.9 ± 0.1 *
**9**	22.6 ± 1.9	9.5 ± 0.3

## Data Availability

Raw datasets are available from the authors upon request.

## Supplementary Materials

The following are available online at https://www.mdpi.com/article/10.3390/antiox11091832/s1, Figures S1–S7: HPLC-PDA fingerprints of oxidized product mixtures Ox1–Ox7. Figures S8–S15: HRMS spectra of compounds **2**–**8**. Figures S16–S56: 1D and 2D NMR spectra of compounds **2**–**9**. Figures S57–S61: Lineweaver–Burk plots of compounds **2**–**6**.
