# Correction: Agbadua et al. Oxidized Resveratrol Metabolites as Potent Antioxidants and Xanthine Oxidase Inhibitors. *Antioxidants* 2022, *11*, 1832

**DOI:** 10.3390/antiox13101206

**Published:** 2024-10-08

**Authors:** Orinamhe G. Agbadua, Norbert Kúsz, Róbert Berkecz, Tamás Gáti, Gábor Tóth, Attila Hunyadi

**Affiliations:** 1 Institute of Pharmacognosy, University of Szeged, H-6720 Szeged, Hungary; 2 Institute of Pharmaceutical Analysis, University of Szeged, H-6720 Szeged, Hungary; 3 Servier Research Institute of Medicinal Chemistry (SRIMC), H-1031 Budapest, Hungary; 4 NMR Group, Department of Inorganic and Analytical Chemistry, Budapest University of Technology and Economics, H-1111 Budapest, Hungary; 5 Interdisciplinary Centre of Natural Products, University of Szeged, H-6720 Szeged, Hungary

## Text Correction

There was an error in the original publication (Section 2.6) [1]: Dry residue of the combined organic layers was purified by preparative HLPC on a biphenyl column with an isocratic elution of CH_3_CN-H_2_O (31:69, *v*/*v*) to produce compounds **7** (24.80 mg), **8** (13.30 mg), and **9** (33.98 mg) (13.30 mg). Further purification was carried out on the compounds on the same column but using an elution of CH_3_OH-H_2_O (52:48, *v*/*v*) to obtain compounds **7** (11.93 mg) and **9** (22.62 mg). Compound **8** was further purified by HPLC on a Luna Silica column (250 × 4.6 mm, 5 µm, 100 Å) using an elution of cyclohexane-isopropanol (85:15, *v*/*v*) to obtain 8.38 mg of pure compound.

A correction has been made to Section 2.6, Reaction with PIFA in Ethanol (Ox4):

The dry residue of the combined organic layers was purified by preparative HLPC on a biphenyl column with an isocratic elution of CH_3_CN-H_2_O (31:69, *v*/*v*) to produce compounds **7** (24.80 mg), **8** (33.98 mg), and **9** (13.30 mg). Further purification was carried out on the compounds on the same column but using an elution of CH_3_OH-H_2_O (52:48, *v*/*v*) to obtain compounds **7** (11.93 mg) and **8** (22.62 mg). Compound **9** was further purified by HPLC on a Luna Silica column (250 × 4.6 mm, 5 µm, 100 Å) using an elution of cyclohexane-isopropanol (85:15, *v*/*v*) to obtain 8.38 mg of pure compound.

There was an error in the original publication (Section 2.7) [1]: 2.7. Reaction of Resveratrol with FeCl_3_ and Oxone in Ethanol (Ox5).

A correction has been made to the title of Section 2.7, Reaction of Resveratrol with FeCl_3_ and Oxone in Ethanol (Ox5):

2.7. Reaction of Resveratrol with Periodic Acid and Oxone in Ethanol (Ox5).

There was an error in the original publication (Section 3.2) [1]: Compound **5**, i.e., (*E*)-(±)-2,3-*cis*-δ-viniferin, is structurally (±)-(*E*)-5-(3,5-dihydroxystyryl)-3-(3,5-dihydroxyphenyl)-2-(4-hydroxyphenyl)-*cis*-dihydrobenzofuran.

A correction has been made to Section 3.2, Structure Elucidation of the Isolated Compounds, Paragraph Number 5:

Compound **5**, i.e., (*E*)-(±)-2,3-*trans*-δ-viniferin, is structurally (±)-(*E*)-5-(3,5-dihydroxystyryl)-3-(3,5-dihydroxyphenyl)-2-(4-hydroxyphenyl)-*trans*-dihydrobenzofuran.

There was an error in the original publication (Section 3.2) [1]: Considering the approximately planar structure of the five-membered ring of dihydrobenzofurans, in the *cis* isomer (compound **5**), a dihedral angle close to zero degrees is consistent with a *J*(H-2,H-3) = 8 Hz coupling. On the other hand, in the case of *trans* substituents, the detected *J*(H-2,H-3) = 5 Hz coupling is in accordance with a ≈ 120° dihedral angle (see compound **6**).

A correction has been made to Section 3.2, Structure Elucidation of the Isolated Compounds, Paragraph Number 5:

Even though both compounds **5** and **6** are *trans*-substituted at the 2,3 positions of the nearly planar dihydrobenzofuran ring, the peri-effect, caused by the 4 substituent of compound **6**, alters the geometry of the dominant conformer as compared to that of compound **5**. This manifests in differences in the *J*(H-2,H-3) coupling constants, i.e., 8 Hz and 5 Hz for compounds **5** and **6**, respectively.

## Error in Figure (in the Main Text)

In the original publication [1], there was a mistake in “Figure 1. Structures of resveratrol (**1**) and its metabolites obtained by chemical oxidation (**2**–**9**). Each optically active compound (**5**, **6**, **8**, **9**) is racemate; for simplicity, only one enantiomer is presented. For compounds **7** and **9**, the relative configuration could not be determined” as published.

In Section 3.2, the stereochemistry of compound **5** was erroneously assigned as (*E*)-(±)-2,3-*cis*-δ-viniferin. The subsequent extensive spectroscopic analysis and high-level in silico quantum chemical calculations revealed this compound as a *trans*-isomer. The structure and stereochemistry of compound **5** were confirmed by comparing with the ^1^H-NMR spectroscopic data of *trans*-δ-viniferin previously reported by Huber and colleagues [2]. Figure 1 should be corrected as follows due to an erroneous additional -OH group added to the structure of **6** in the previously published article. The corrected “Figure 1. Structures of resveratrol (**1**) and its metabolites obtained by chemical oxidation (**2**–**9**). Each optically active compound (**5**, **6**, **8**, **9**) is racemate; for simplicity, only one enantiomer is presented. For compounds **7** and **9**, the relative configuration could not be determined” appears below.

## Error in Figure (in Supplementary Materials)

In the original publication [1], there was a mistake in “Figure S4. HPLC-PDA fingerprint of oxidized product mixture Ox4” as published. The numbers **8** and **9** were placed wrongly. The corrected “Figure S4. HPLC-PDA fingerprint of oxidized product mixture Ox4” appears below.

**Figure S4:**
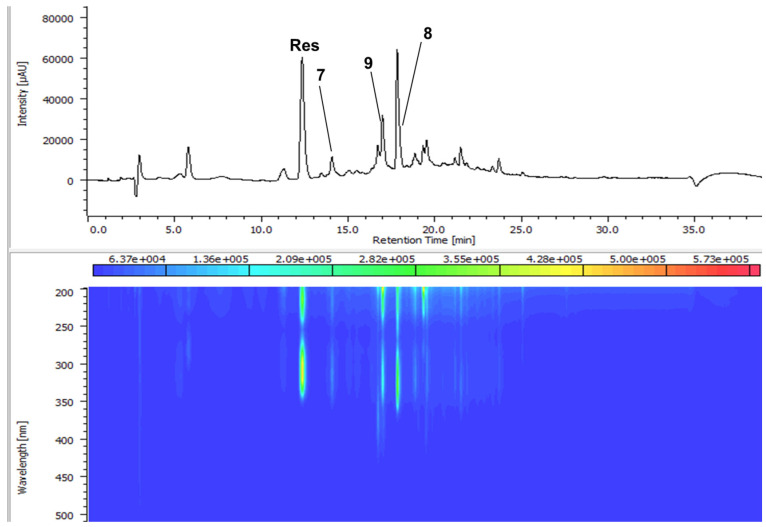
HPLC-PDA fingerprint of oxidized product mixture Ox4.

In the original publication [1], there was a mistake in “Figure S12. Compound **5**, HRMS (positive mode)” as published. The structure of **5** needs to be revised. The corrected “Figure S12. Compound **5**, HRMS (positive mode)” appears below.

**Figure S12:**
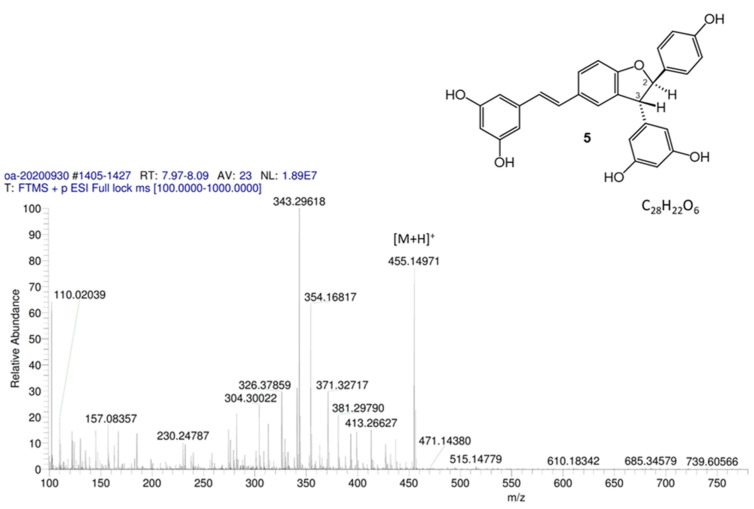
Compound **5**, HRMS (positive mode).

In the original publication [1], there was a mistake in “Figure S28. Compound **5**, ^1^H NMR spectrum, and selROE on δ4.47 and δ5.45 ppm” as published. All stereostructures of compound **5** in Supplementary Figures S28–S32 should be revised. The corrected “Figure S28. Compound **5**, ^1^H NMR spectrum, and selROE on δ4.47 and δ5.45 ppm” appears below.

**Figure S28:**
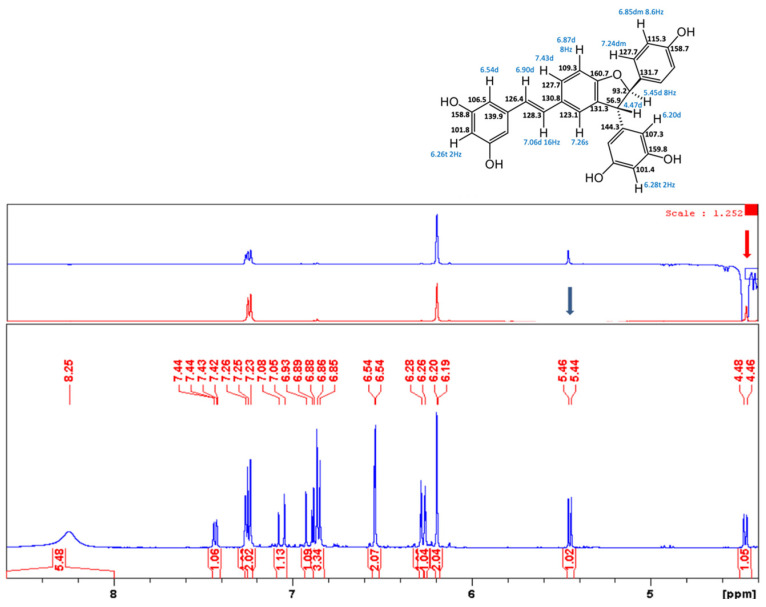
Compound **5**, ^1^H NMR spectrum, and selROE on δ4.47 and δ5.45 ppm.

In the original publication [1], there was a mistake in “Figure S29. Compound **5**, ^13^C, APT NMR spectrum” as published. All stereostructures of compound **5** in Supplementary Figures S28–S32 should be revised. The corrected “Figure S29. Compound **5**, ^13^C, APT NMR spectrum” appears below.

**Figure S29:**
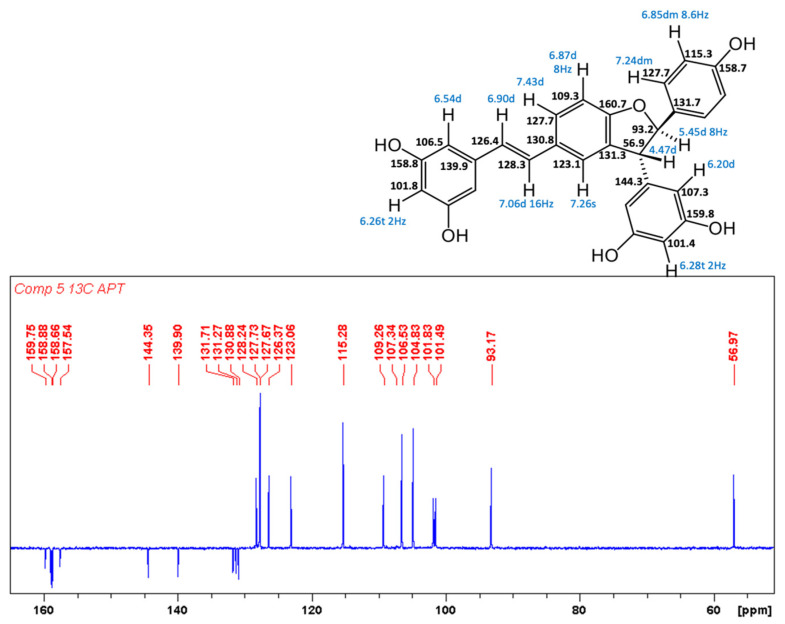
Compound **5**, ^13^C, APT NMR spectrum.

In the original publication [1], there was a mistake in “Figure S30. Compound **5**, HSQC spectrum and HSQC section” as published. All stereostructures of compound **5** in Supplementary Figures S28–S32 should be revised. The corrected “Figure S30. Compound **5**, HSQC spectrum and HSQC section” appears below.

**Figure S30:**
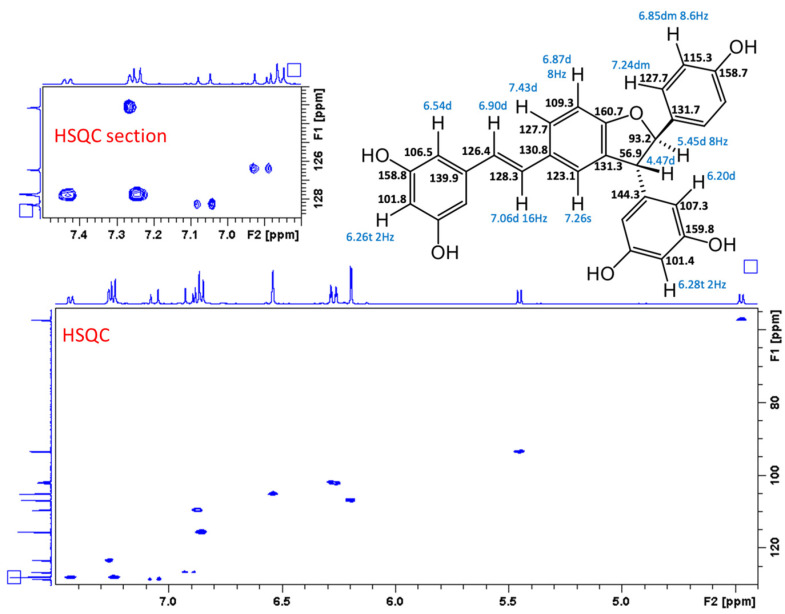
Compound **5**, HSQC spectrum and HSQC section.

In the original publication [1], there was a mistake in “Figure S31. Compound **5**, COSY spectrum” as published. All stereostructures of compound **5** in Supplementary Figures S28–S32 should be revised. The corrected “Figure S31. Compound **5**, COSY spectrum” appears below.

**Figure S31:**
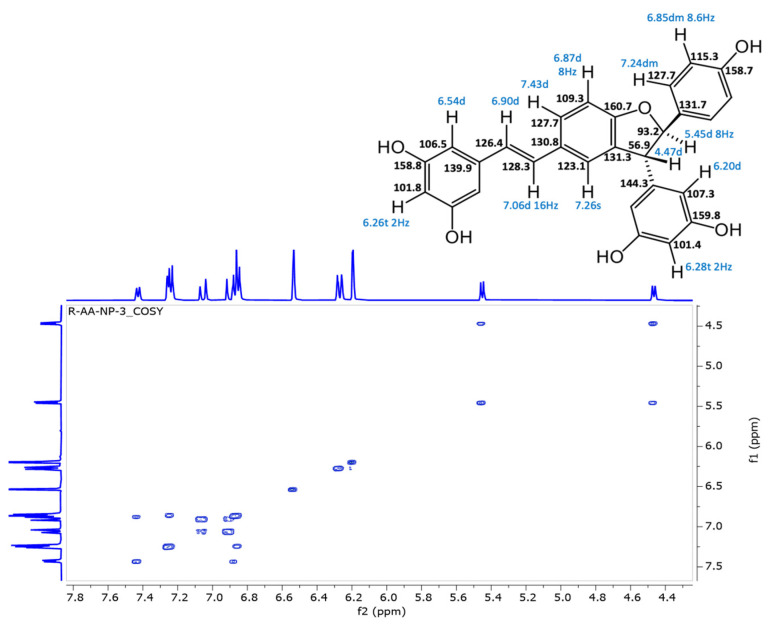
Compound **5,** COSY spectrum.

In the original publication [1], there was a mistake in “Figure S32. Compound **5**, HMBC spectrum” as published. All stereostructures of compound **5** in Supplementary Figures S28–S32 should be revised. The corrected “Figure S32. Compound **5**, HMBC spectrum” appears below.

**Figure S32:**
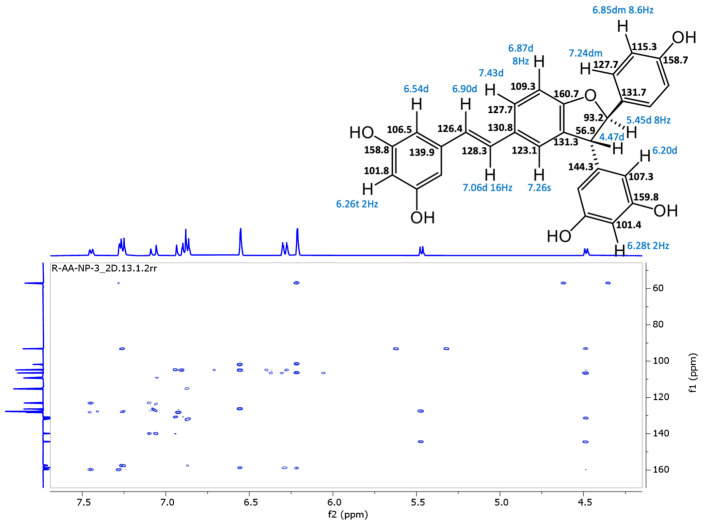
Compound **5**, HMBC spectrum.

The authors state that the scientific conclusions are unaffected. This correction was approved by the Academic Editor. The original publication has also been updated.

## Figures and Tables

**Figure 1:**
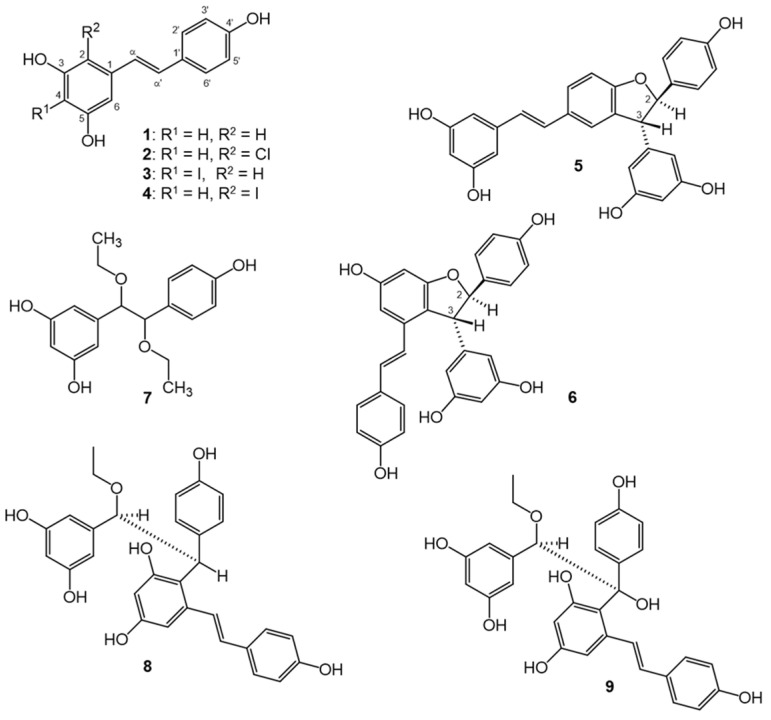
Structures of resveratrol (**1**) and its metabolites obtained by chemical oxidation (**2**–**9**). Each optically active compound (**5**, **6**, **8**, **9**) is racemate; for simplicity, only one enantiomer is presented. For compounds **7** and **9**, the relative configuration could not be determined.
