# Supplementary material for: Oxidized Resveratrol Metabolites as Potent Antioxidants and Xanthine Oxidase Inhibitors
Source: Antioxidants (Basel). 2022 Sep 17;11(9):1832. doi: 10.3390/antiox11091832 (PMC9495788; doi:10.3390/antiox11091832)
Supplement: Supplementary file 1 [file antioxidants-11-01832-s001.zip › antioxidants-1875141-supplementary.pdf]

Supplementary Materials to
Oxidized resveratrol metabolites as potent antioxidants and xanthine oxidase inhibitors

Orinamhe G. Agbadua[1], Norbert Kúsz [1], Róbert Berkecz [2], Tamás Gáti [3], Gábor Tóth [4], and Attila Hunyadi [1,5,*]

[1] Institute of Pharmacognosy, University of Szeged, H-6720, Szeged, Hungary
[2] Institute of Pharmaceutical Analysis, University of Szeged, H-6720 Szeged, Hungary
[3] Servier Research Institute of Medicinal Chemistry (SRIMC), H-1031 Budapest, Hungary
[4] NMR Group, Department of Inorganic and Analytical Chemistry, Budapest University of Technology and Economics, H-1111 Budapest, Hungary
[5] Interdisciplinary Centre of Natural Products, University of Szeged, H-6720 Szeged, Hungary
* Correspondence: hunyadi.attila@szte.hu; Tel.: +36-62546456

Table of contents

Figure S1. HPLC-PDA fingerprint of oxidized product mixture Ox1.
Figure S2. HPLC-PDA fingerprint of oxidized product mixture Ox2.
Figure S3. HPLC-PDA fingerprint of oxidized product mixture Ox3.
Figure S4. HPLC-PDA fingerprint of oxidized product mixture Ox4.
Figure S5. HPLC-PDA fingerprint of oxidized product mixture Ox5.
Figure S6. HPLC-PDA fingerprint of oxidized product mixture Ox6.
Figure S7. HPLC-PDA fingerprint of oxidized product mixture Ox7.
Figure S8. Compound **2**, HRMS (positive mode).
Figure S9. Compound **2**, HRMS (negative mode).
Figure S10. Compound **3**, HRMS (negative mode).
Figure S11. Compound **4**, HRMS (positive mode).
Figure S12. Compound **5**, HRMS (positive mode).
Figure S13. Compound **6**, HRMS (positive mode).
Figure S14. Compound **7**, HRMS (negative mode).
Figure S15. Compound **8**, HRMS (positive mode).
Figure S16. Compound **2**, ^{1}H NMR spectrum.
Figure S17. Compound **2**, ^{13}C, APT NMR spectrum.
Figure S18. Compound **2**, HSQC and HMBC spectra.
Figure S19. Compound **2**, COSY and NOESY spectra.

Figure S20. Compound **3**, ^{1}H NMR spectrum.
Figure S21. Compound **3**, ^{13}C, APT NMR spectrum.
Figure S22. Compound **3**, HSQC and HMBCspectra.
Figure S23. Compound **3**, COSY spectrum.
Figure S24. Compound **4**, ^{1}H NMR spectrum.
Figure S25. Compound **4**, ^{13}C, APT NMR spectrum.
Figure S26. Compound **4**, HSQC and HMBC spectra.
Figure S27. Compound **4**, COSY spectrum.
Figure S28. Compound **5**, ^{1}H NMR spectrum, and selROE on δ4.47 and δ5.45 ppm.
Figure S29. Compound **5**, ^{13}C, APT NMR spectrum.
Figure S30. Compound **5**, HSQC spectrum and HSQC section.
Figure S31. Compound **5**, COSY spectrum.
Figure S32. Compound **5**, HMBC spectrum.
Figure S33. Compound **6**, ^{1}H spectrum, and selNOE on δ4.48 and δ5.42 ppm.
Figure S34. Compound **6**, ^{13}C, APT NMR spectrum.
Figure S35. Compound **6**, HSQC spectrum.
Figure S36. Compound **6**, HMBC spectrum.
Figure S37. Compound **6**, COSY and NOESY spectra.
Figure S38. Compound **7**, ^{1}H NMR spectrum.
Figure S39. Compound **7**, ^{13}C, APT NMR spectrum.
Figure S40. Compound **7**, NOESY spectrum and NOESY section.
Figure S41. Compound **7**, HSQC and HMBC spectra.
Figure S42. Compound **7**, COSY spectrum.
Figure S43. Compound **8**, ^{1}H NMR spectrum.
Figure S44. Compound **8**, ^{13}C, APT NMR spectrum.
Figure S45. Compound **8**, HSQC spectrum and HSQC section.
Figure S46. Compound **8**, HMBC spectrum and HMBC section.
Figure S47. Compound **8**, COSY spectrum and COSY aromatic section.
Figure S48. Compound **8**, NOESY; arrows indicate steric proximities, and assign configuration and the dominant conformer.

Figure S49. Compound **9**, ^{1}H NMR spectrum.
Figure S50. Compound **9**, ^{13}C, APT NMR spectrum.
Figure S51. Compound **9**, HSQC spectrum and HSQC section.
Figure S52. Compound **9**, HMBC spectrum.
Figure S53. Compound **9**, **N**OESY spectrum.
Figure S54. Compound **9**, HSQC spectrum and HSQC section, DMSO-d_6, T=80°C.
Figure S55. Compound **9**, ^{13}C, APT NMR spectrum, DMSO-d_6, T=80°C.
Figure S56. Compound **9**, selROE on CH (5.15 ppm) and OH (4.54 ppm) , DMSO-d_6, T=80°C.
Figure S57. Lineweaver-Burk plot of compound **2**.
Figure S58. Lineweaver-Burk plot of compound **3**.
Figure S59. Lineweaver-Burk plot of compound **4**.
Figure S60. Lineweaver-Burk plot of compound **5**.
Figure S61. Lineweaver-Burk plot of compound **6**.

Figure S1. HPLC-PDA fingerprint of oxidized product mixture Ox1.

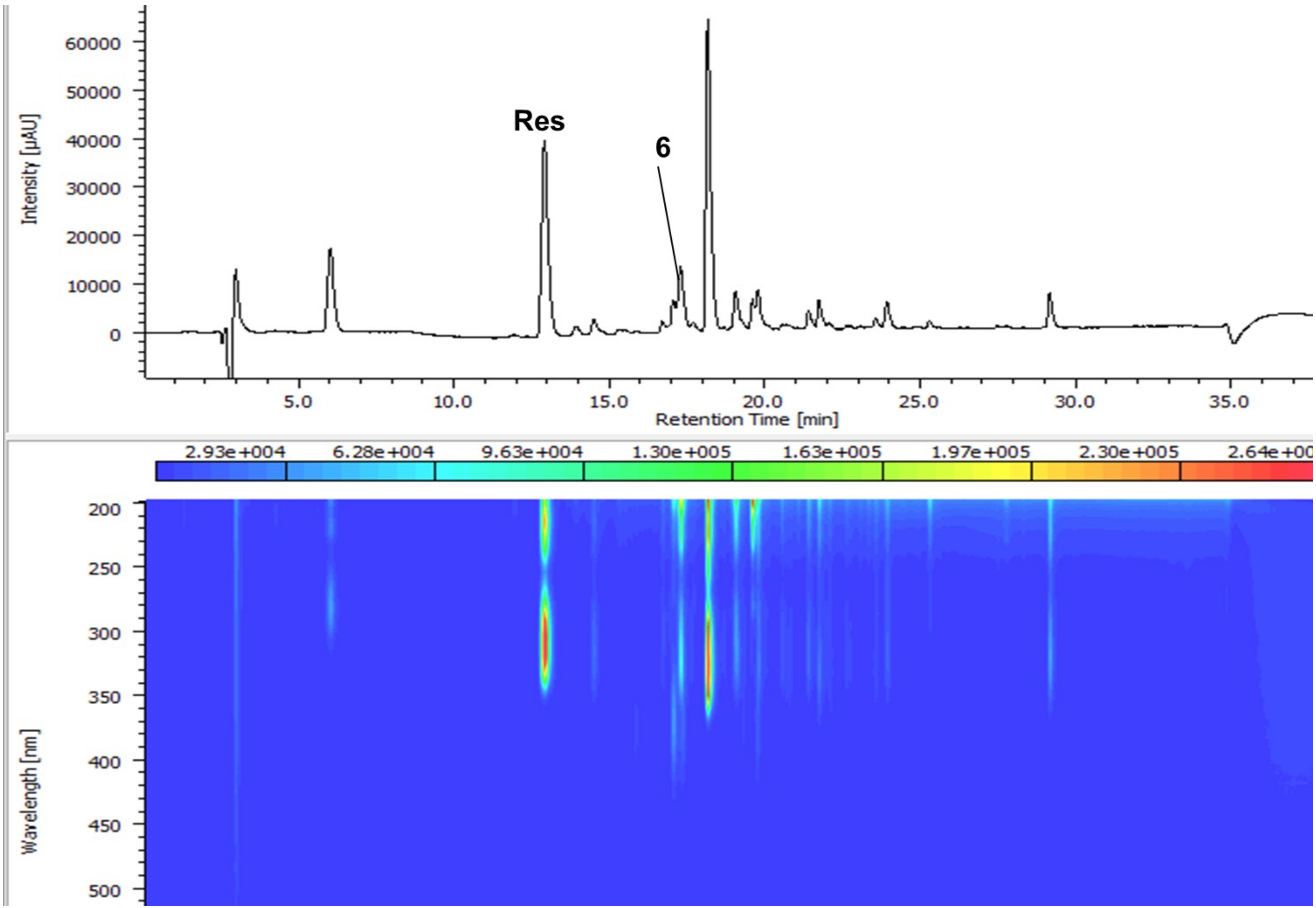

Figure S2. HPLC-PDA fingerprint of oxidized product mixture Ox2.

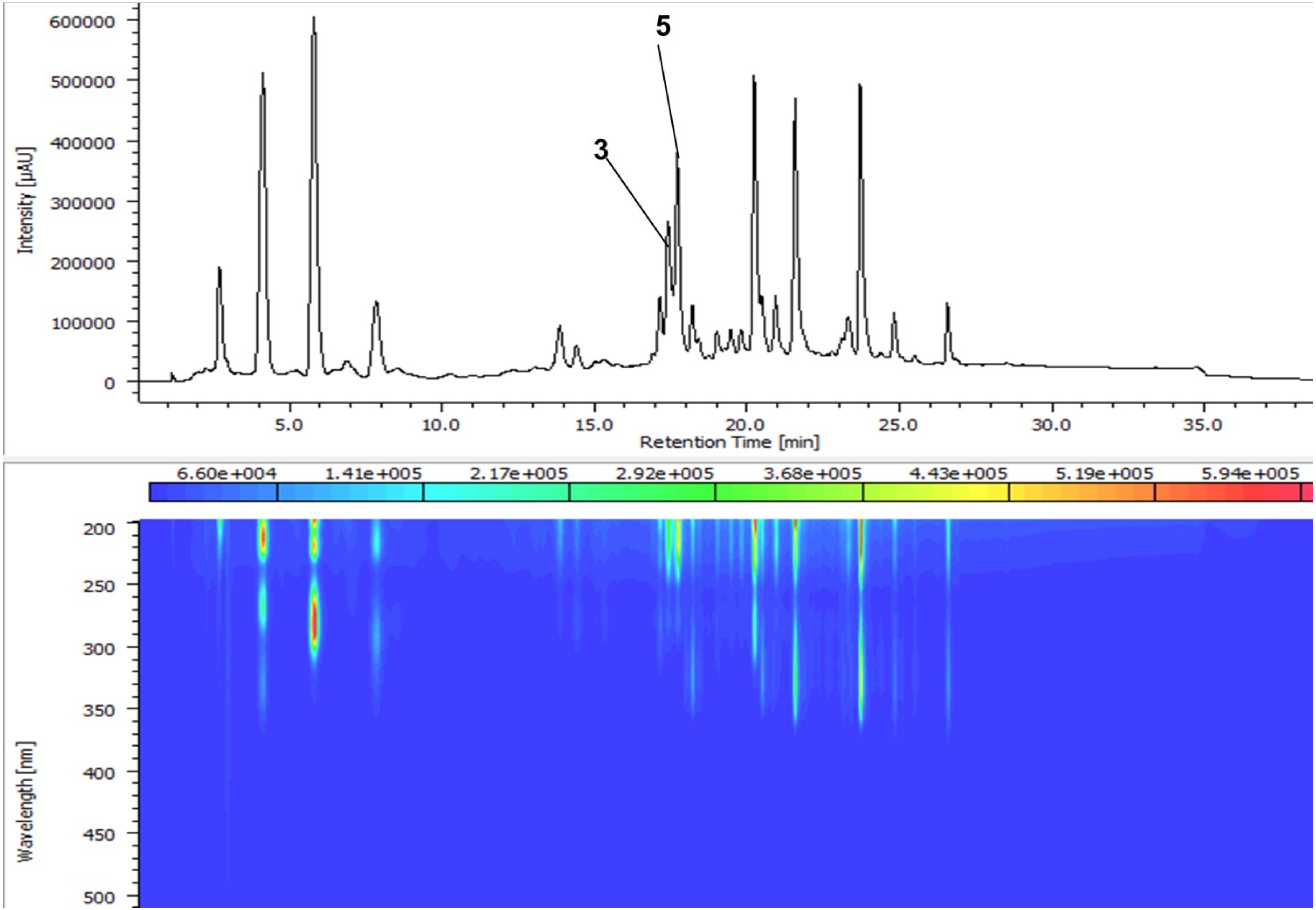

Figure S3. HPLC-PDA fingerprint of oxidized product mixture Ox3.

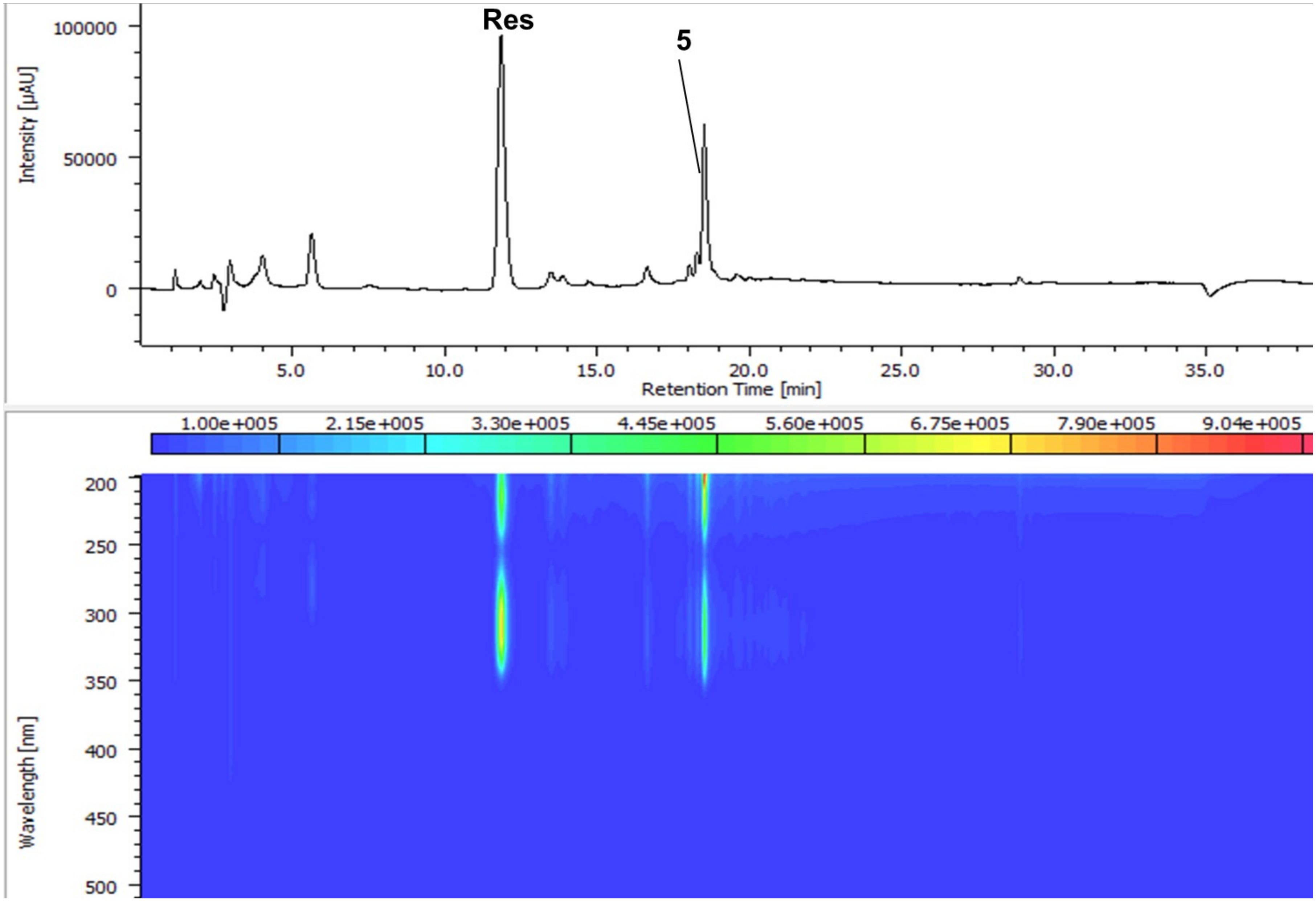

Figure S4. HPLC-PDA fingerprint of oxidized product mixture Ox4.

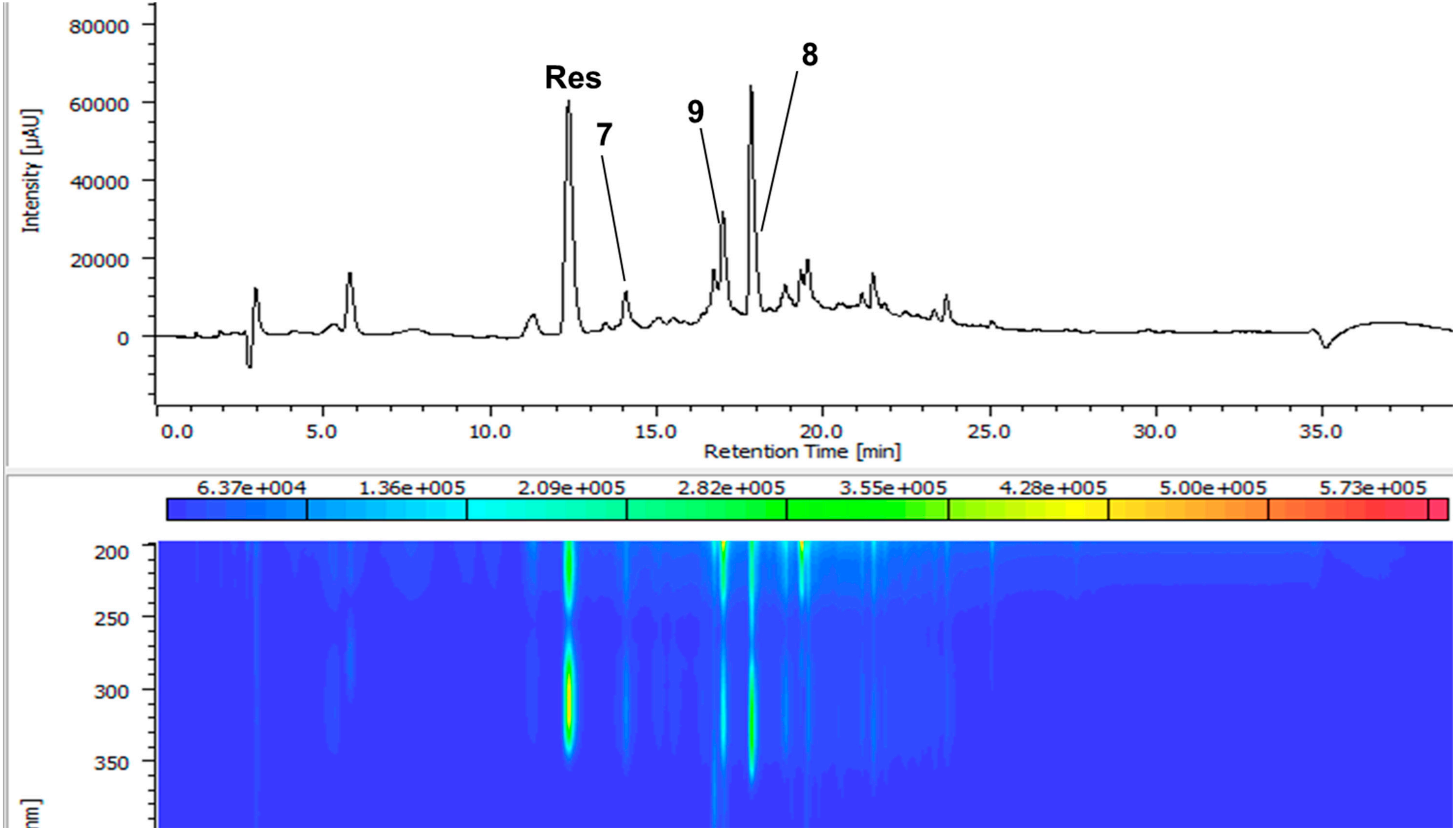

Figure S5. HPLC-PDA fingerprint of oxidized product mixture Ox5.

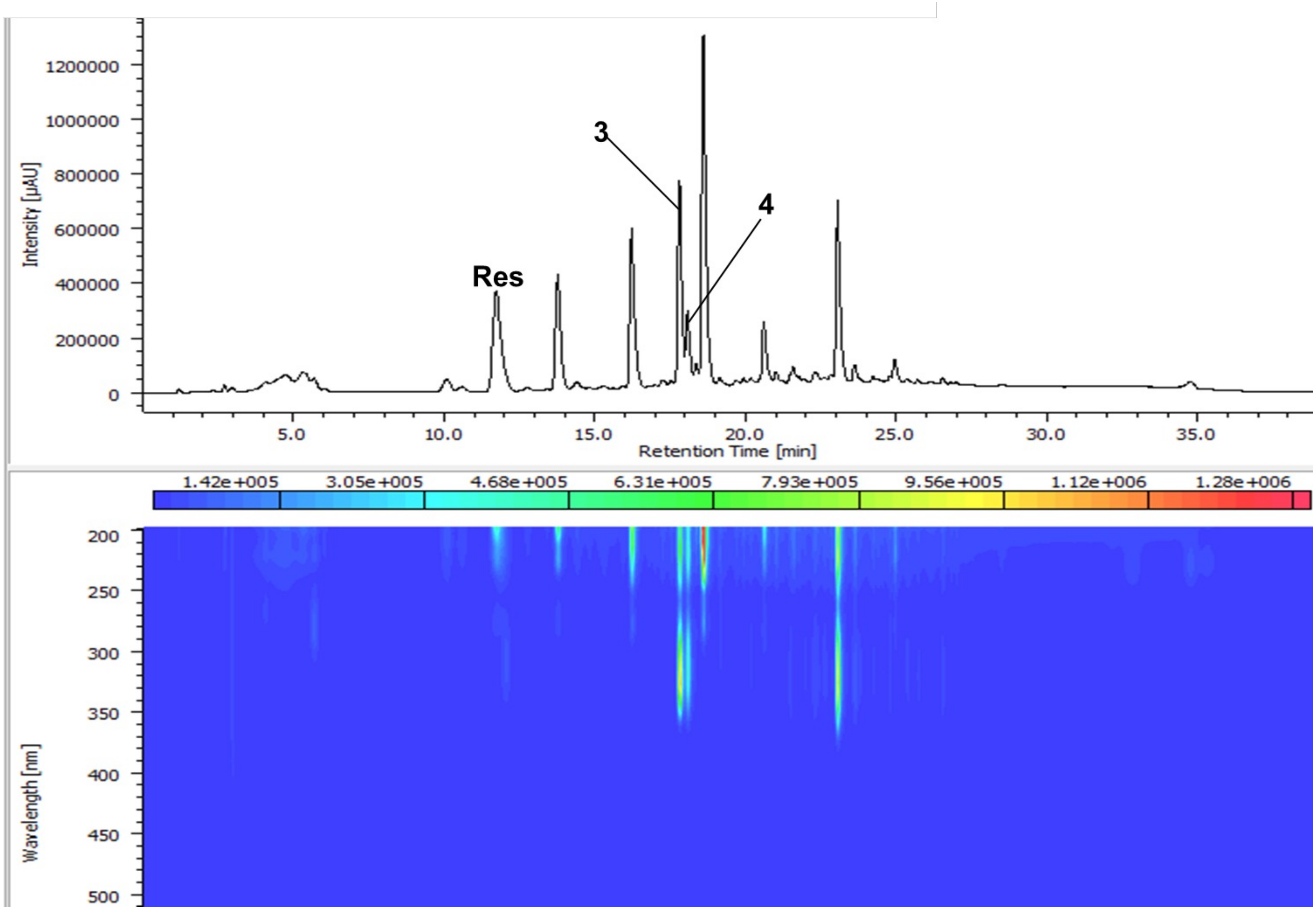

Figure S6. HPLC-PDA fingerprint of oxidized product mixture Ox6.

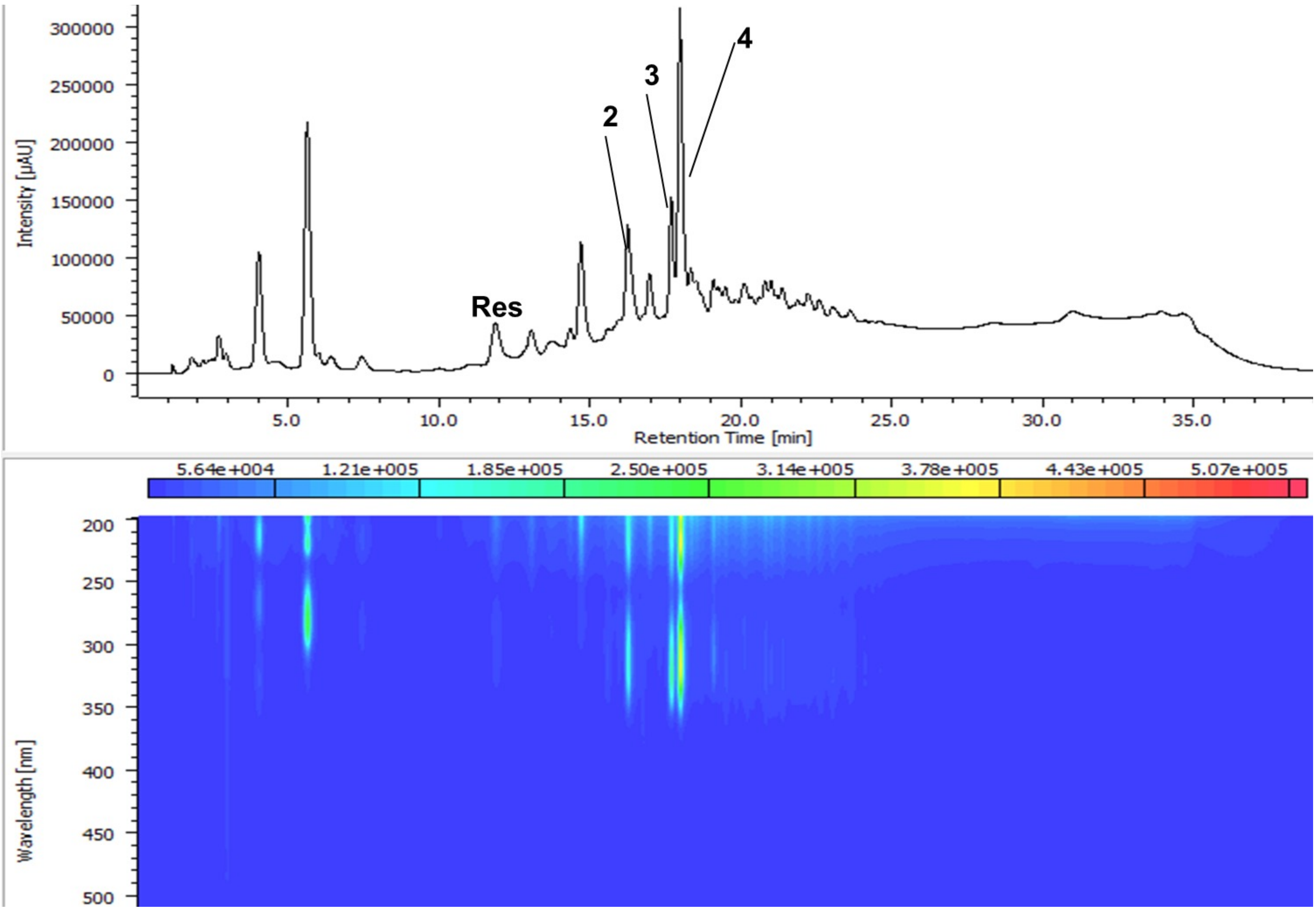

Figure S7. HPLC-PDA fingerprint of oxidized product mixture Ox7.

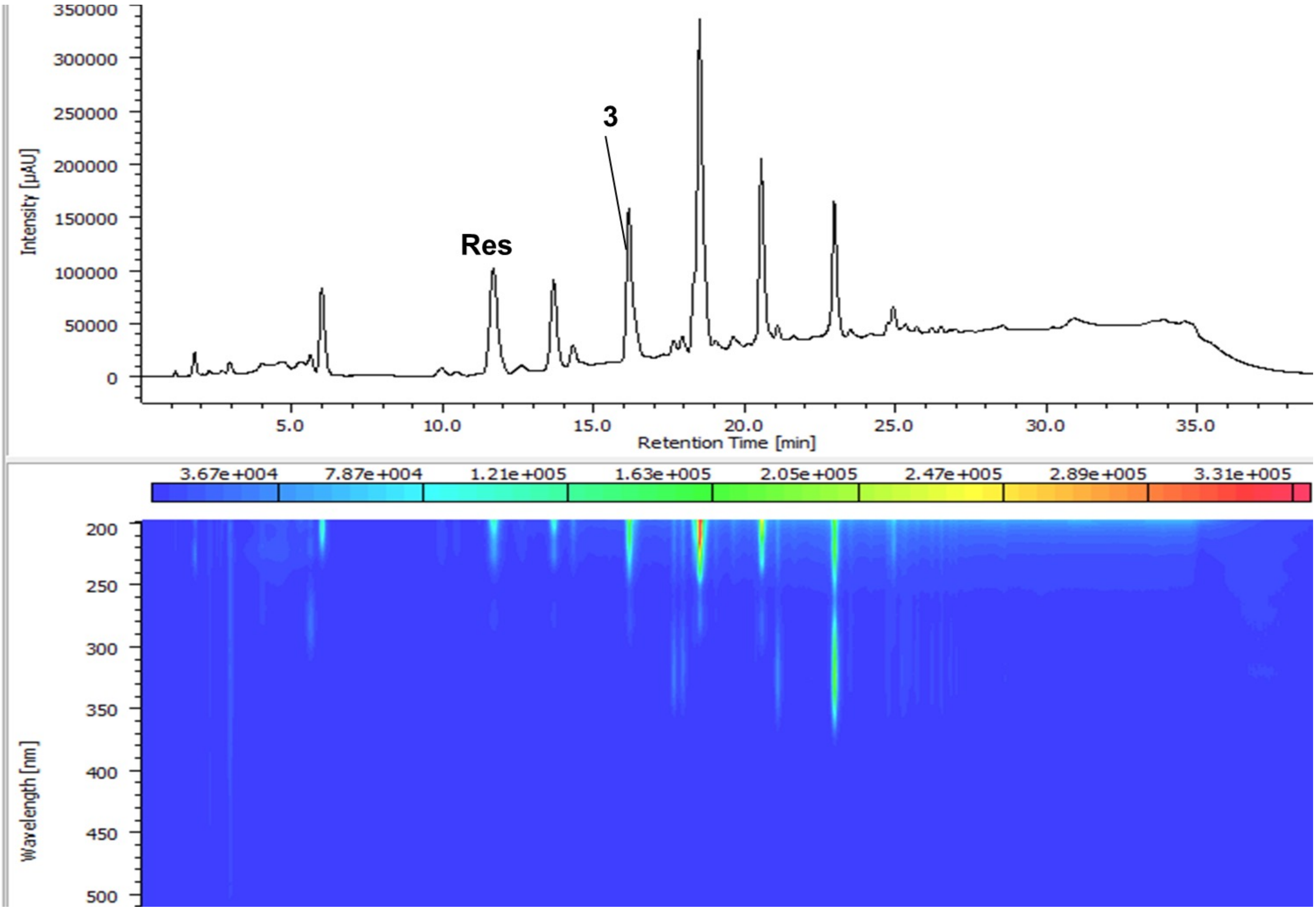

Figure S8. Compound **2**, HRMS (positive mode).

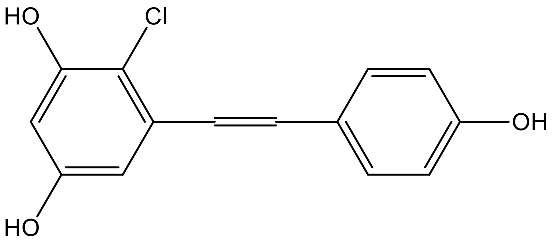

2

$C_{14}H_{11}O_3Cl$

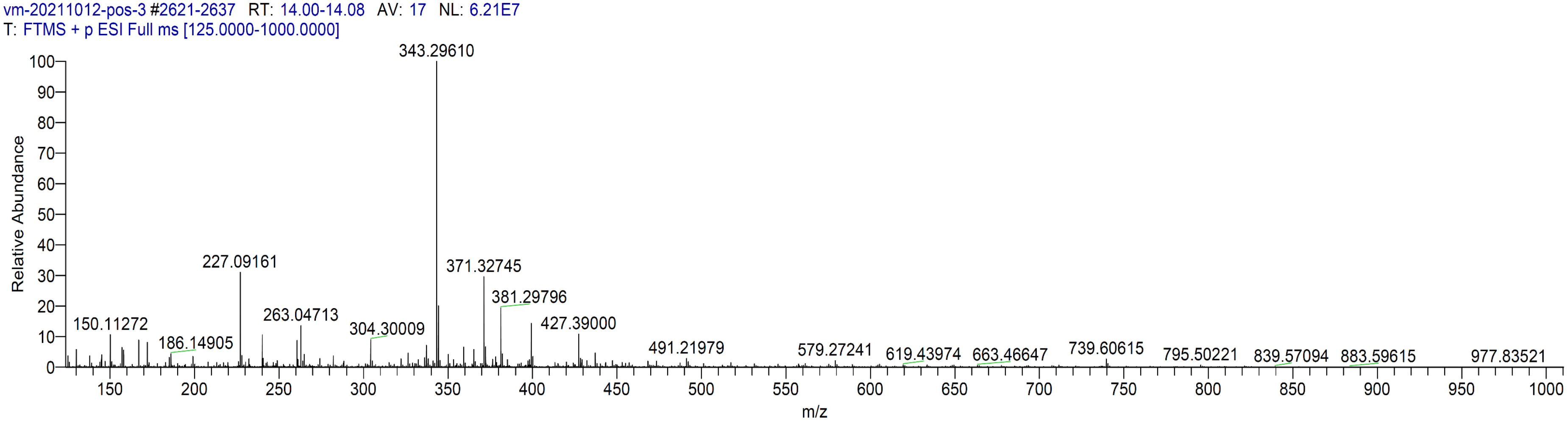

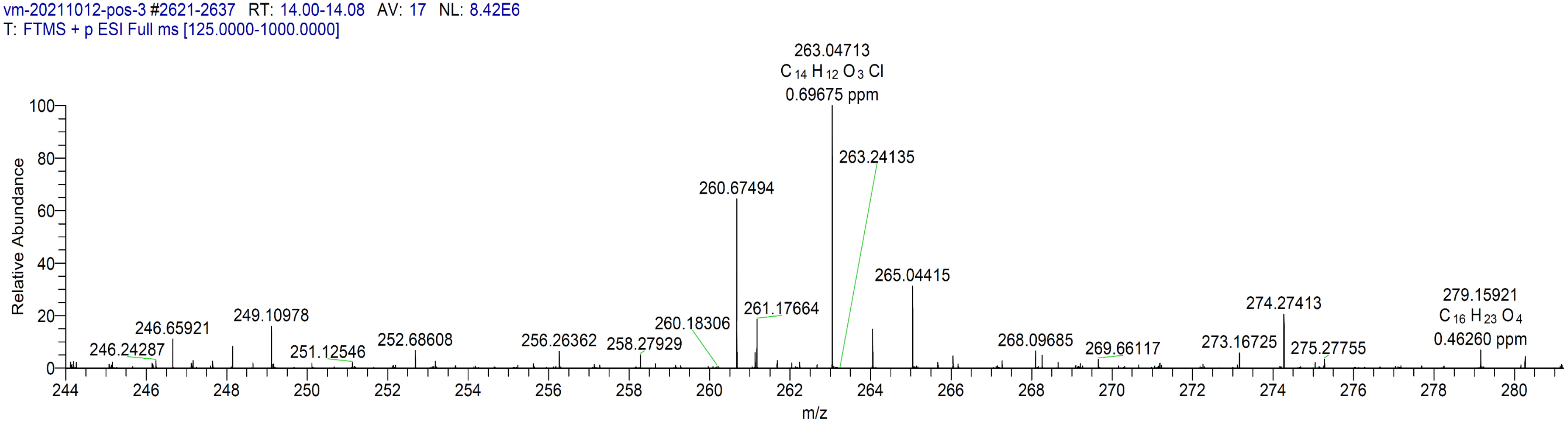

Figure S9. Compound **2**, HRMS (negative mode).

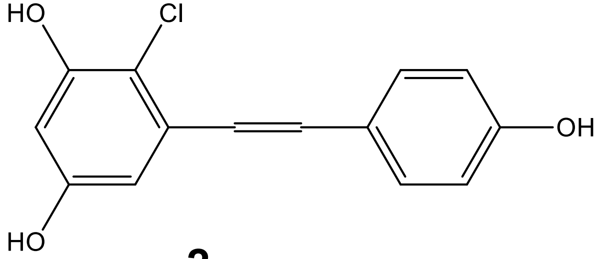

2

$C_{14}H_{11}O_3Cl$

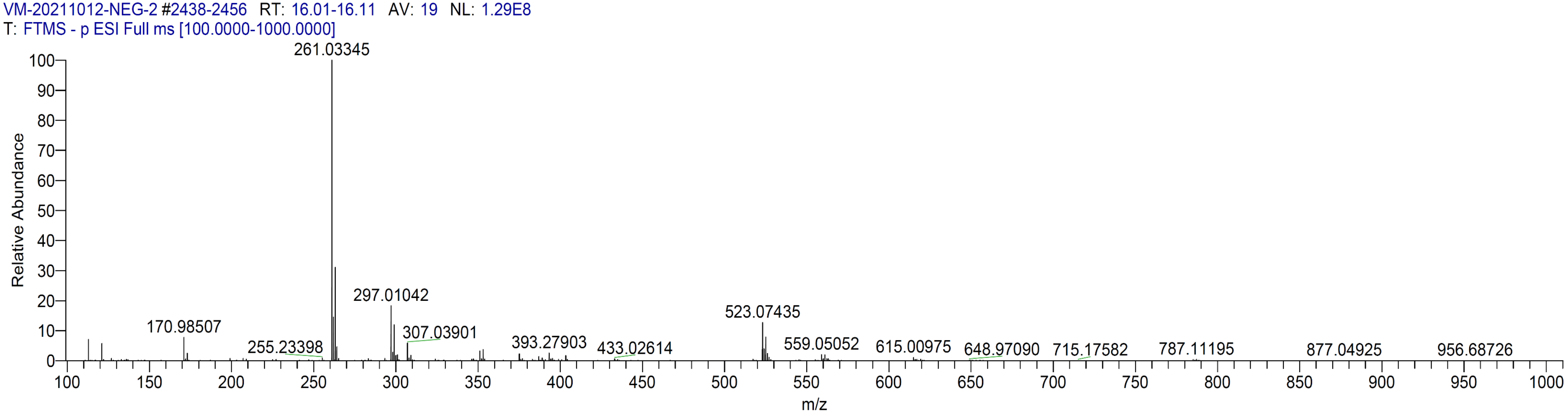

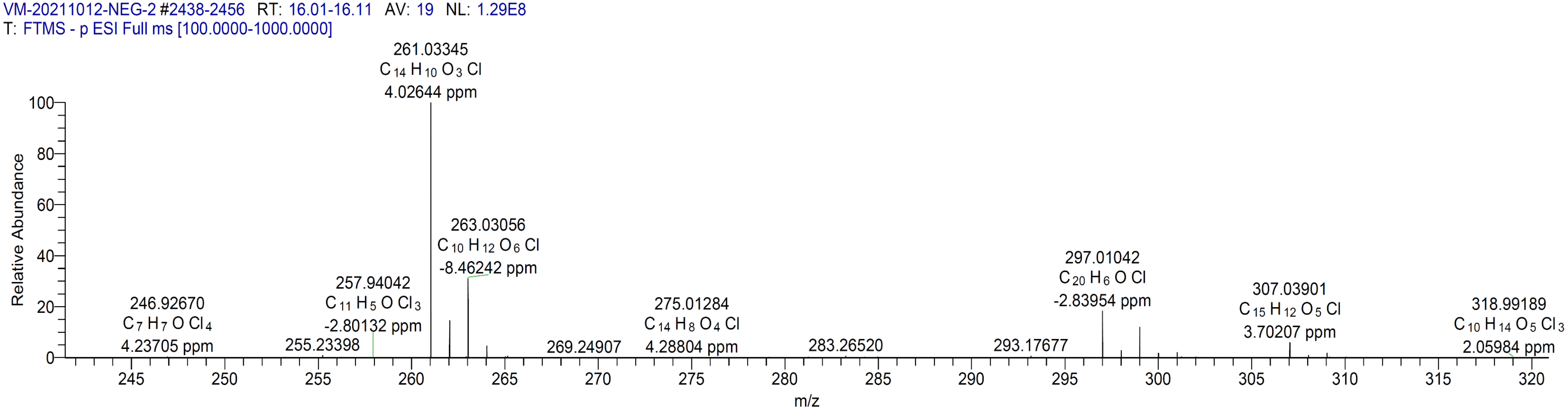

Figure S10. Compound **3**, HRMS (negative mode).

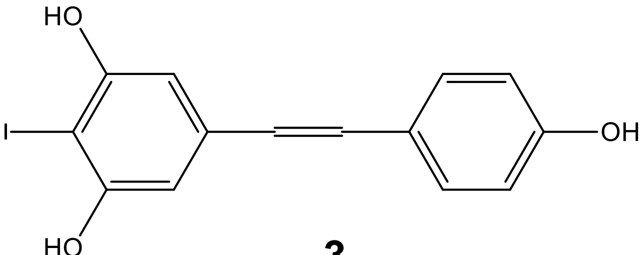

3

$C_{14}H_{11}O_3I$

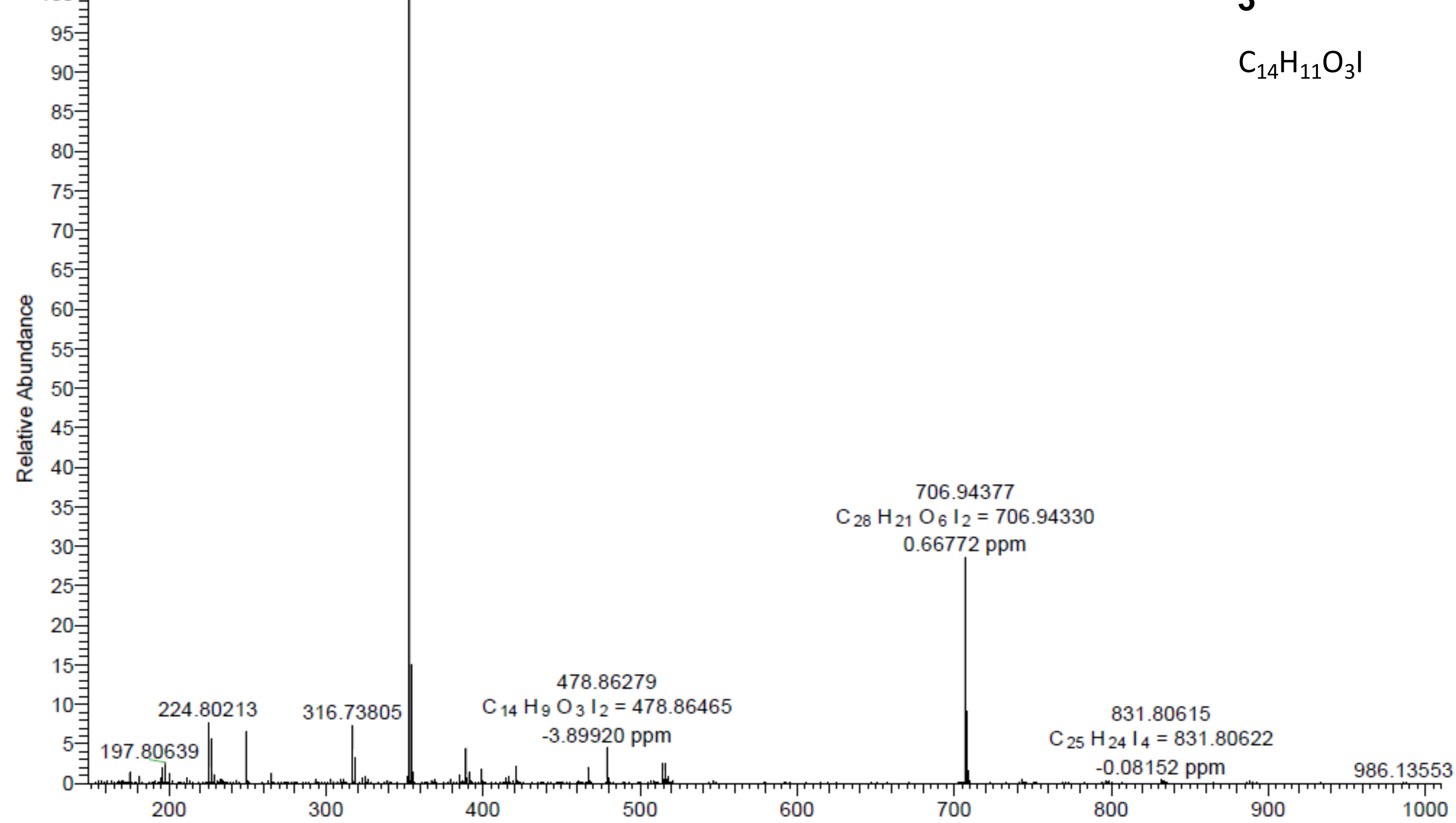

Figure S11. Compound **4**, HRMS (positive mode).

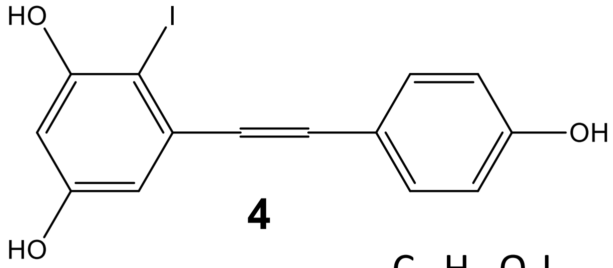

$C_{14}H_{11}O_3I$

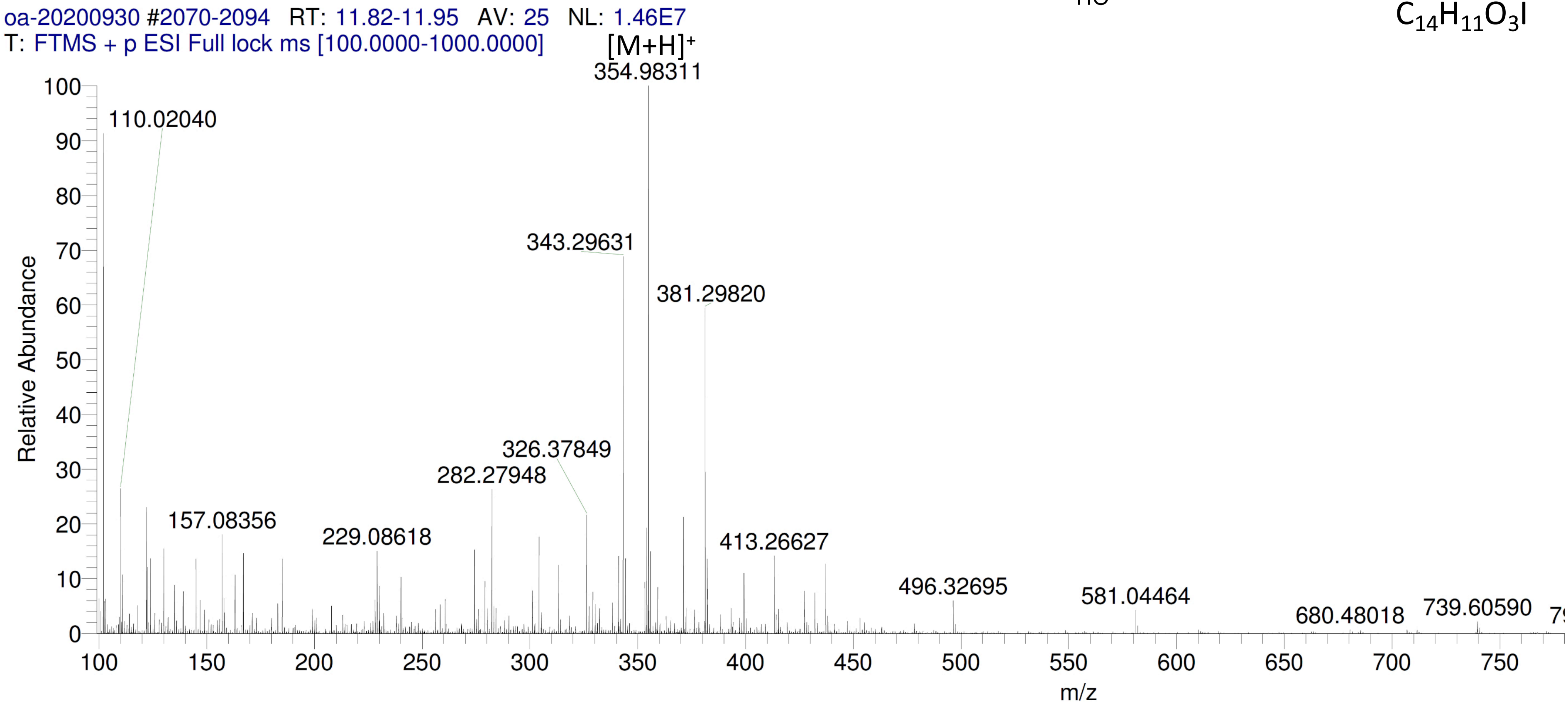

Figure S12. Compound **5**, HRMS (positive mode).

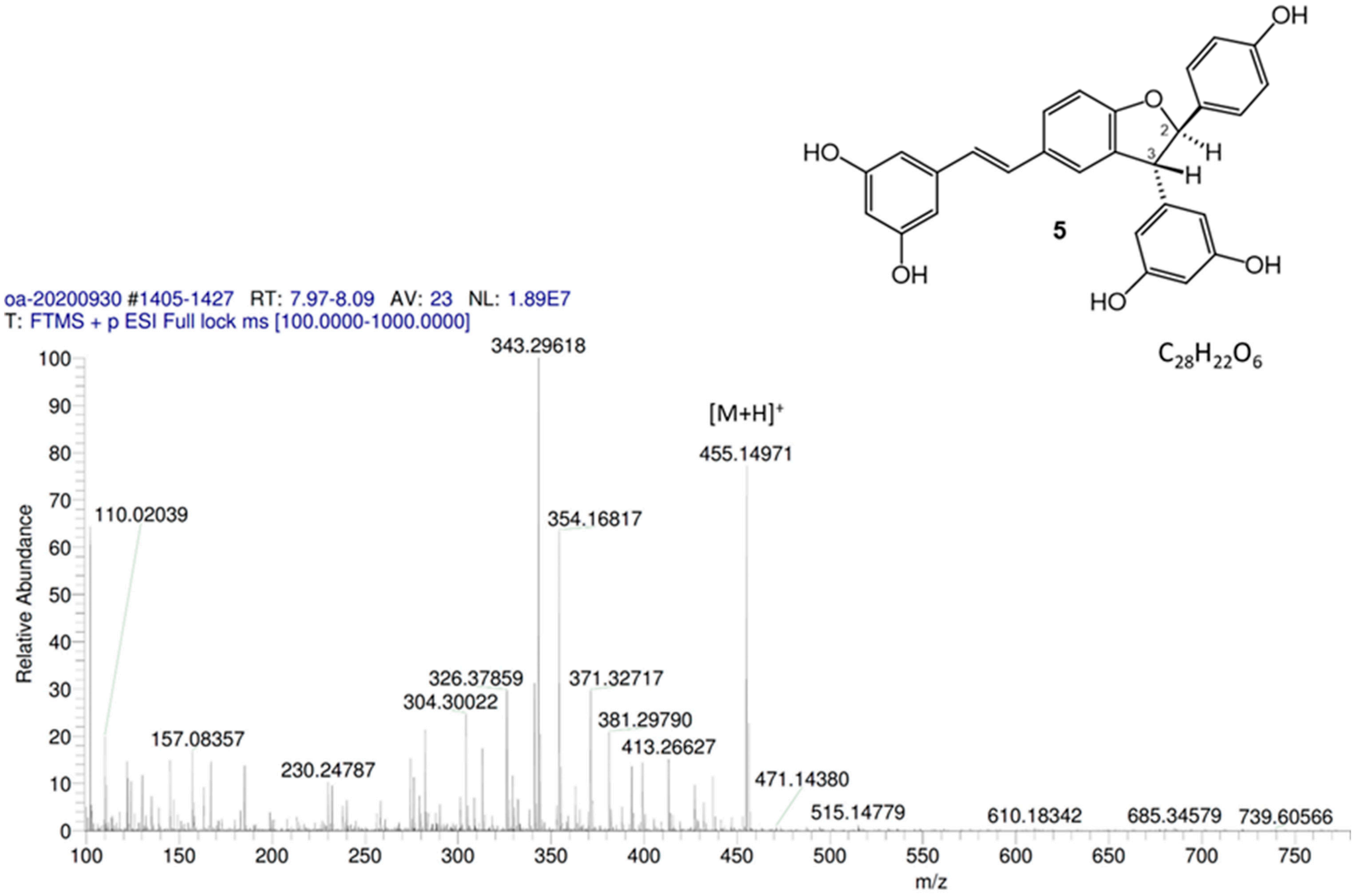

Figure S13. Compound **6**, HRMS (positive mode).

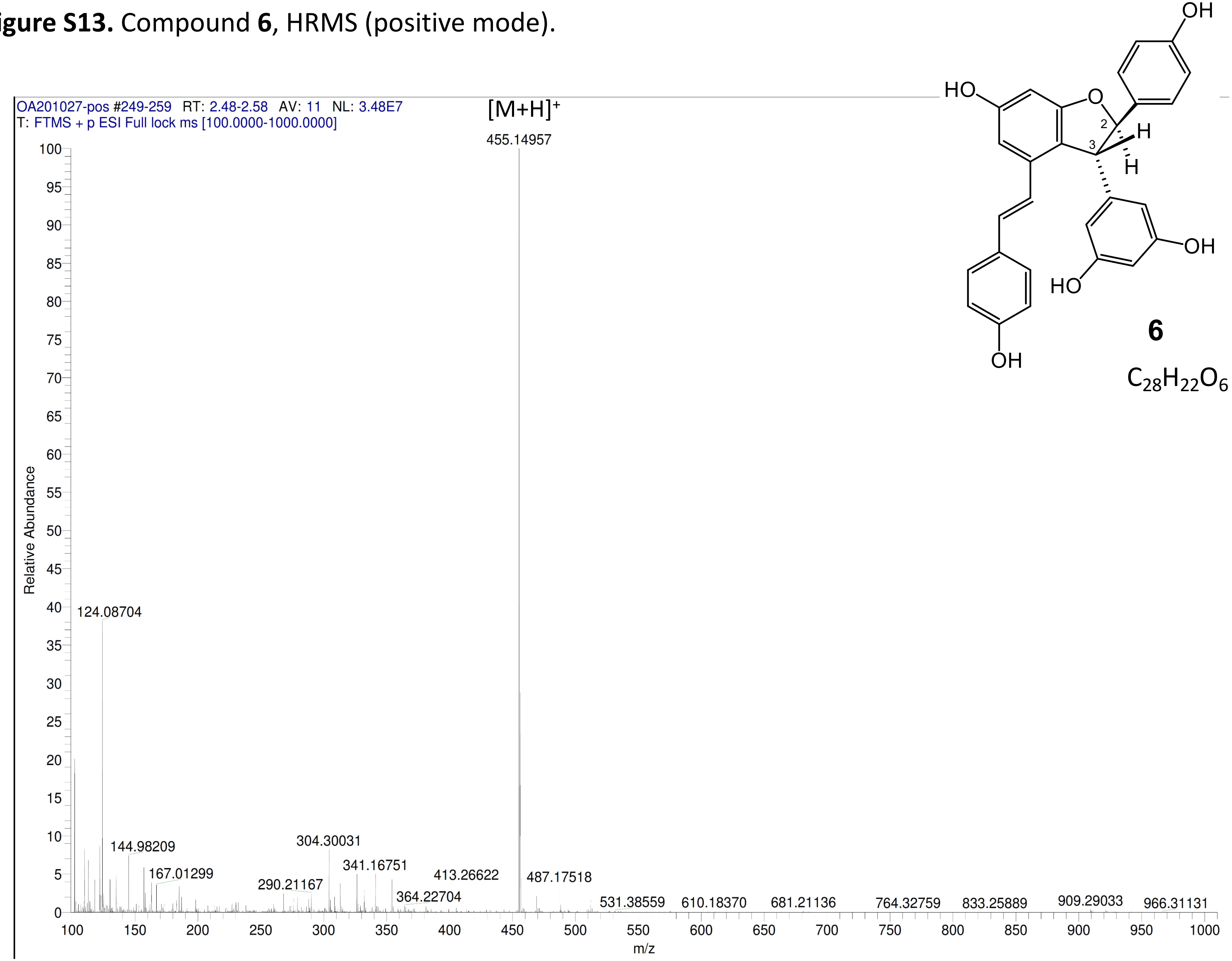

6

$C_{28}H_{22}O_6$

Figure S14. Compound **7**, HRMS (negative mode).

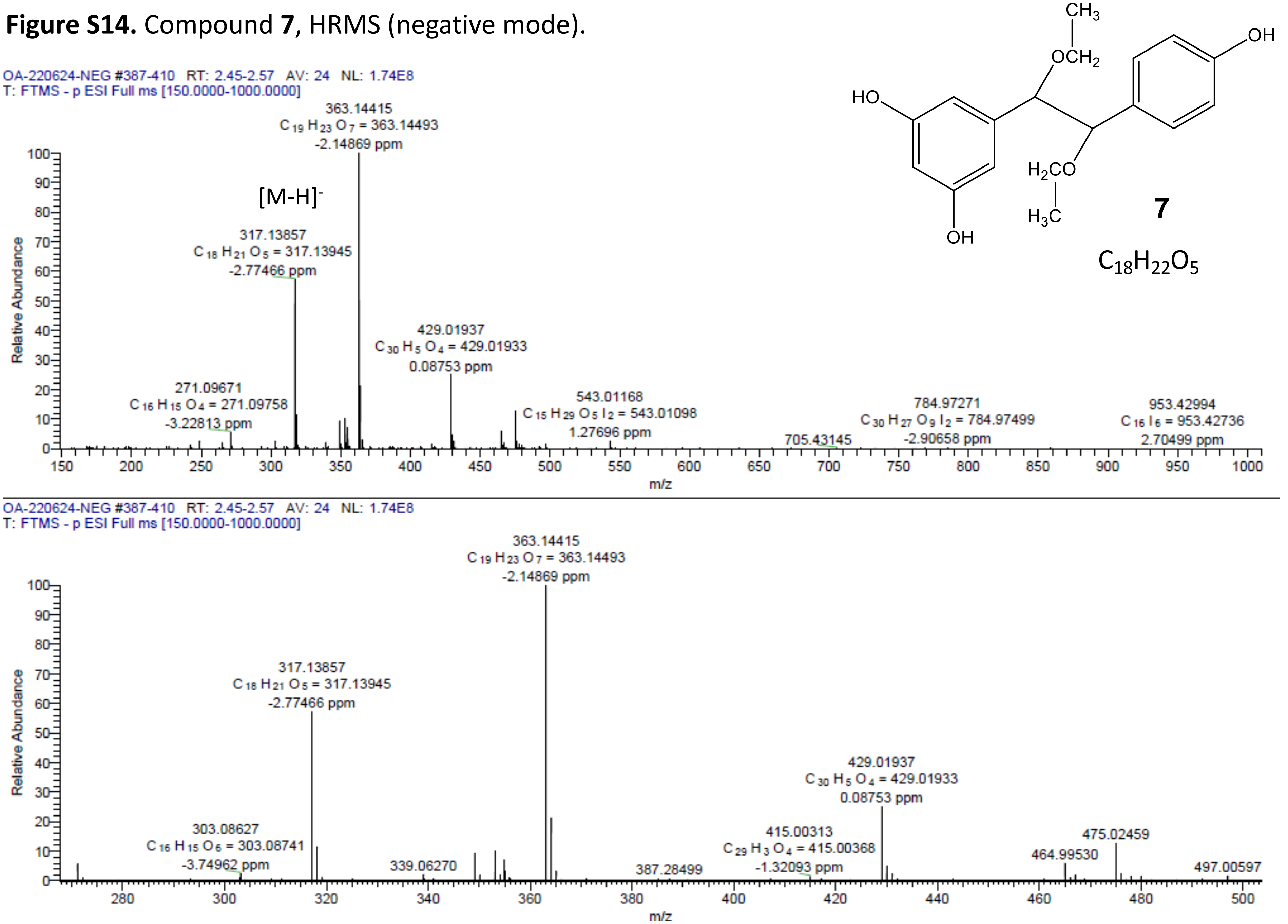

Figure S15. Compound **8**, HRMS (positive mode).

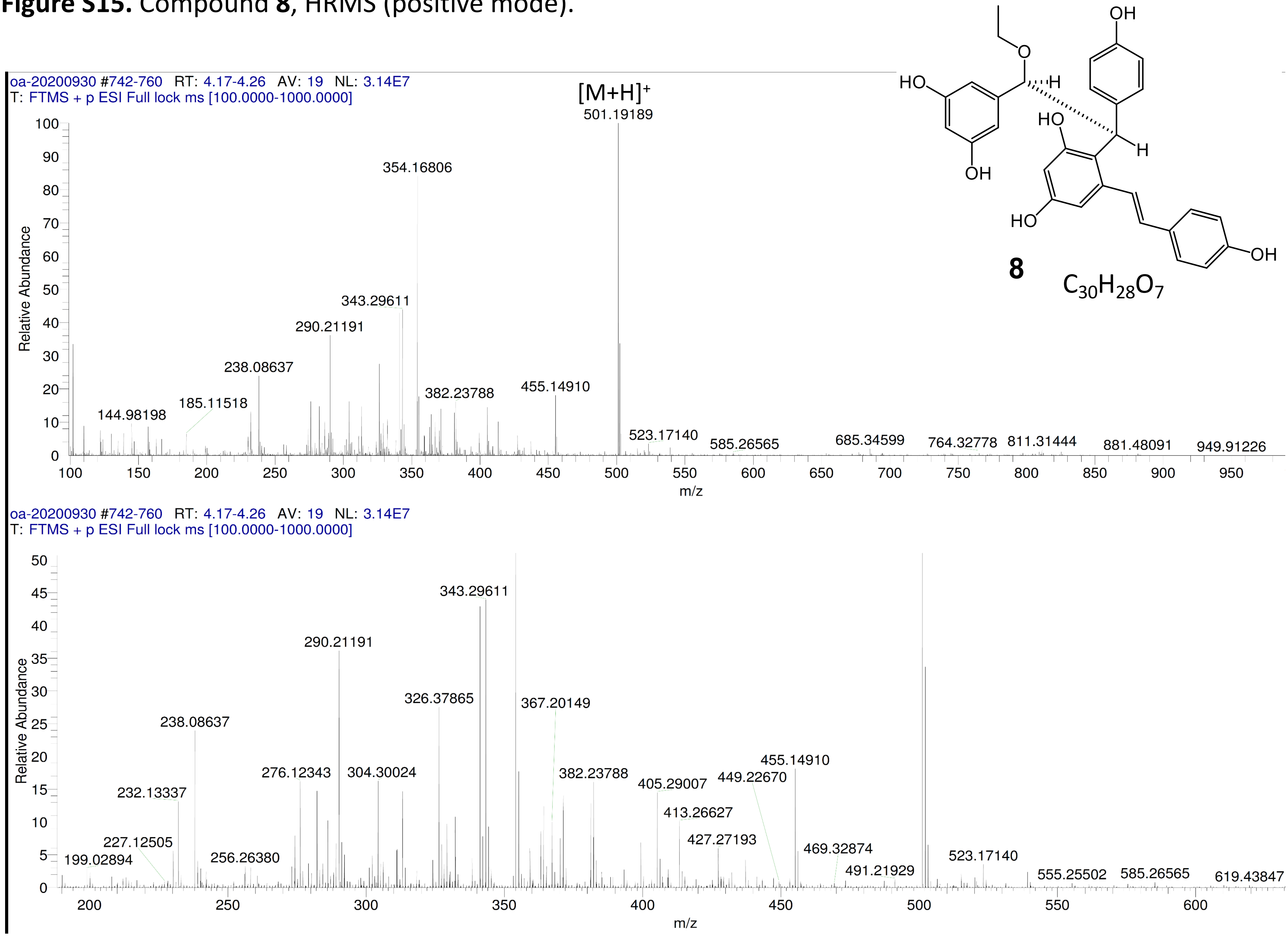

8 $C_{30}H_{28}O_7$

Figure S16. Compound **2**, ^1H NMR spectrum.

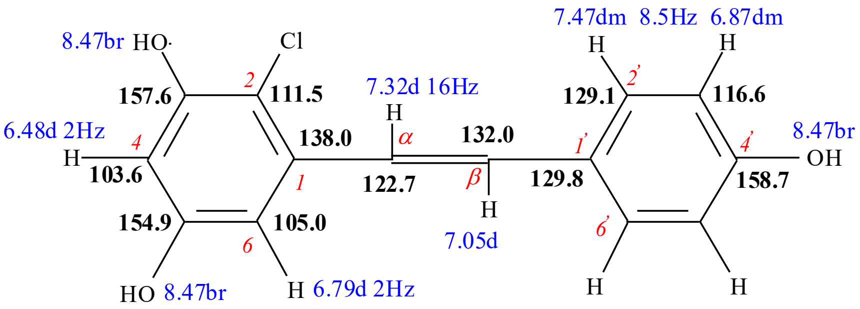

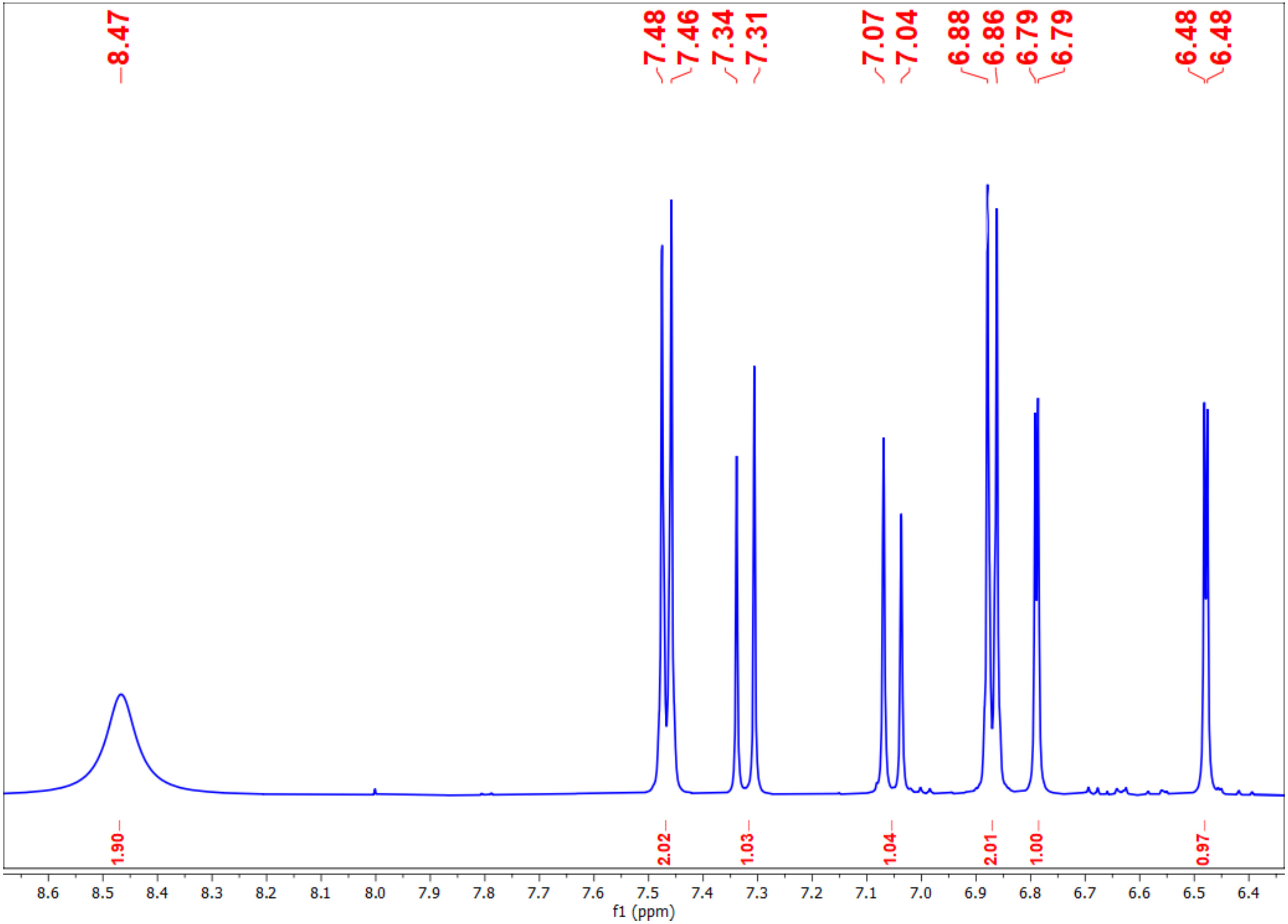

Figure S17. Compound **2**, ^{13}C, APT NMR spectrum

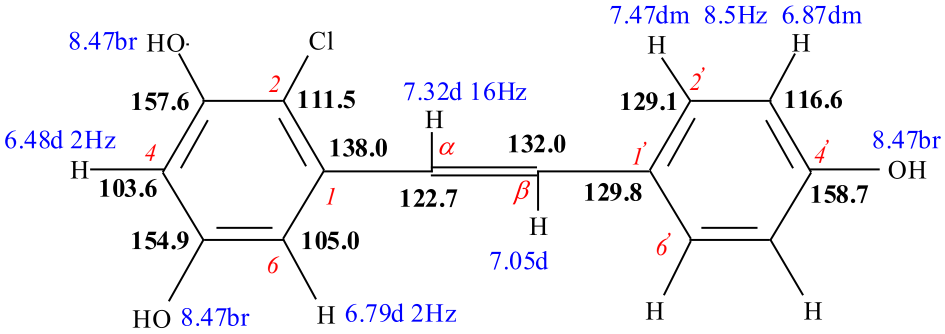

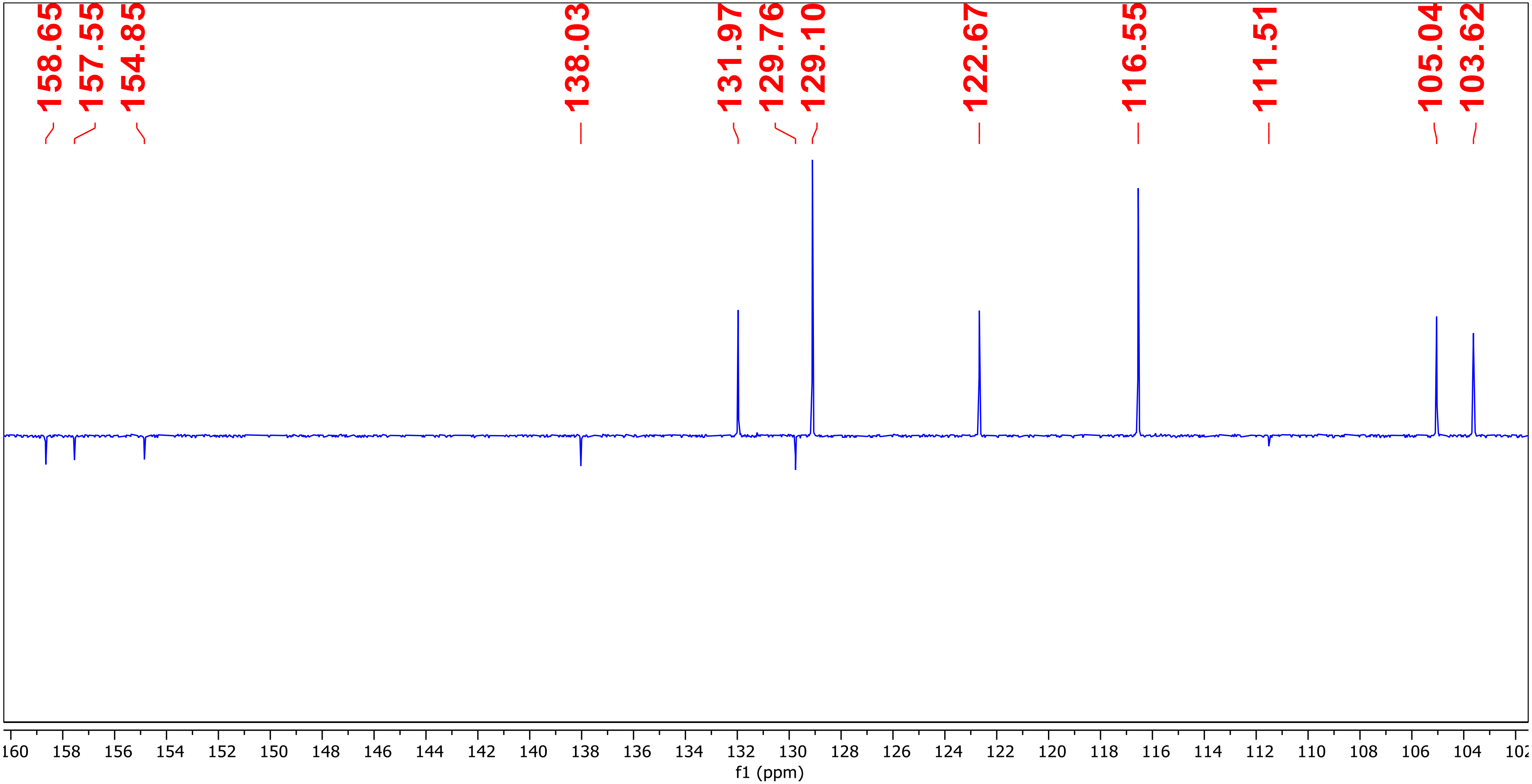

Figure S18. Compound **2**, HSQC and HMBC spectra.

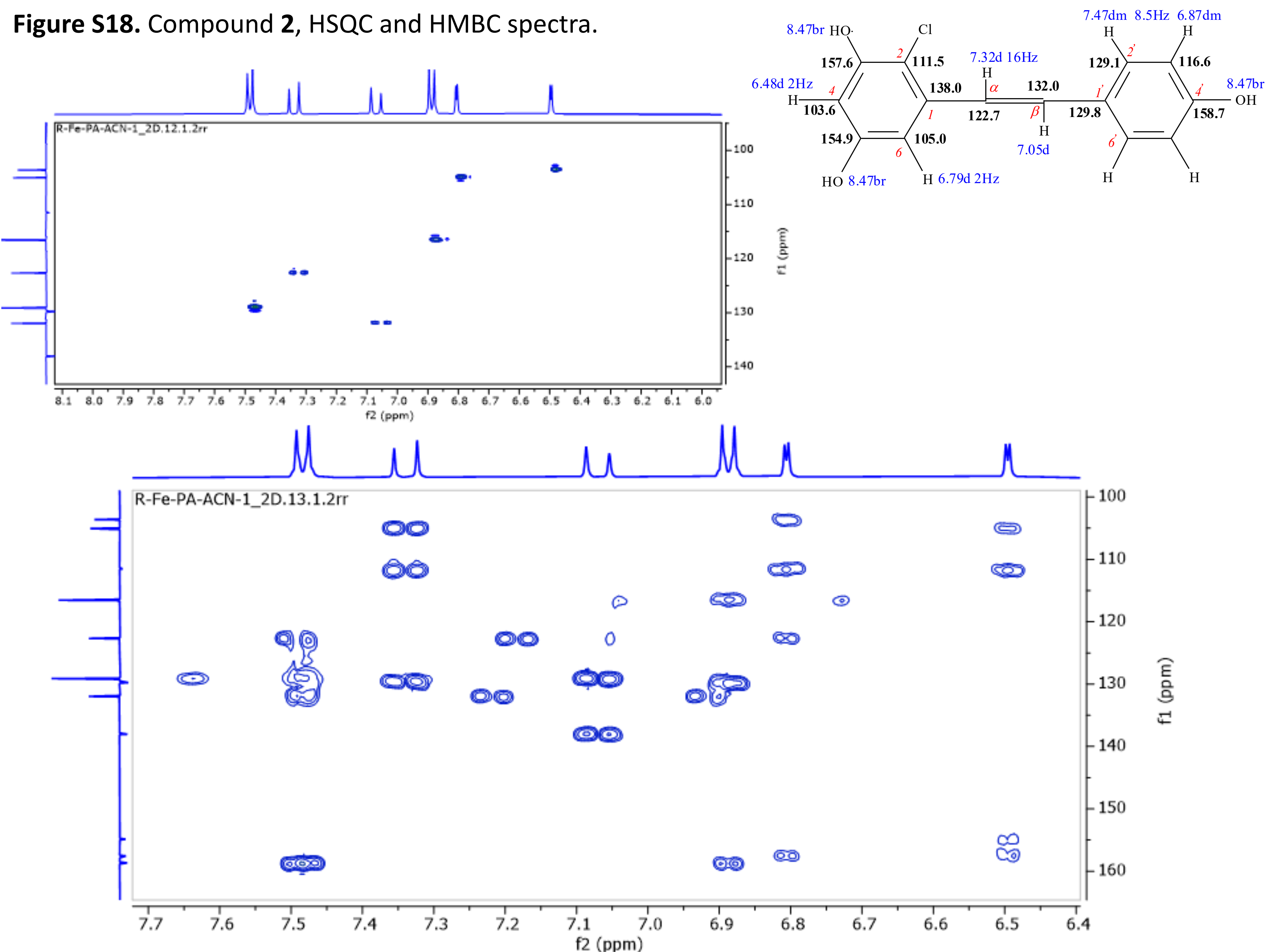

Figure S19. Compound **2**, COSY and NOESY spectra.

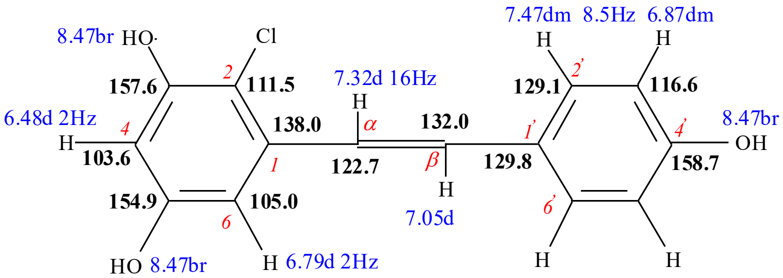

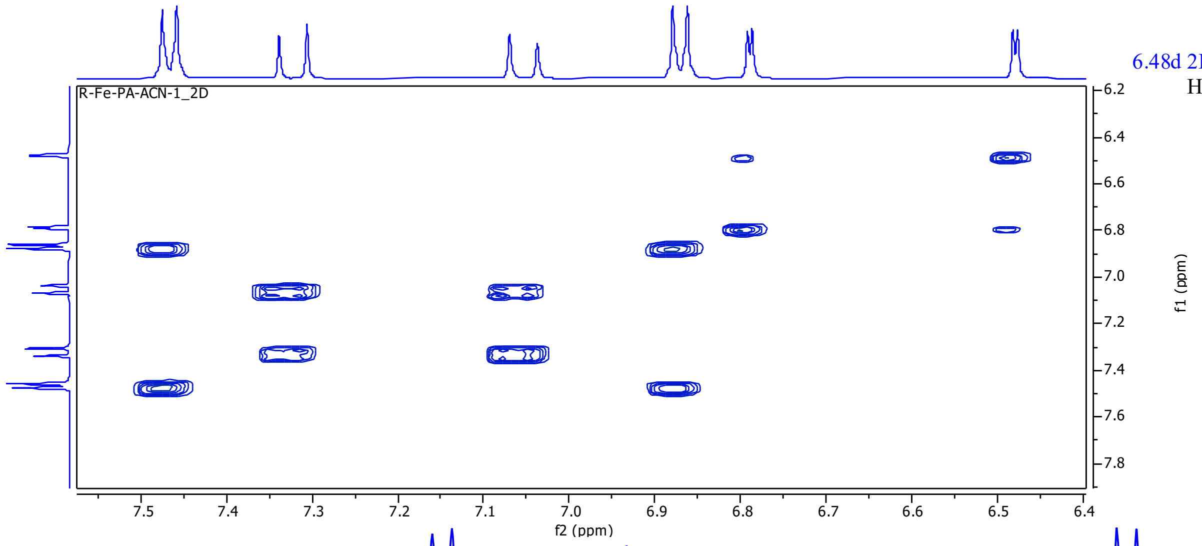

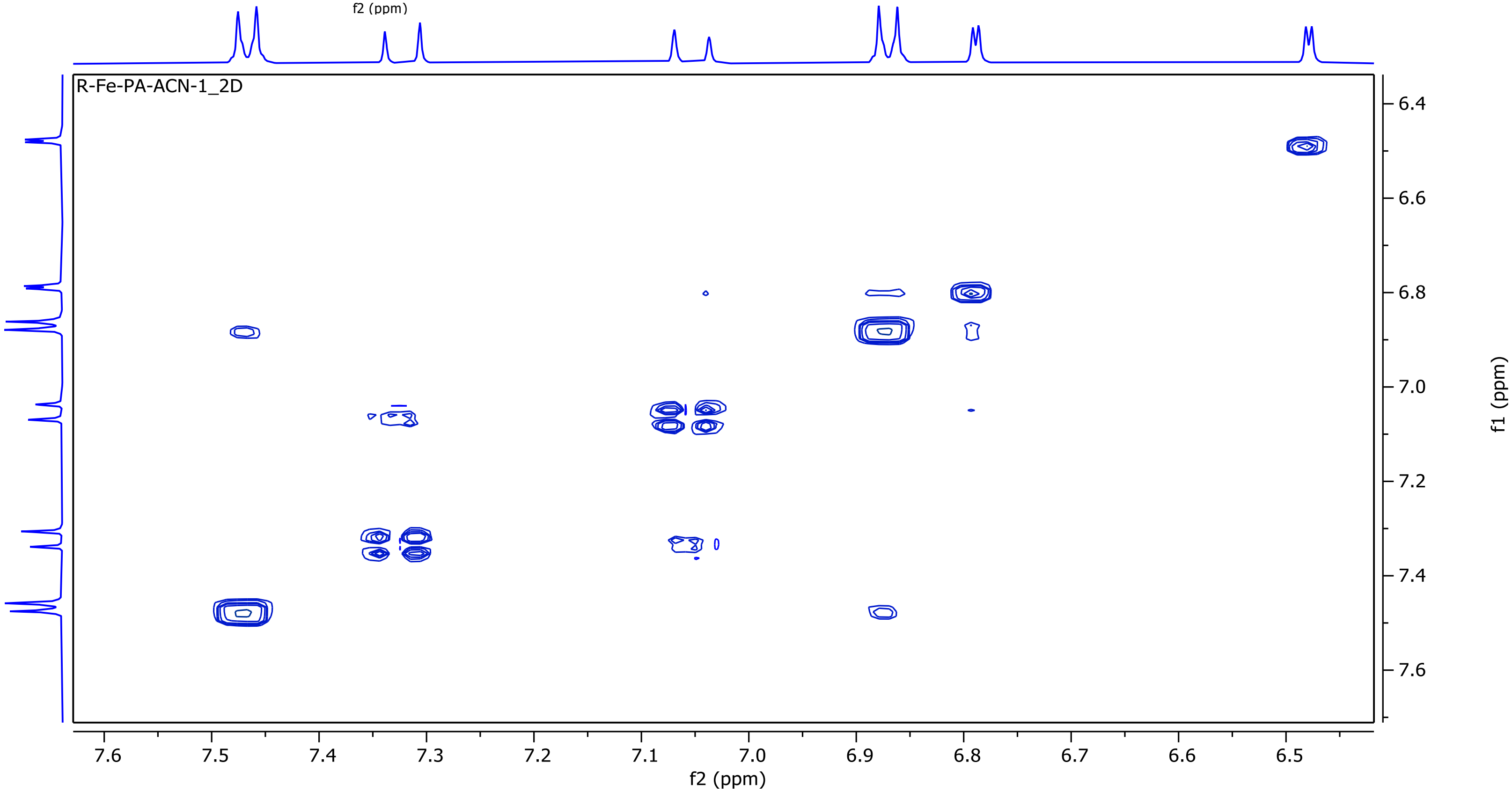

Figure S20. Compound **3**, ^{1}H NMR spectrum.

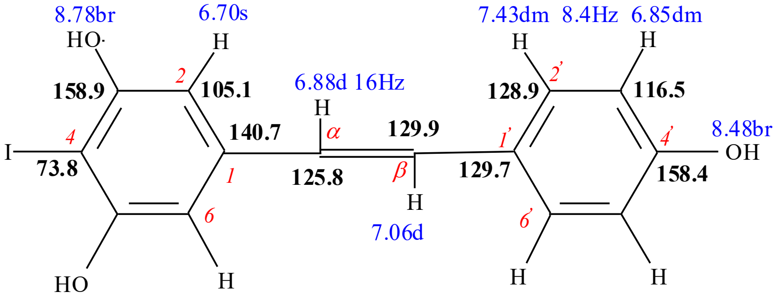

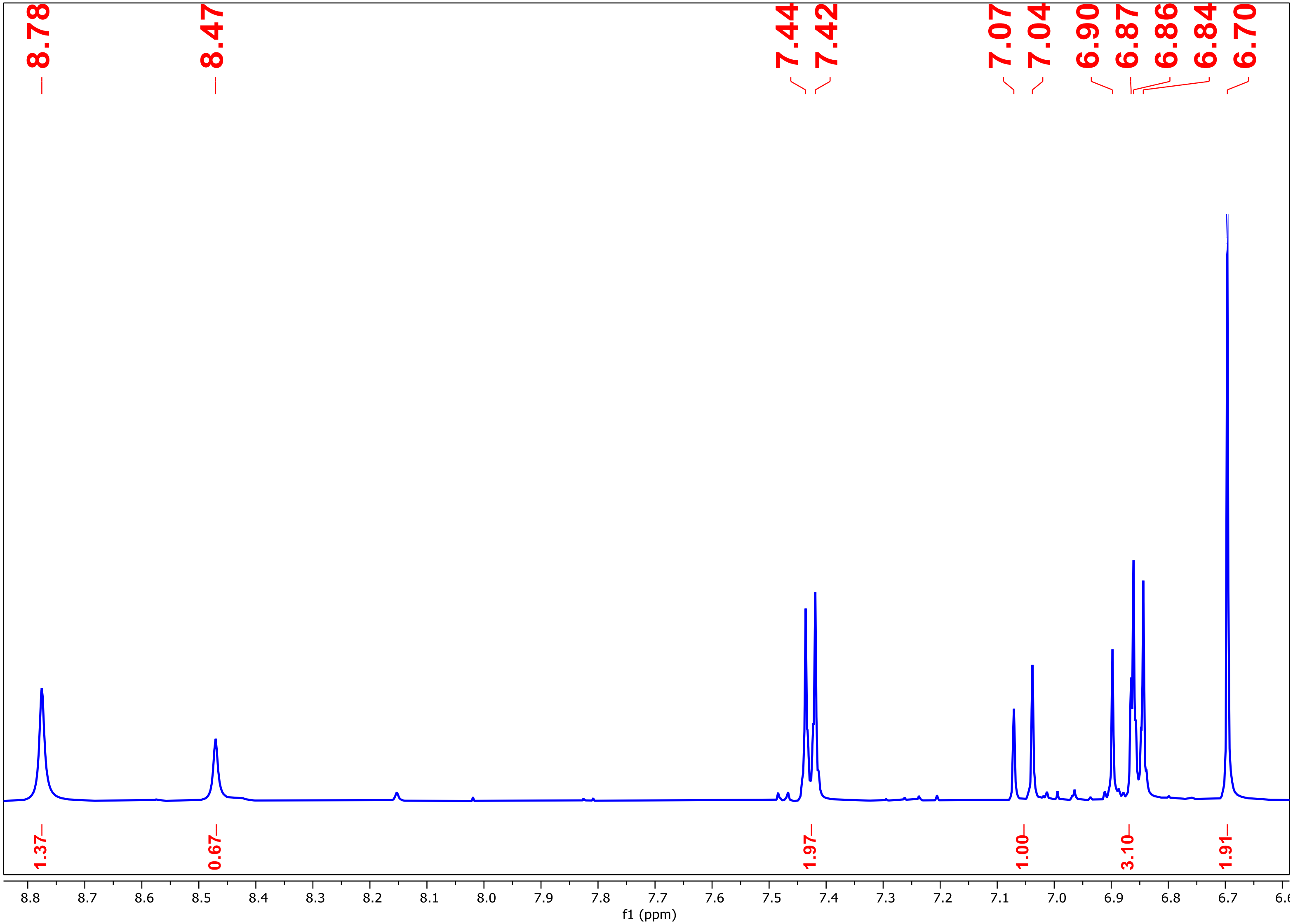

Figure S21. Compound **3**, ^{13}C, APT NMR spectrum.

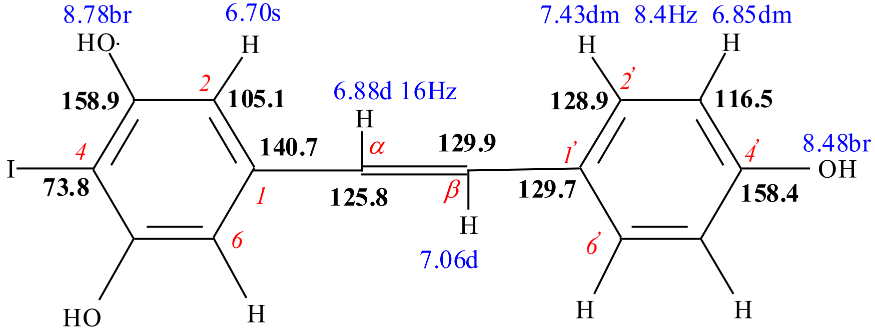

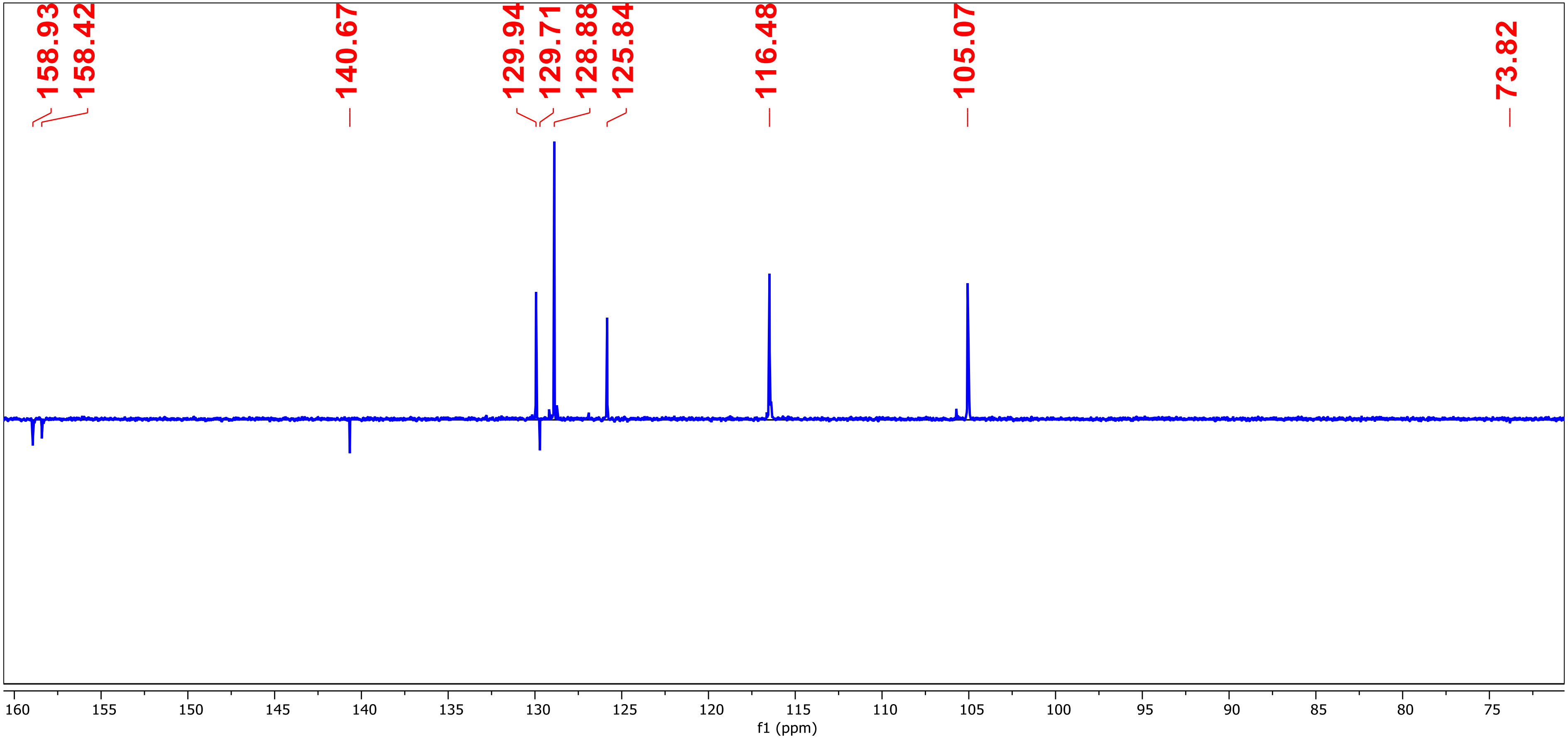

Figure S22. Compound **3**, HSQC and HMBCspectra.

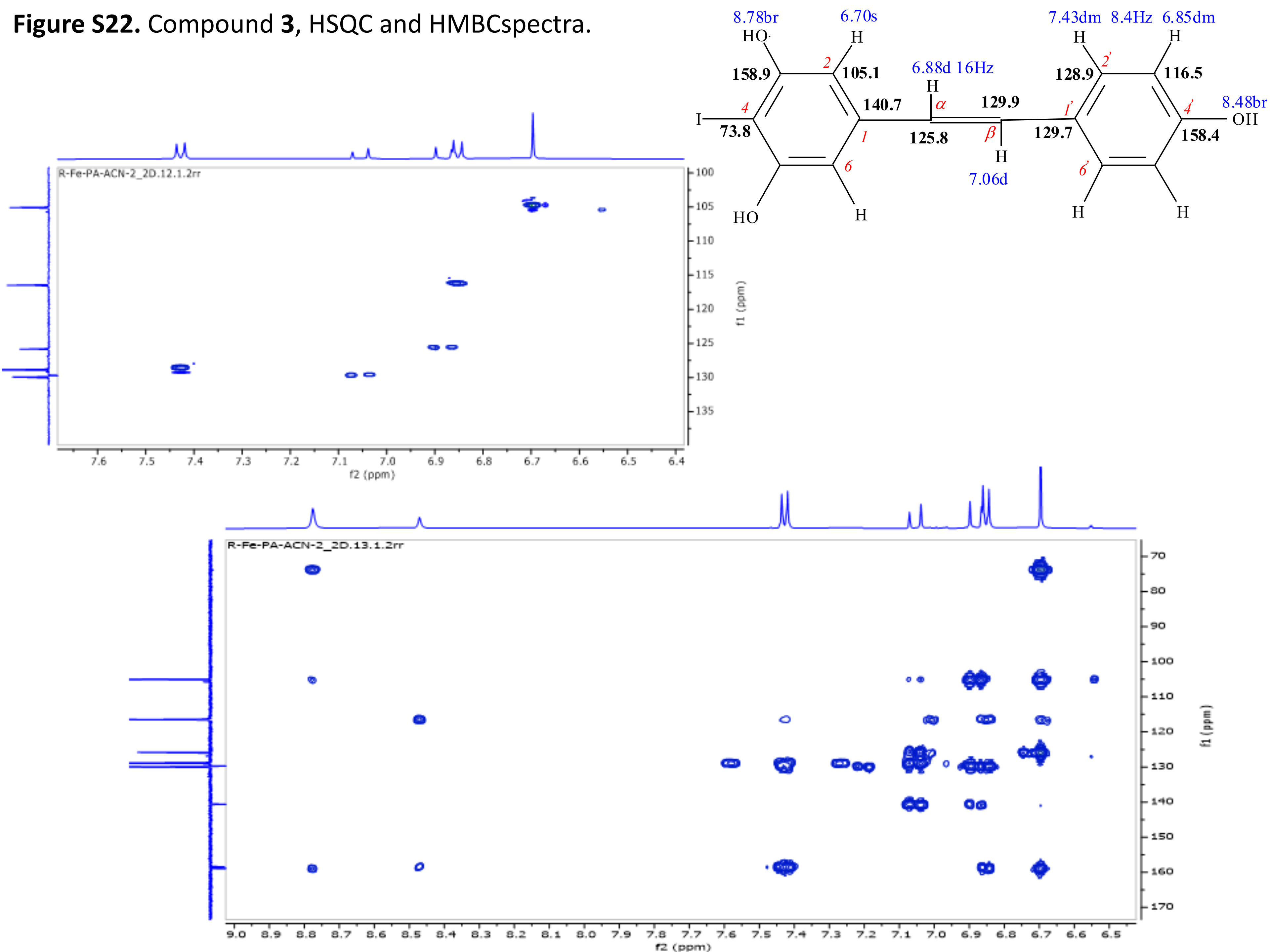

Figure S23. Compound **3**, COSY spectrum.

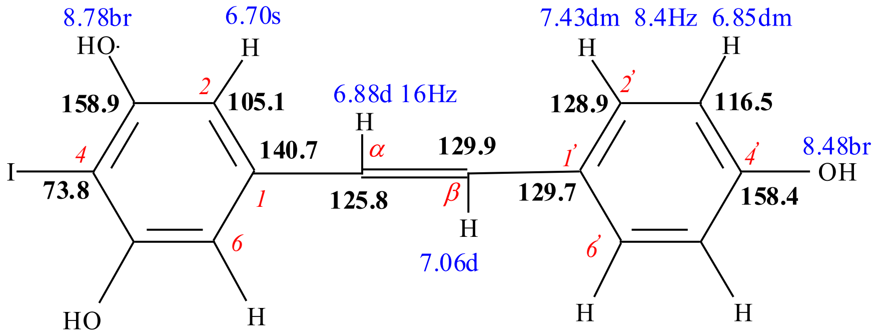

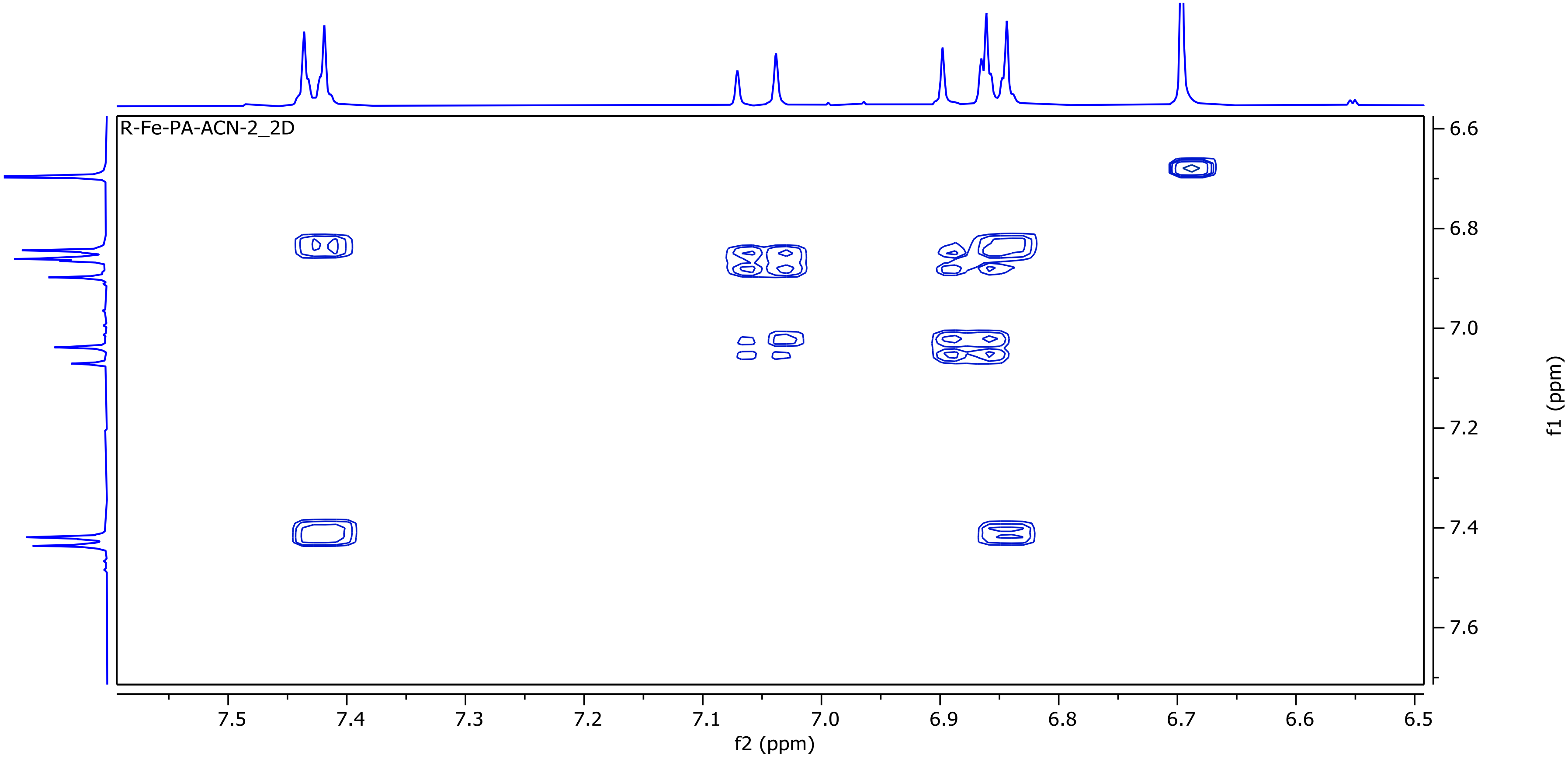

Figure S24. Compound **4**, ^{1}H NMR spectrum.

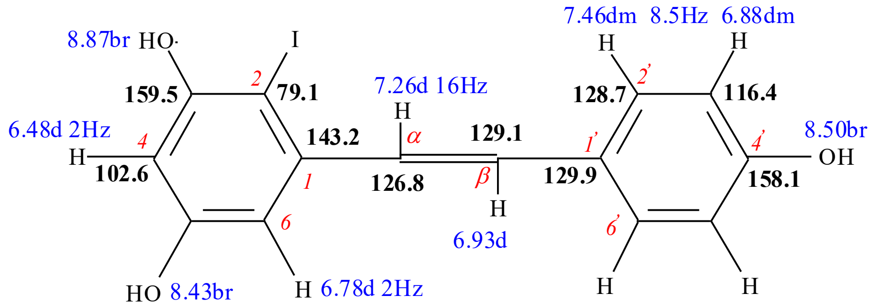

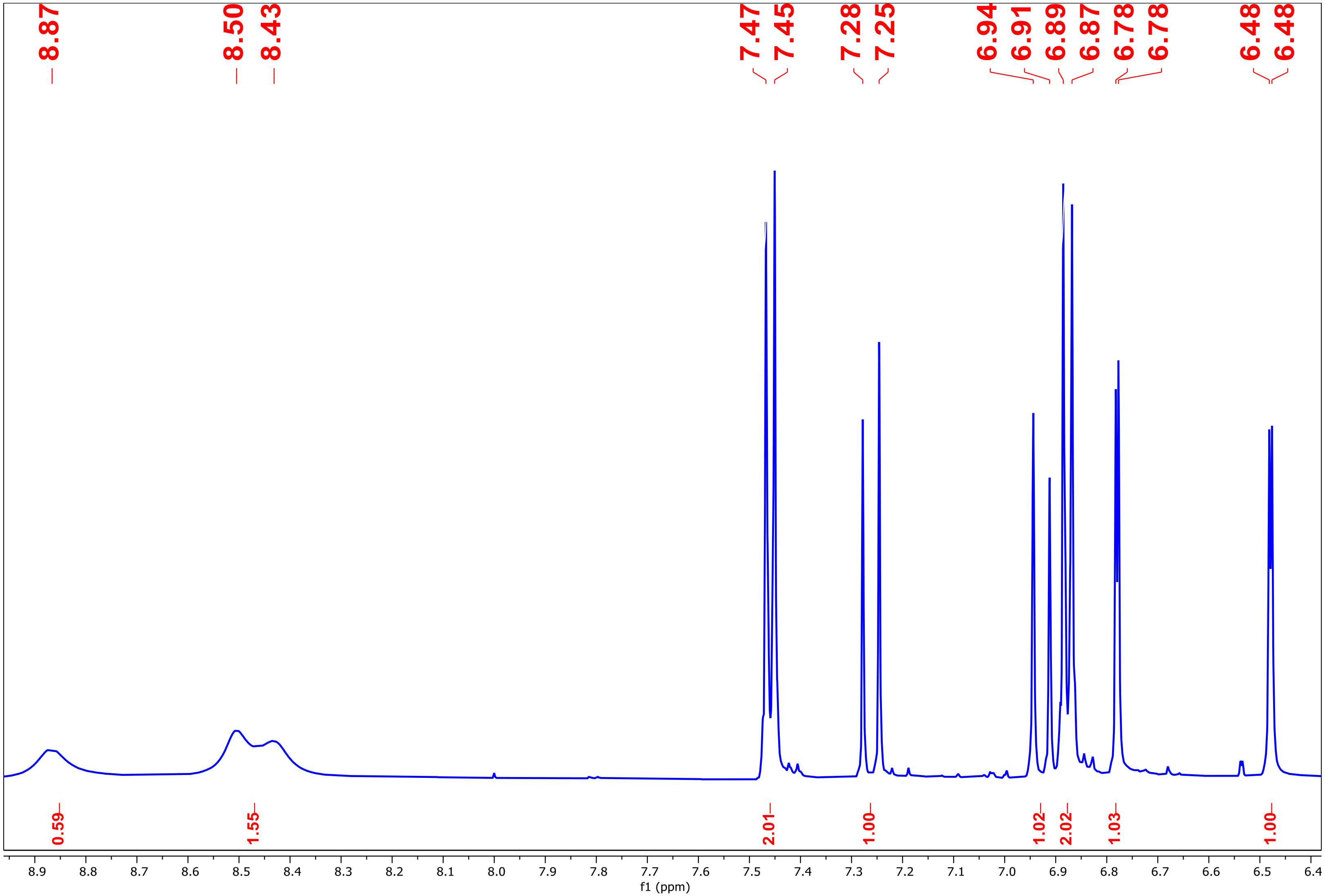

Figure S25. Compound **4**, ^{13}C, APT NMR spectrum.

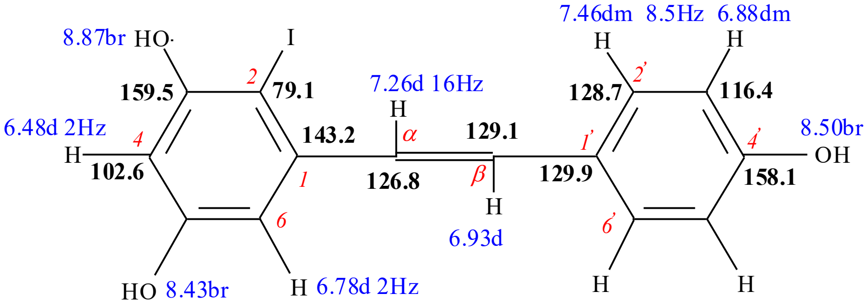

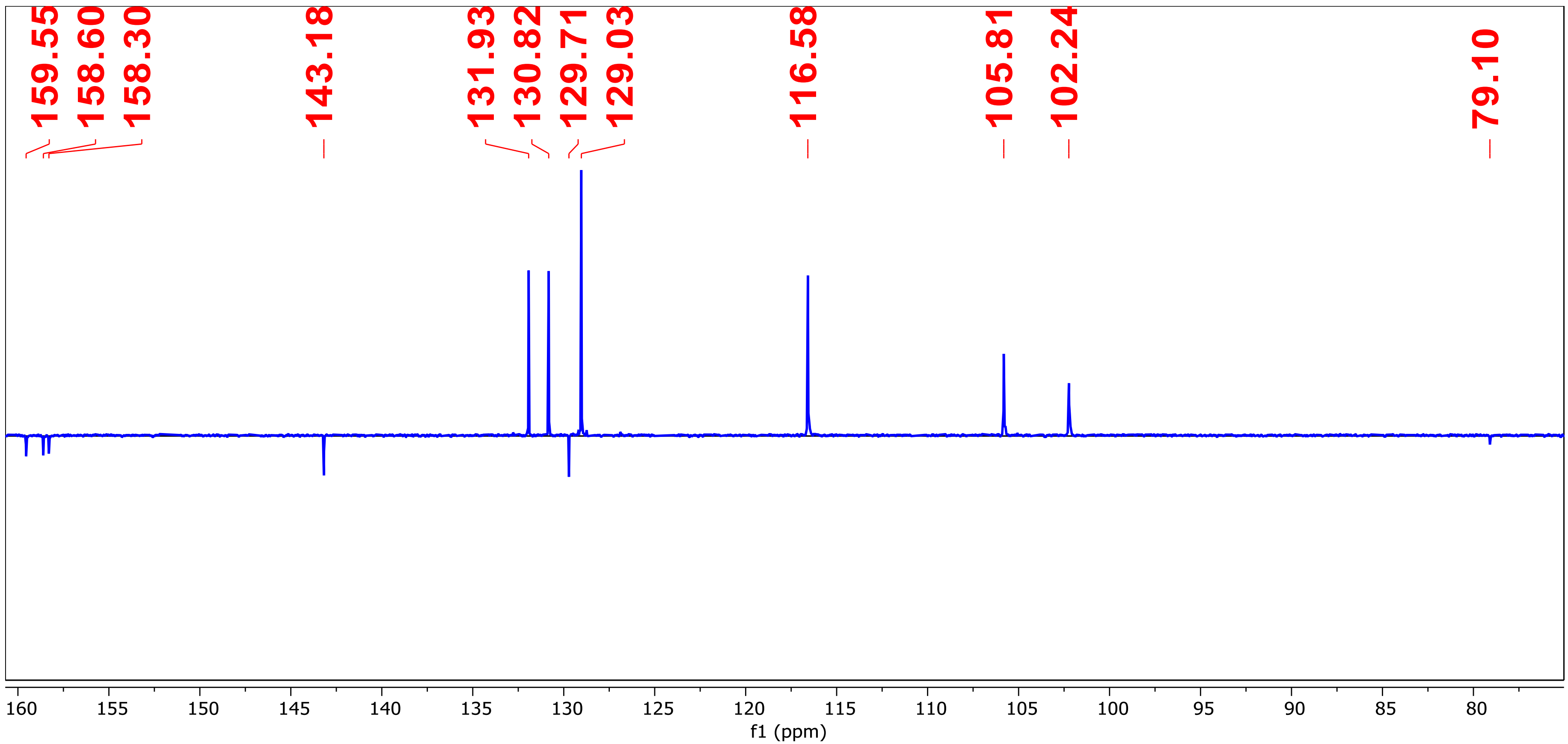

Figure S26. Compound **4**, HSQC and HMBC spectra.

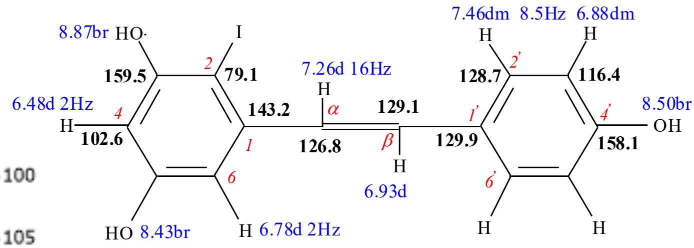

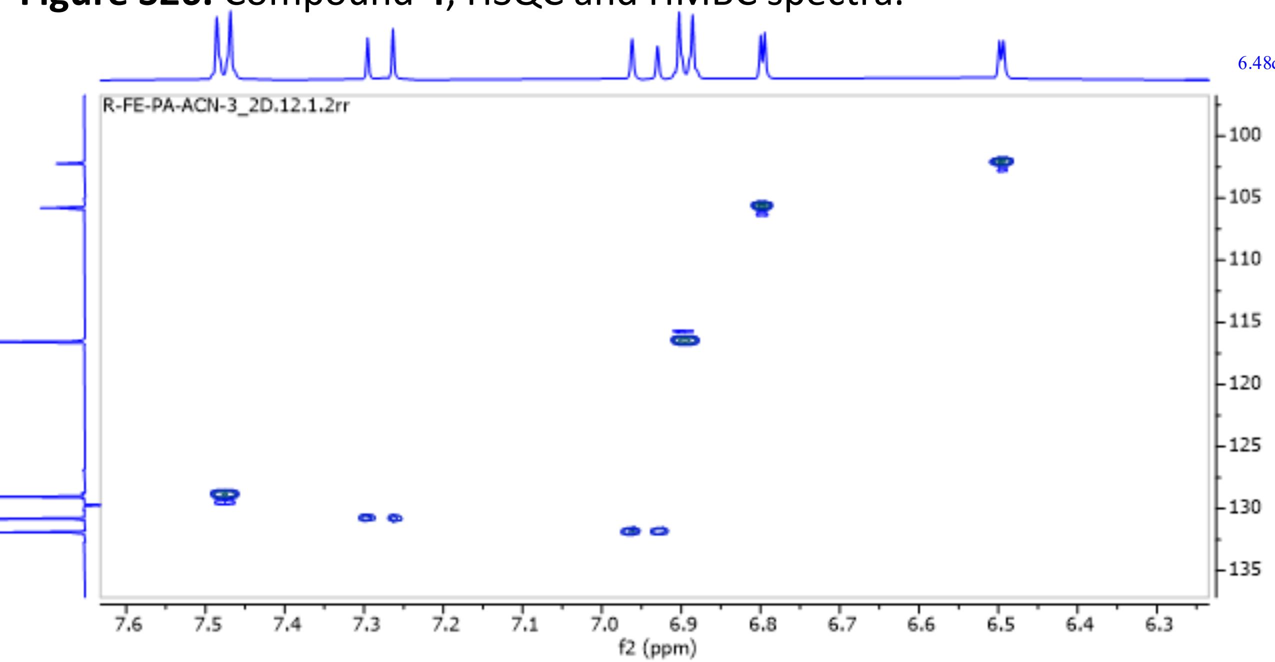

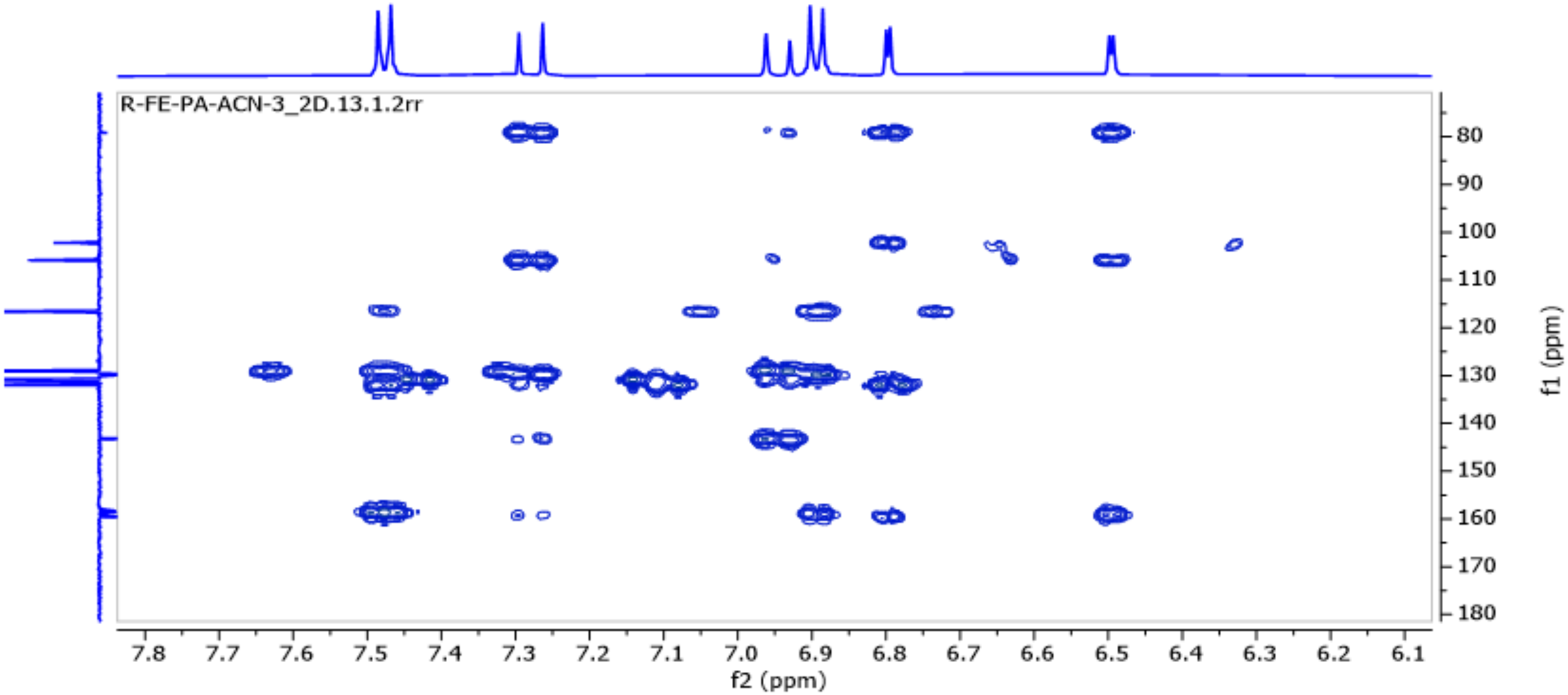

Figure S27. Compound **4**, COSY spectrum.

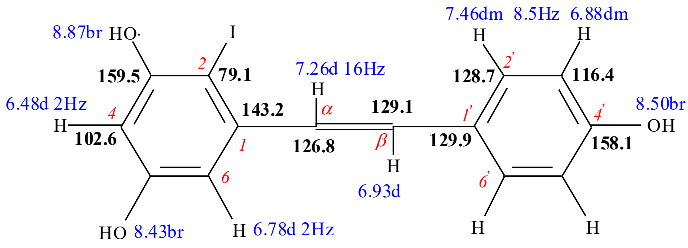

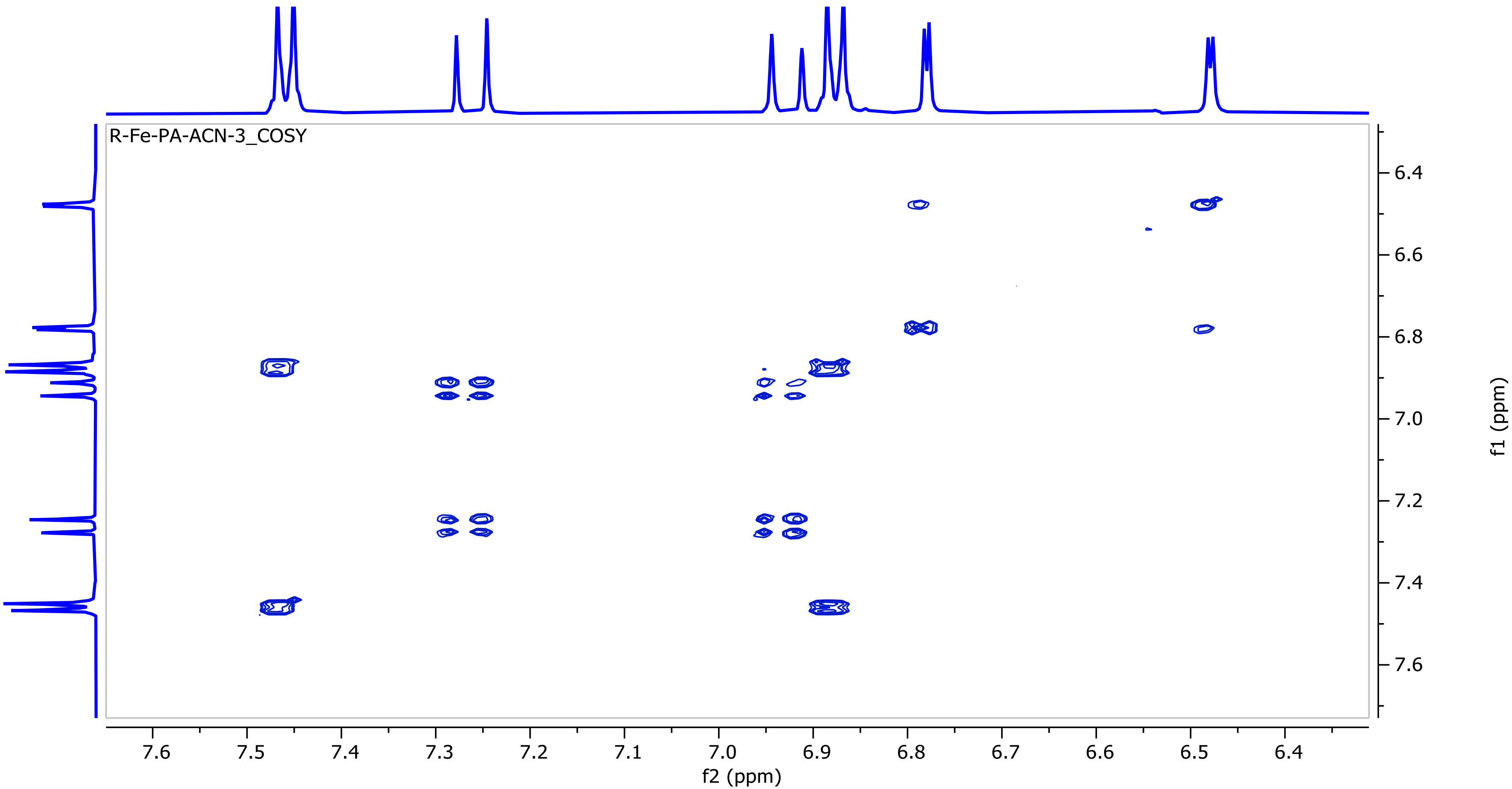

Figure S28. Compound **5**, ^{1}H NMR spectrum, and selROE on δ4.47 and δ5.45 ppm.

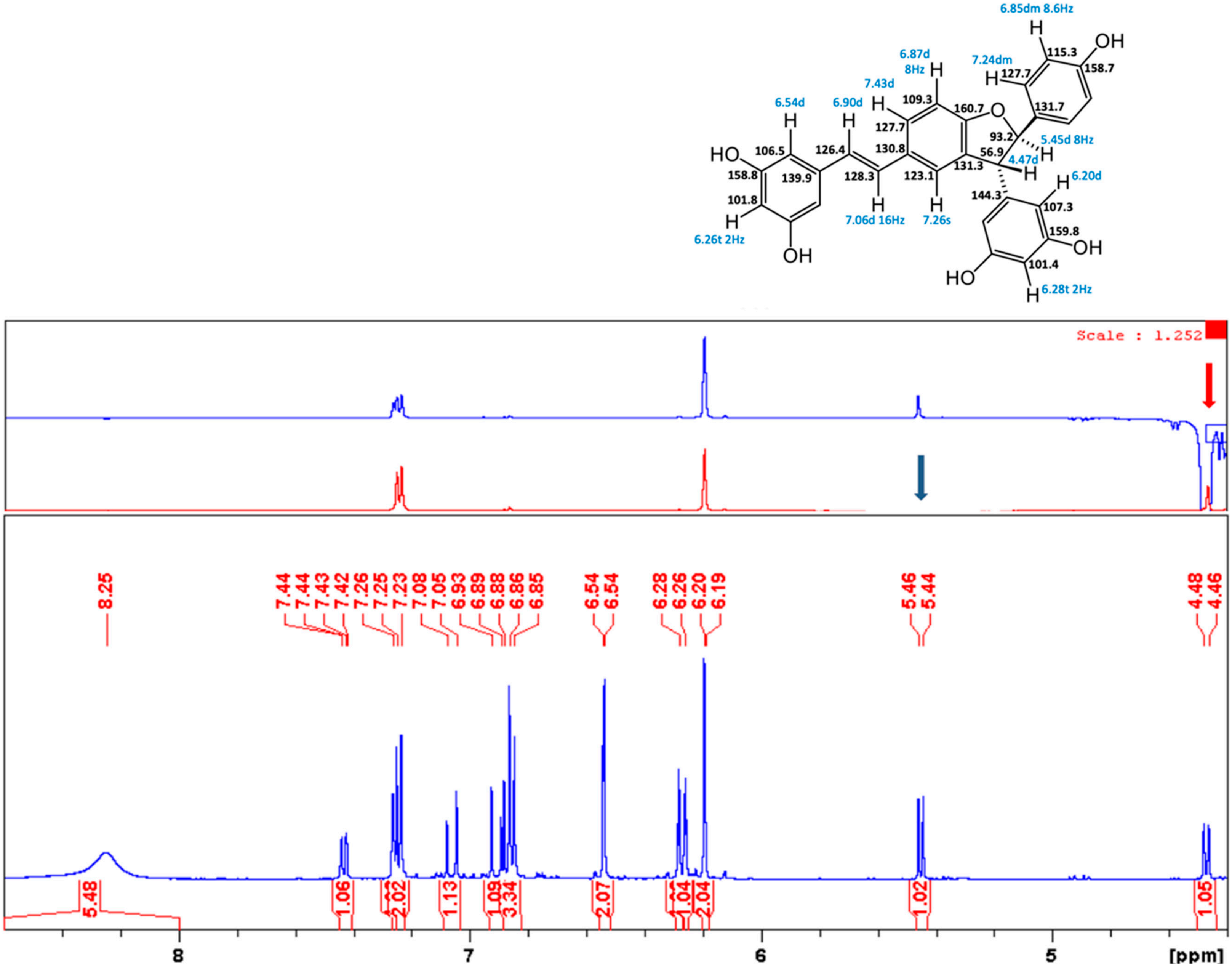

Figure S29. Compound **5**, ^{13}C, APT NMR spectrum.

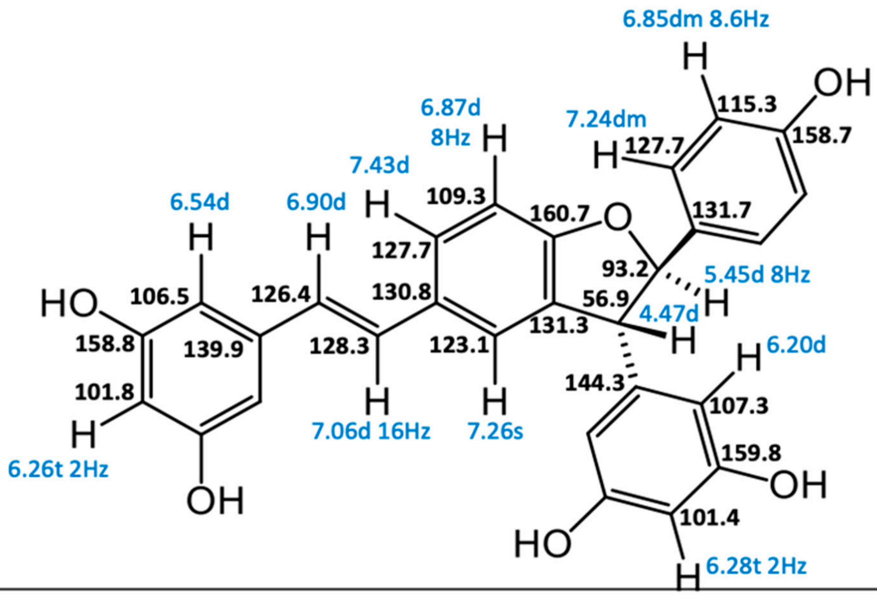

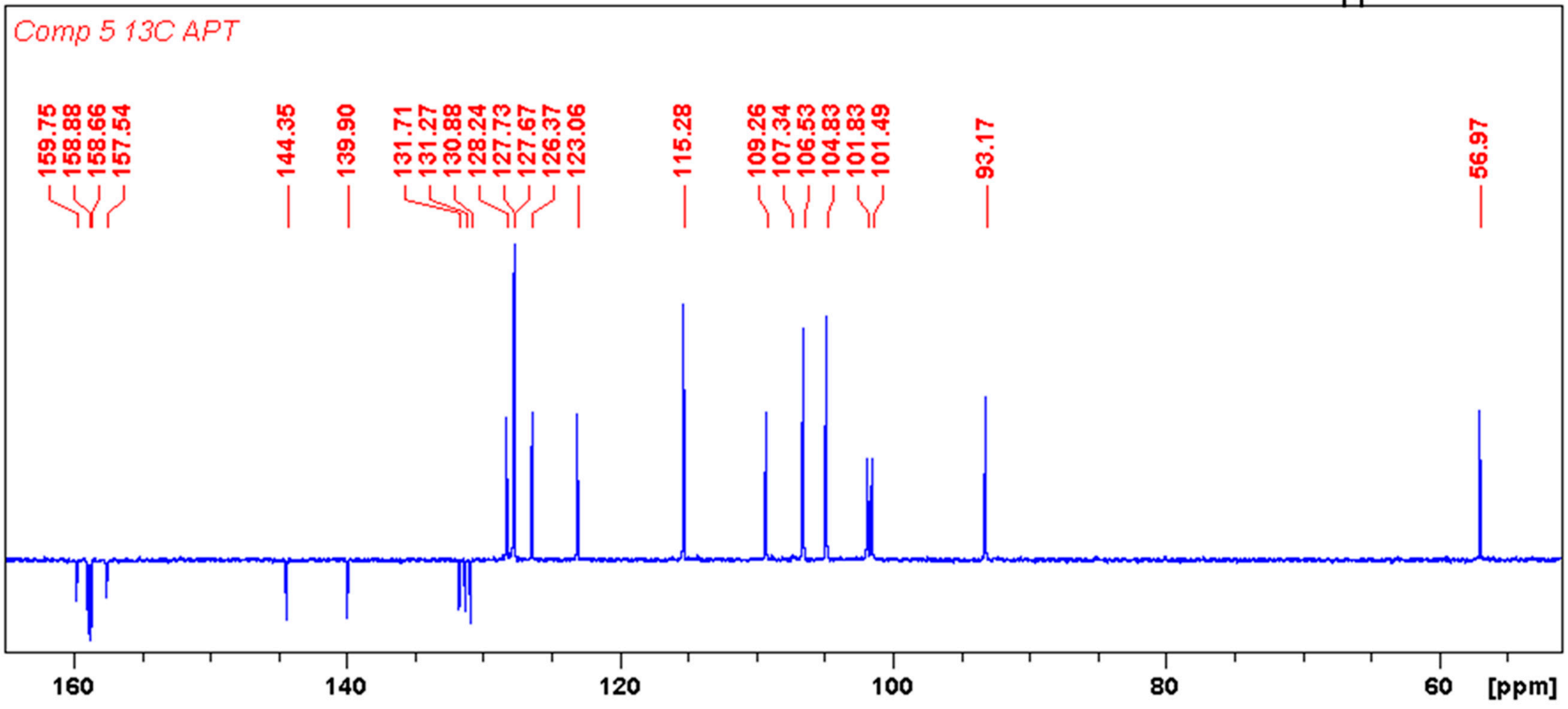

Figure S30. Compound **5**, HSQC spectrum and HSQC section.

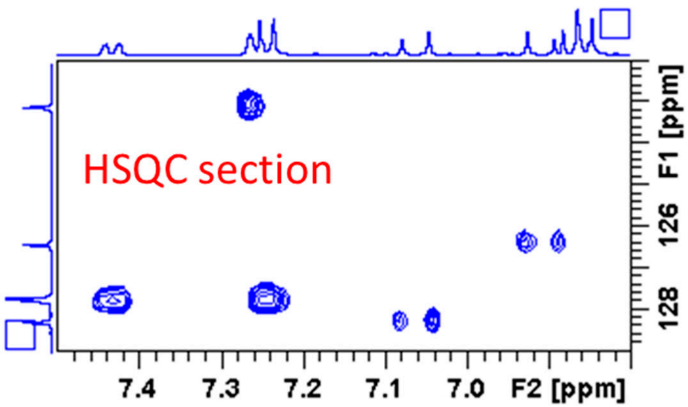

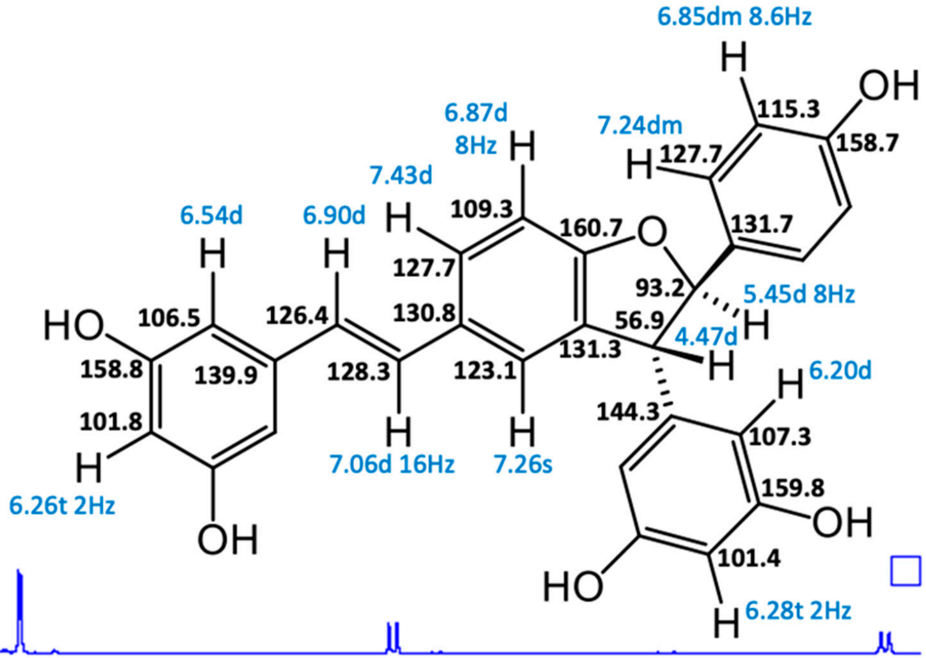

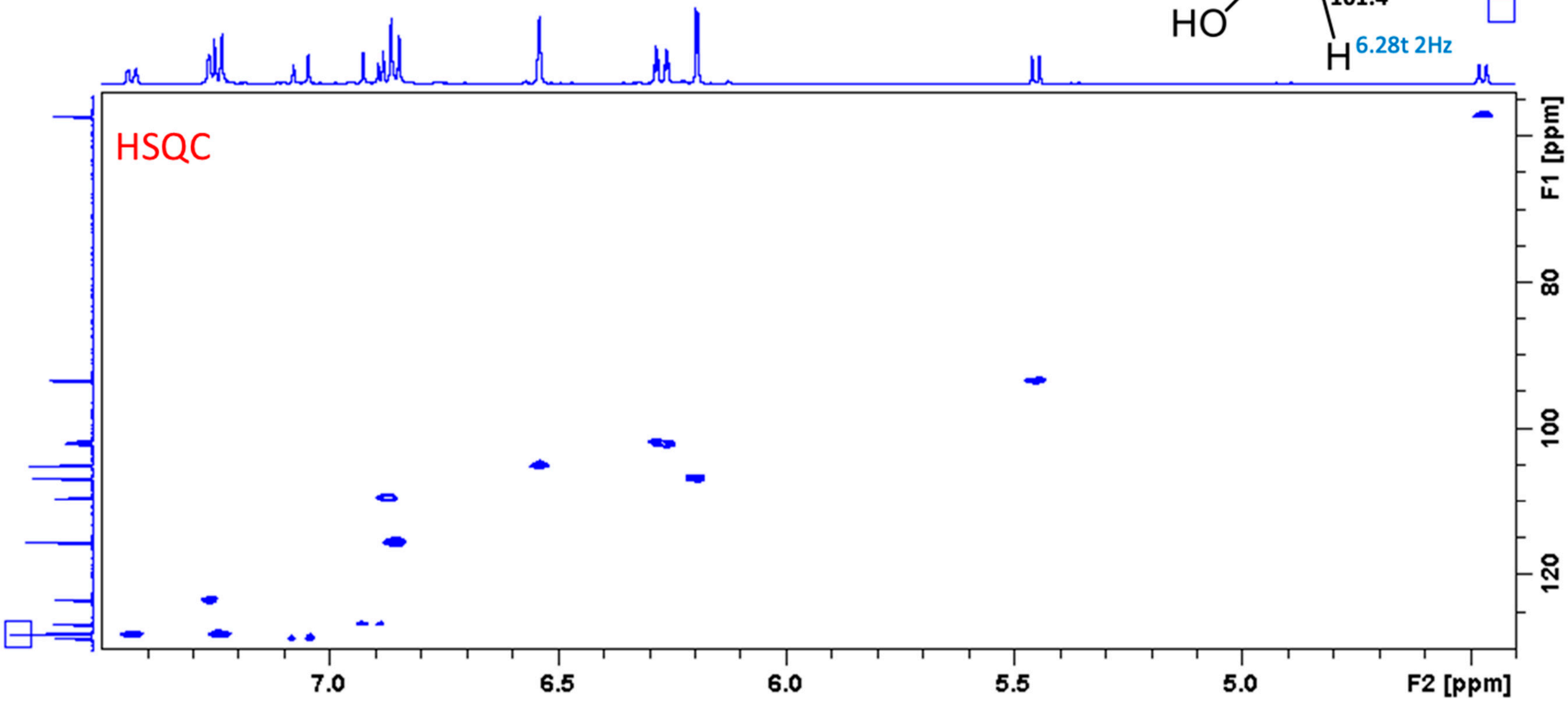

Figure S31. Compound **5**, COSY spectrum.

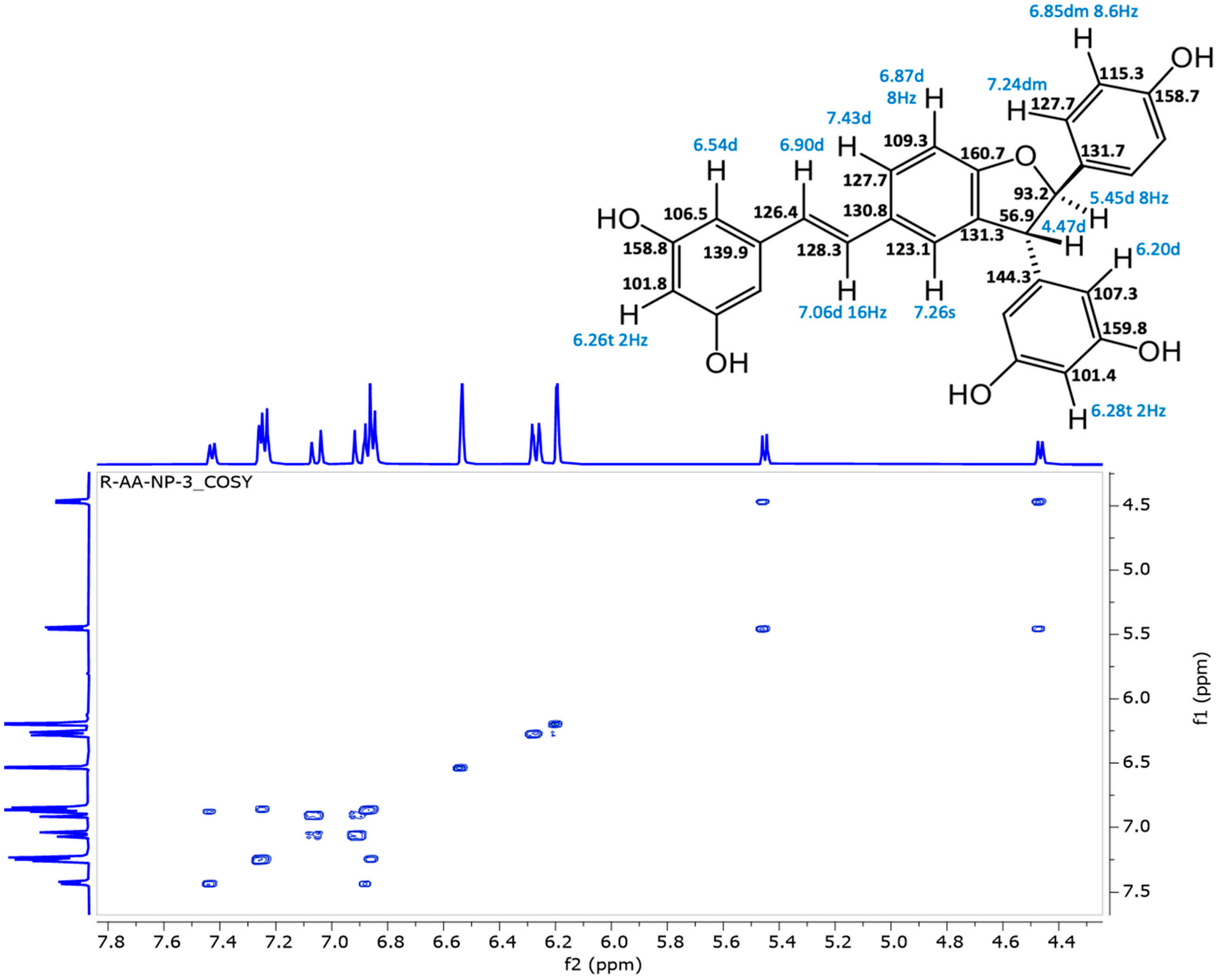

Figure S32. Compound **5**, HMBC spectrum.

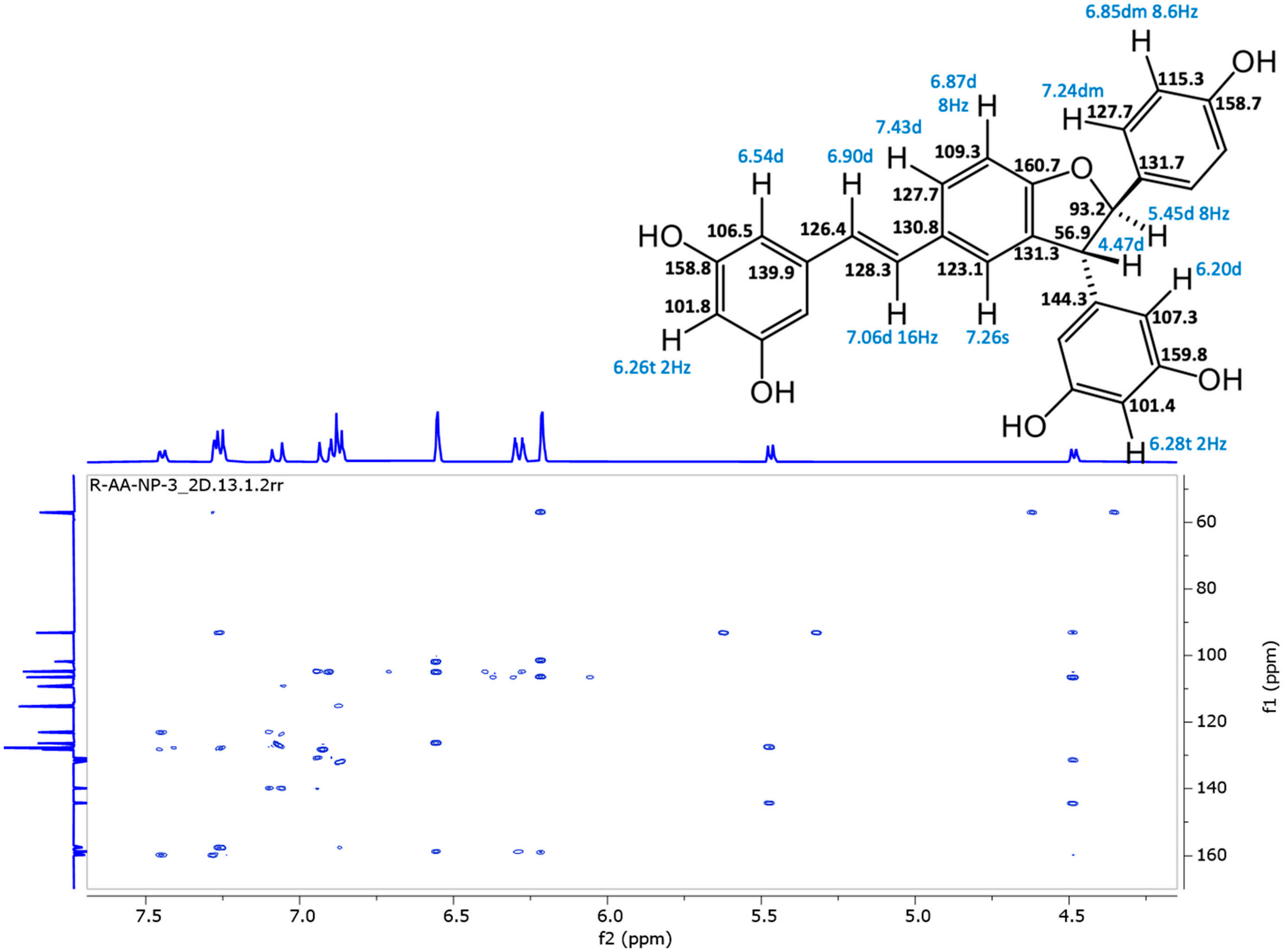

Figure S33. Compound **6**, ^{1}H, selNOE on δ4.48 and δ5.42 ppm.

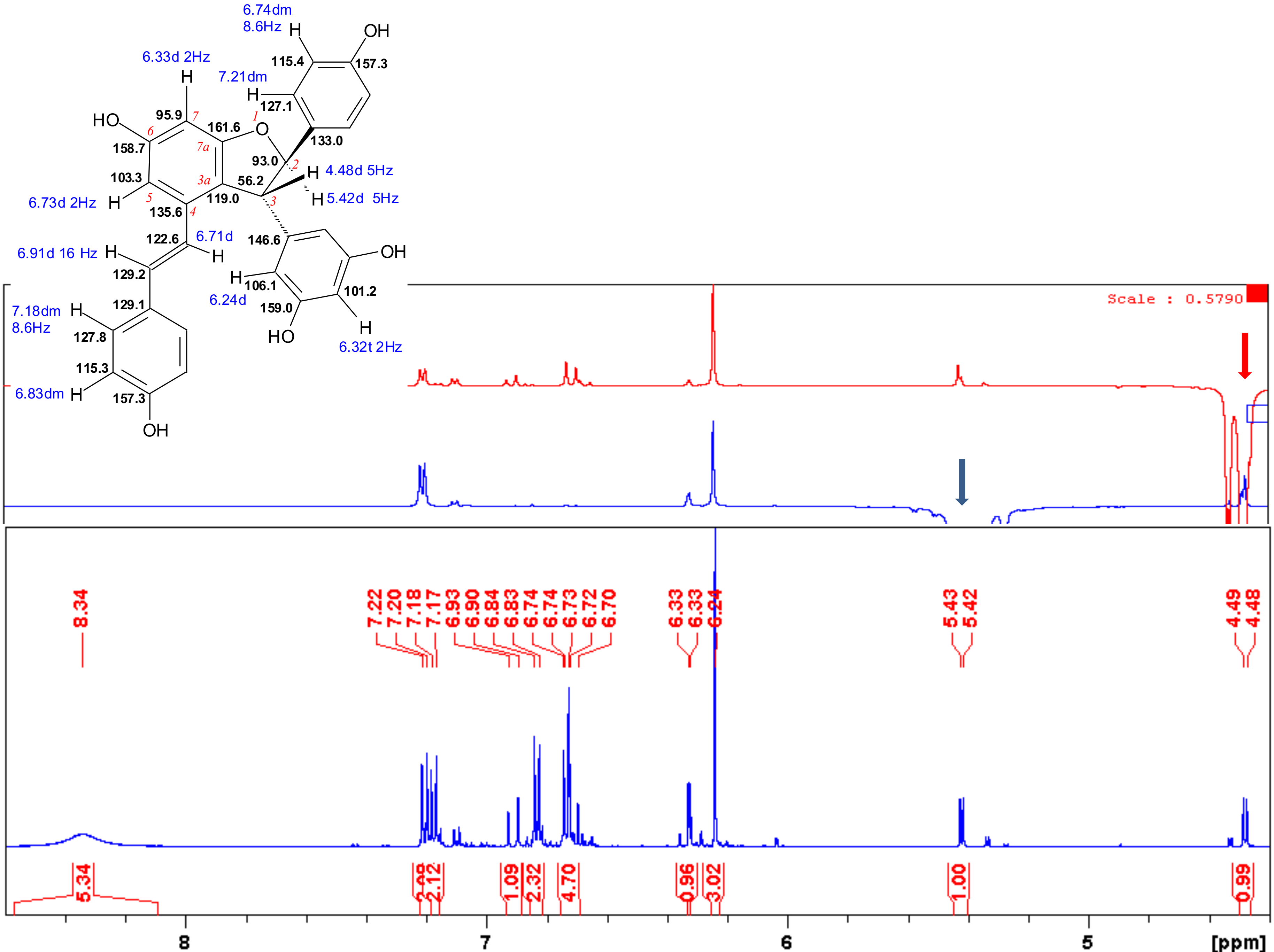

Figure S34. Compound **6**, ^{13}C, APT NMR spectrum.

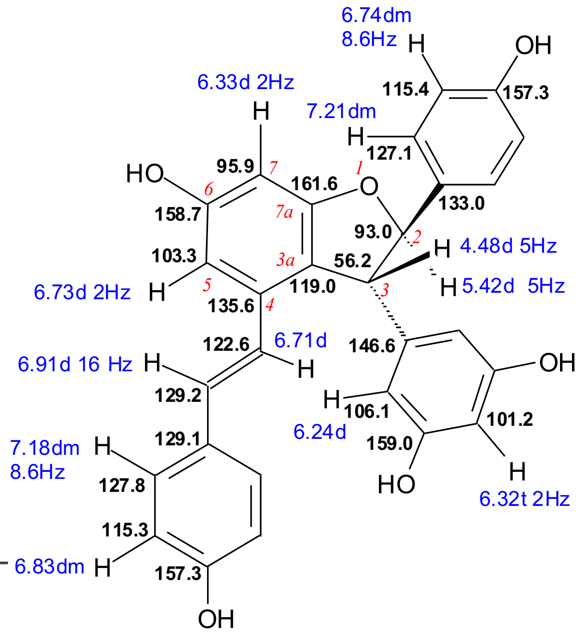

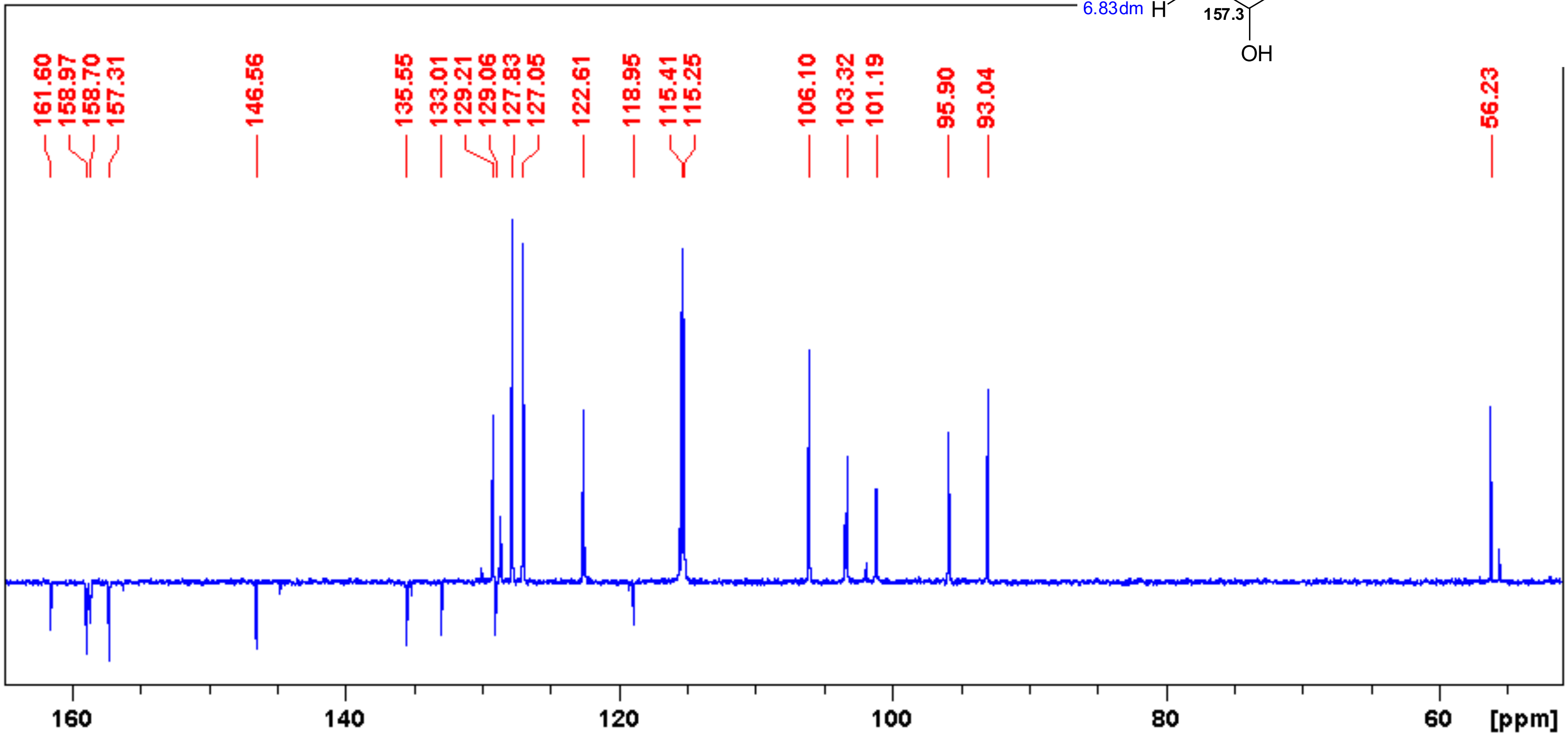

Figure S35. Compound **6**, HSQC spectrum.

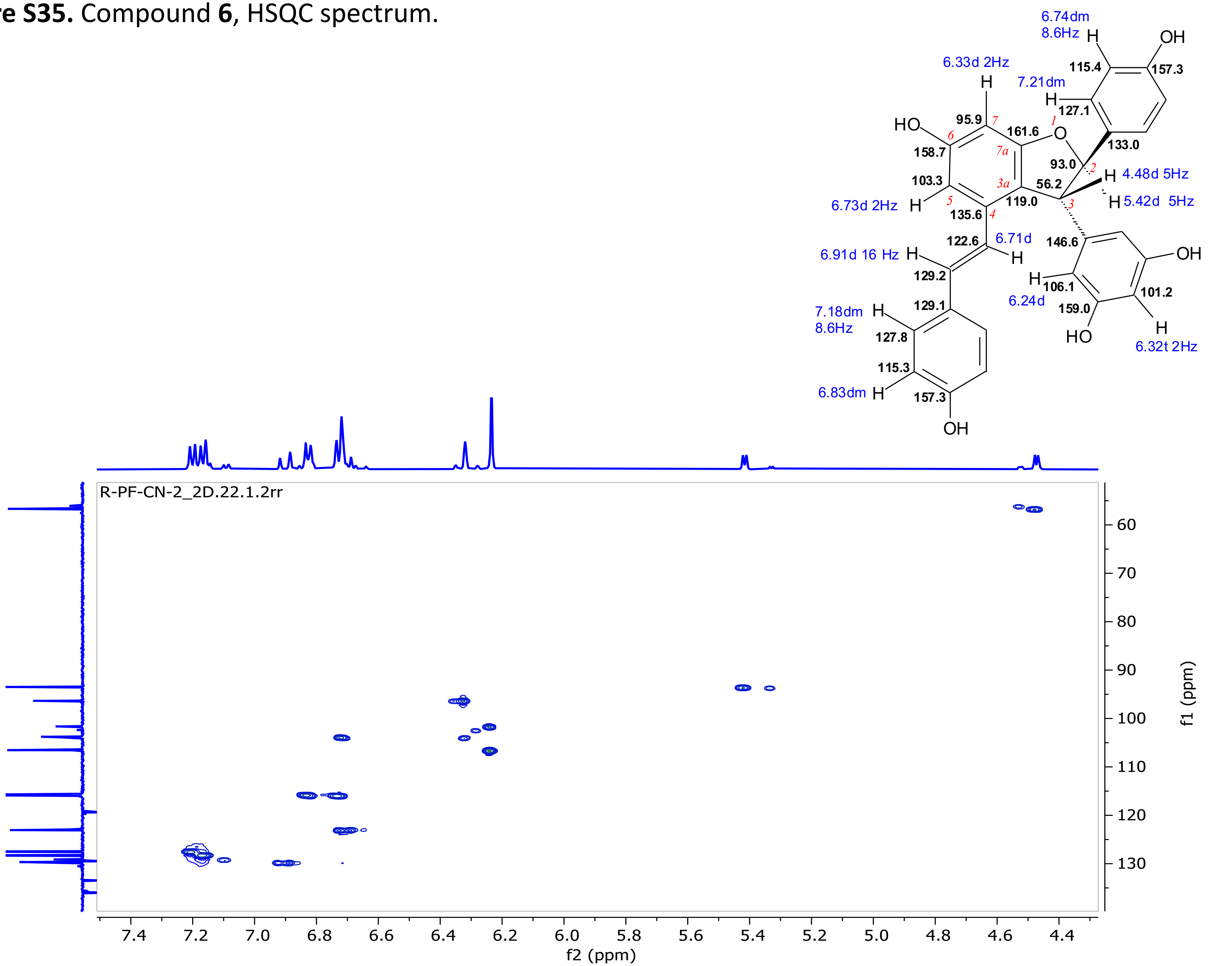

Figure S36. Compound **6**, HMBC spectrum.

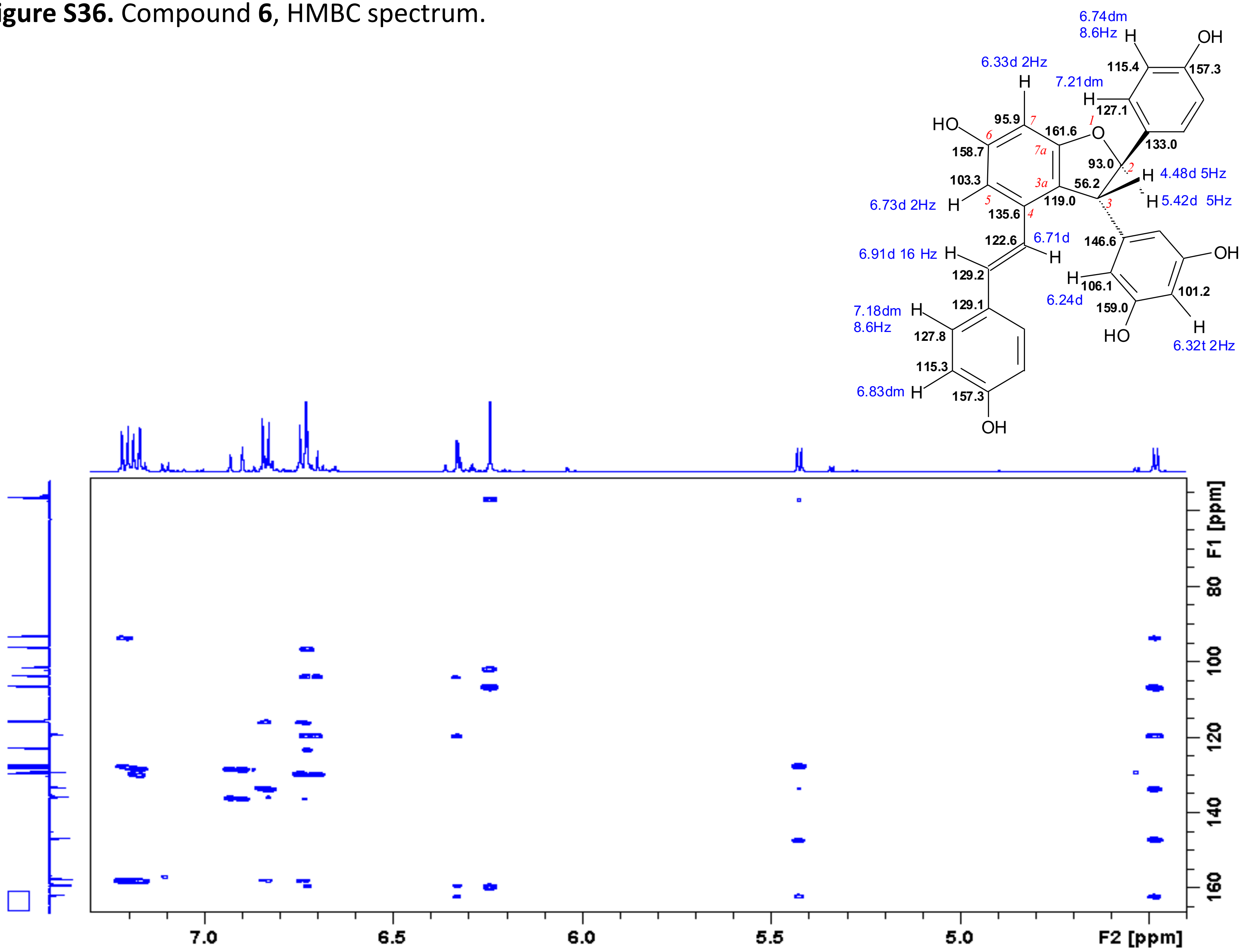

Figure S37. Compound **6**, COSY and NOESY spectra.

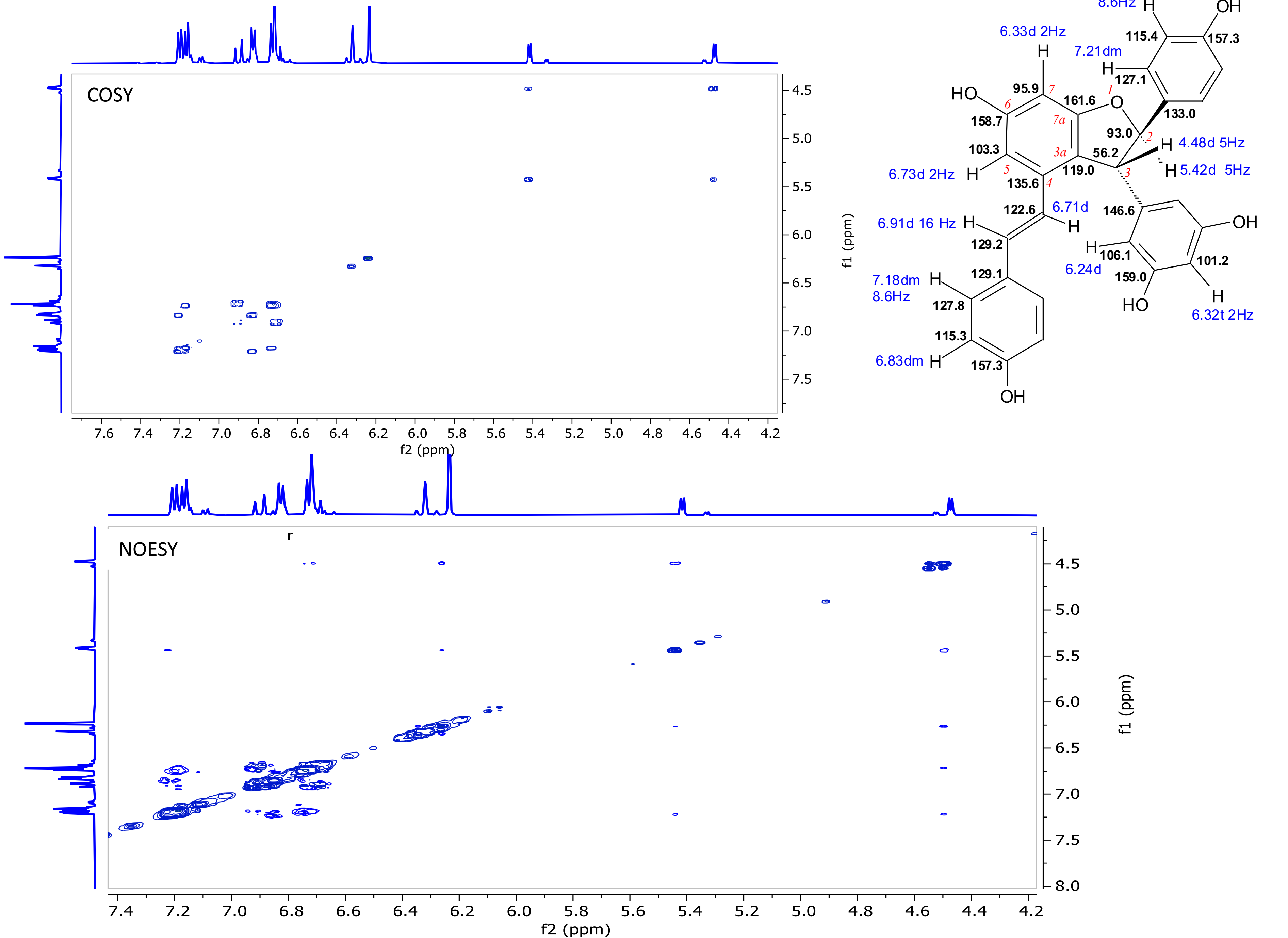

Figure S38. Compound **7**, ^{1}H NMR spectrum.

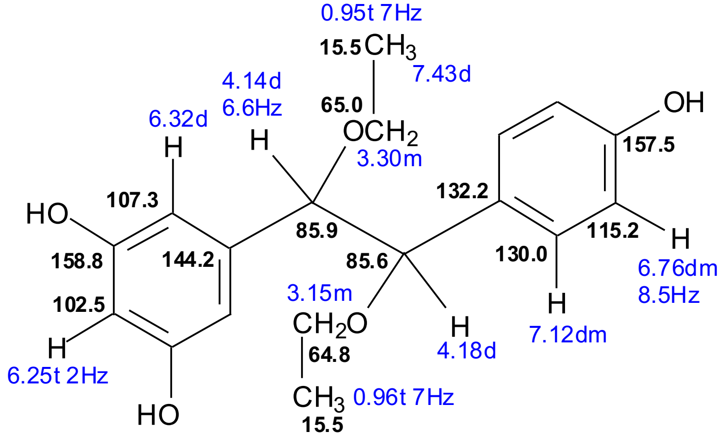

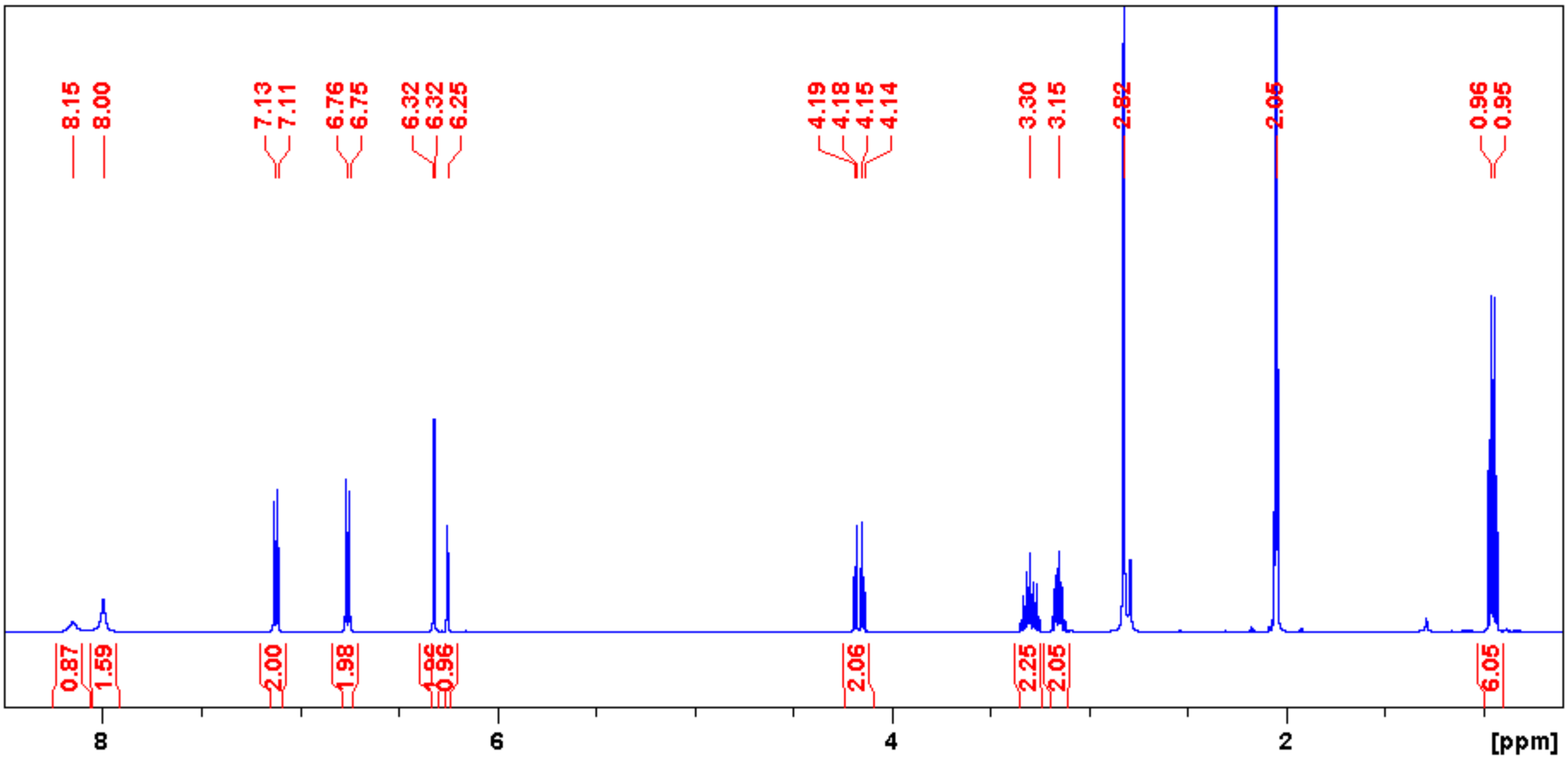

Figure S39. Compound **7**, ^{13}C, APT NMR spectrum.

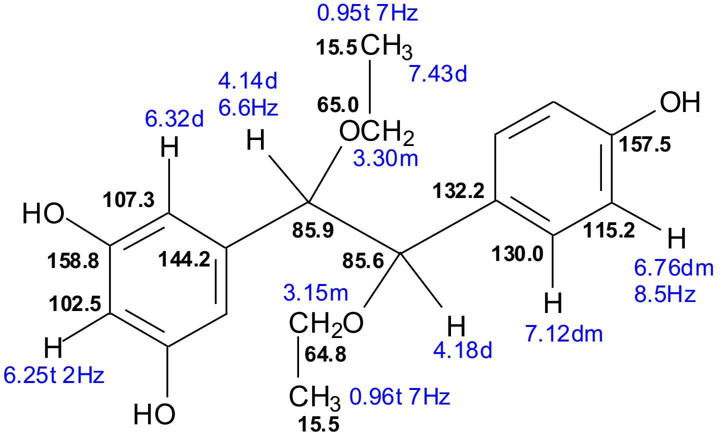

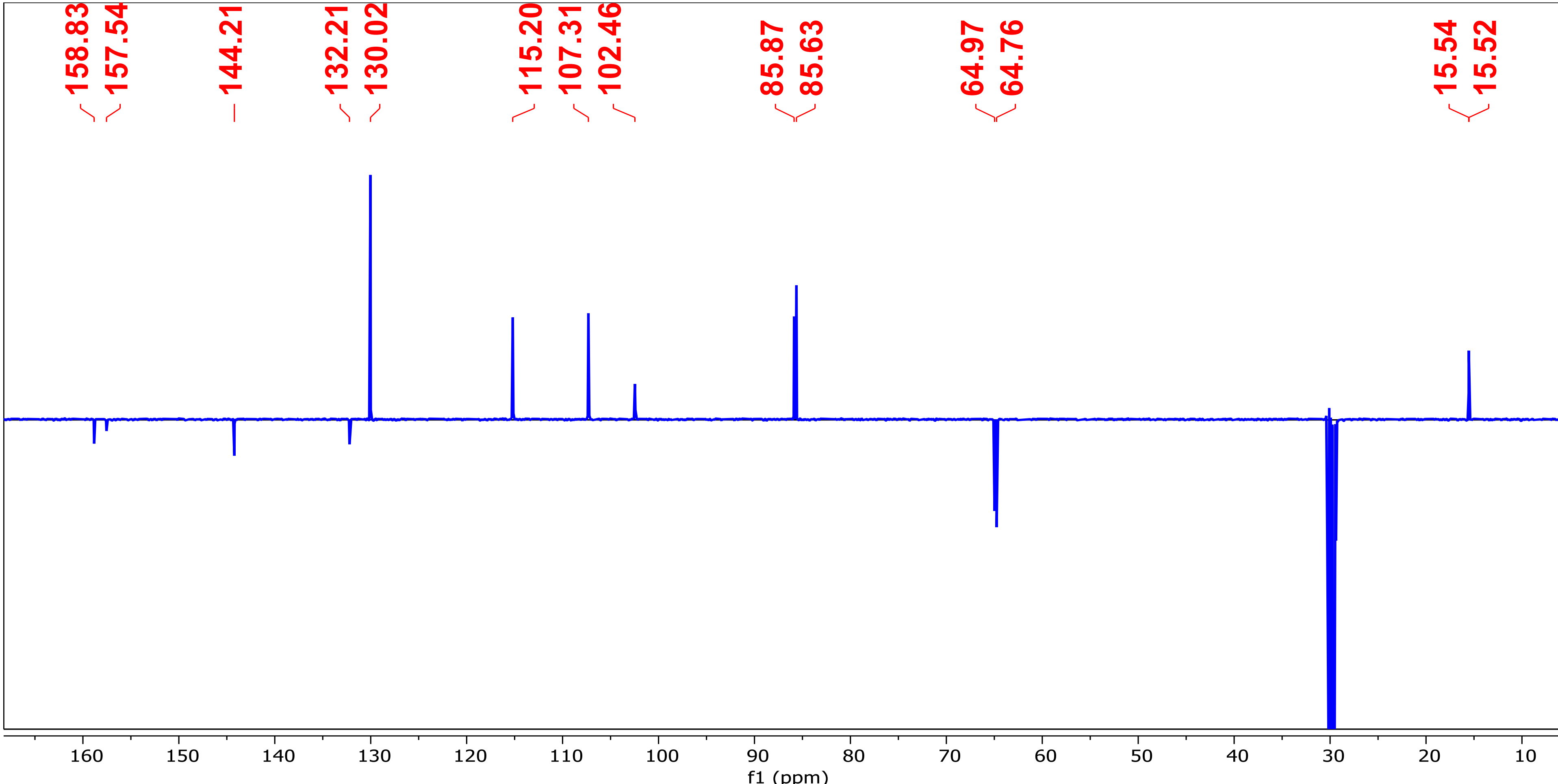

Figure S40. Compound **7**, NOESY spectrum and NOESY section.

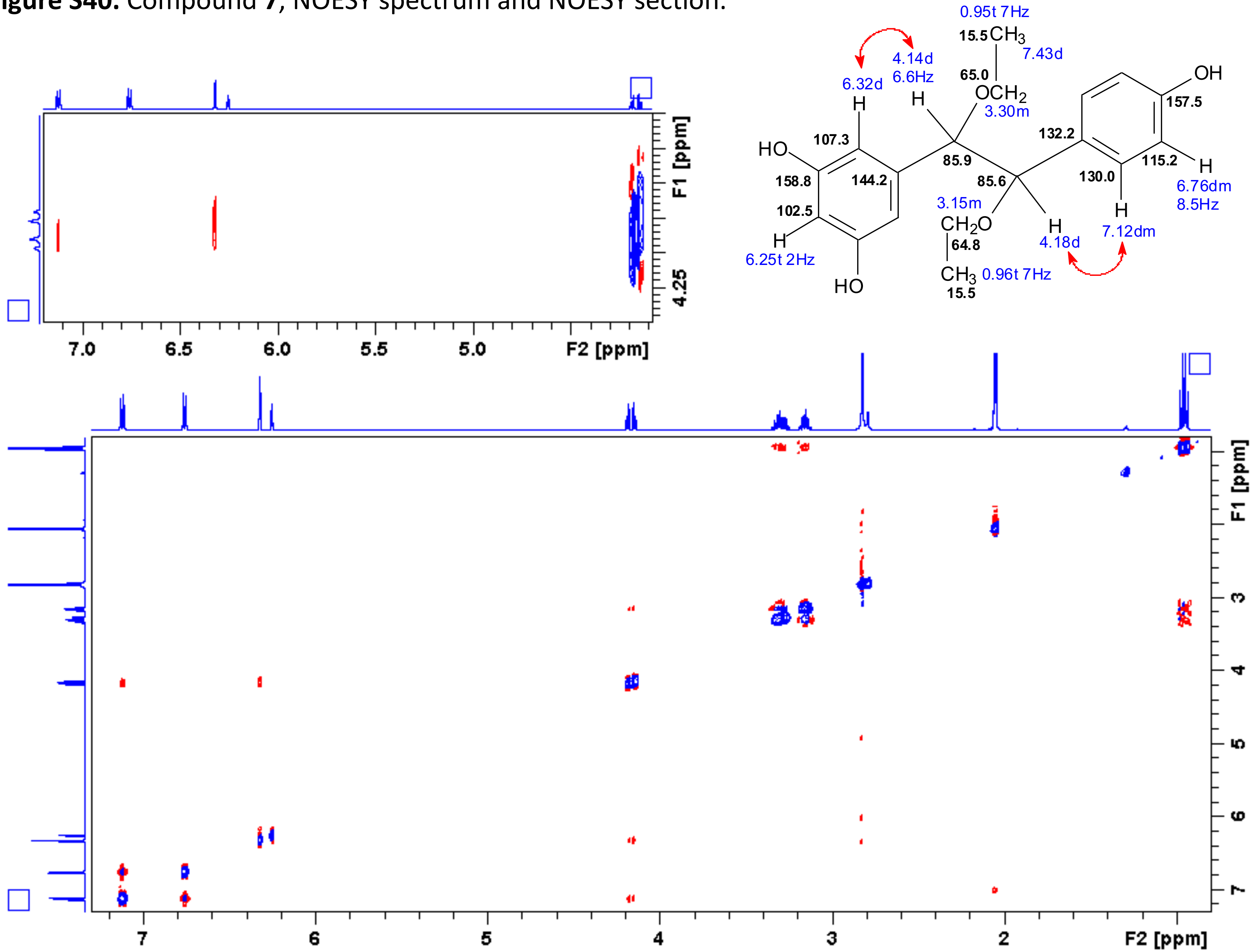

Figure S41. Compound **7**, HSQC and HMBC spectra.

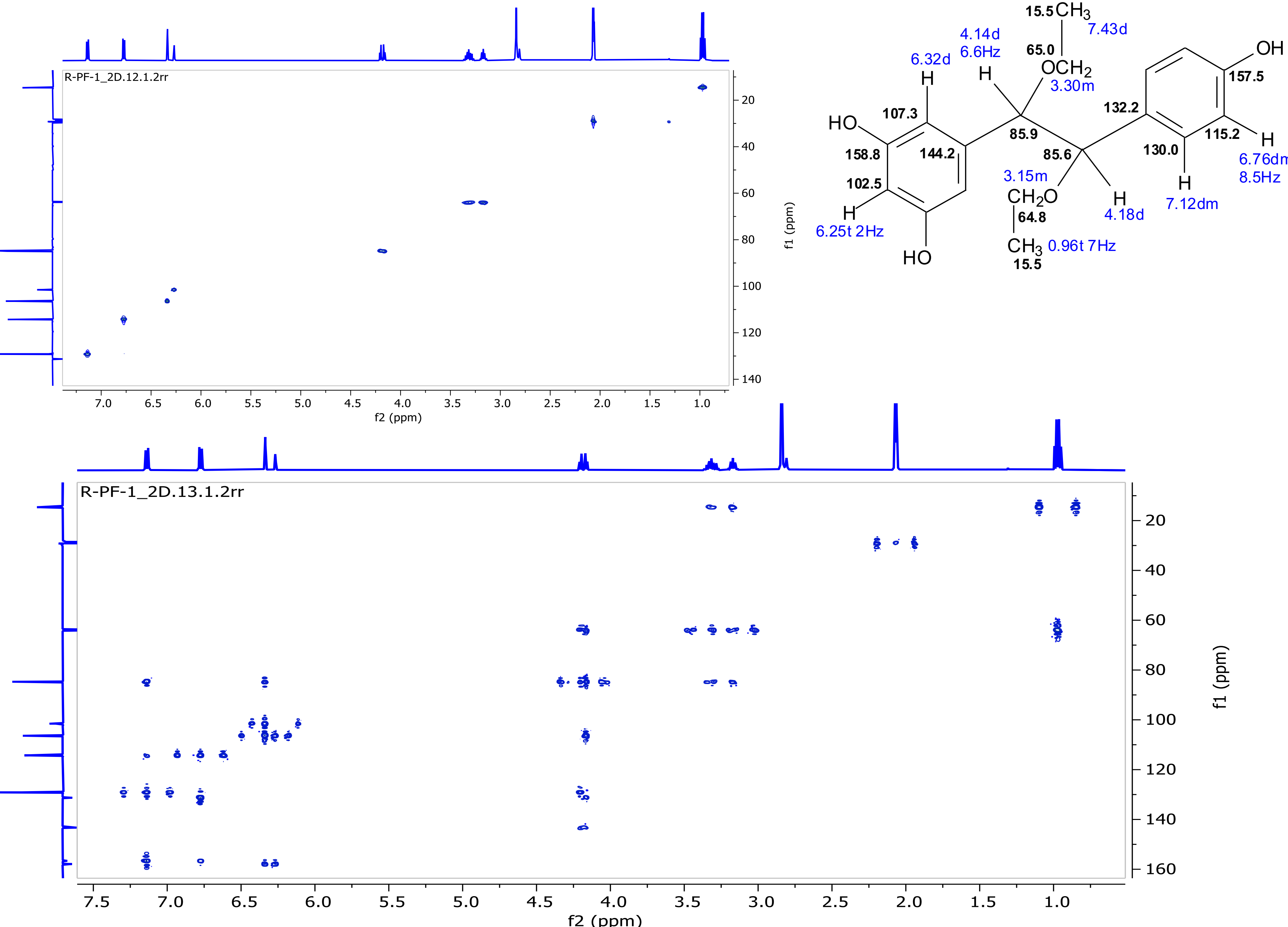

Figure S42. Compound **7**, COSY spectrum.

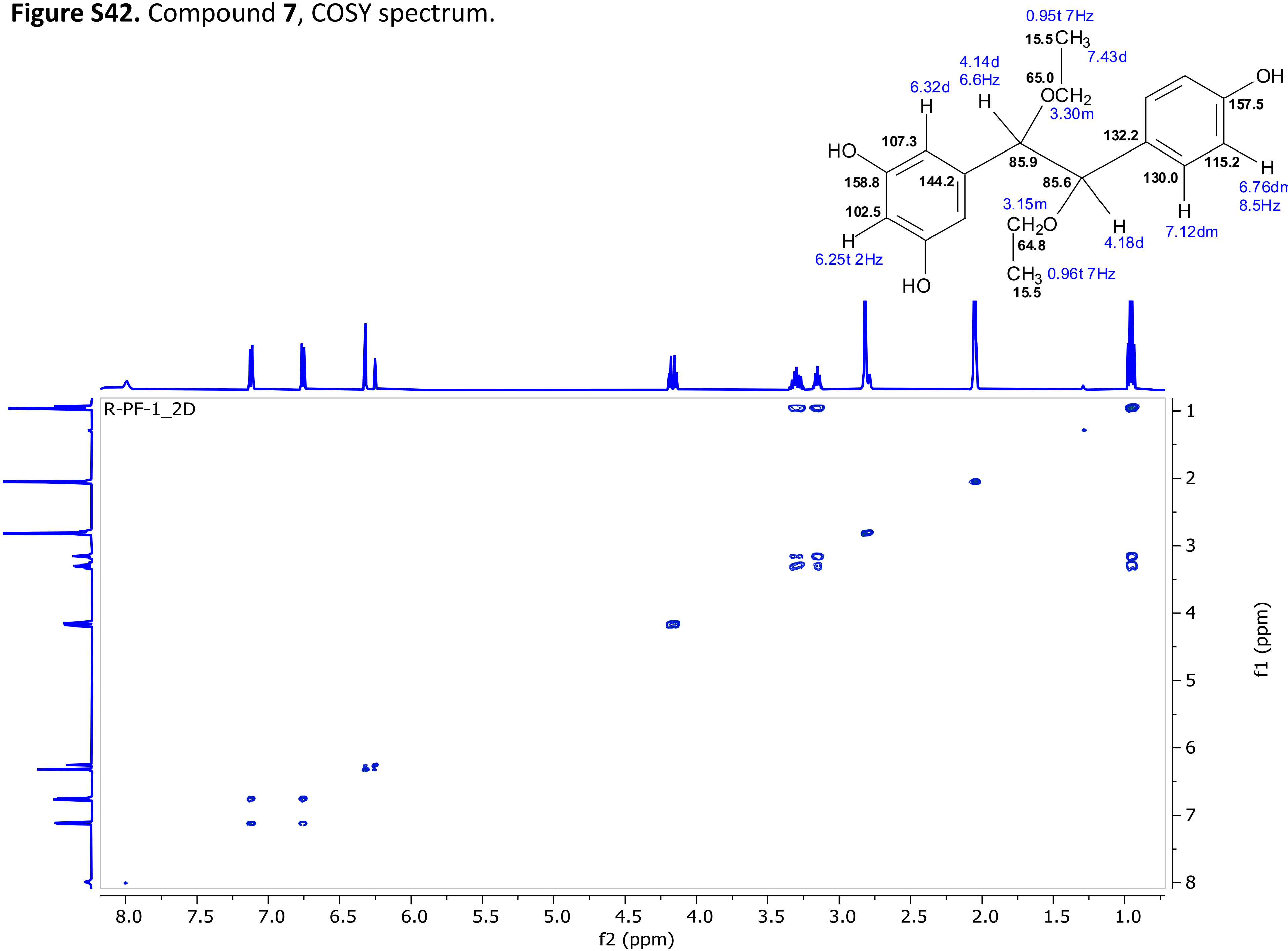

Figure S43. Compound **8**, ^{1}H NMR spectrum.

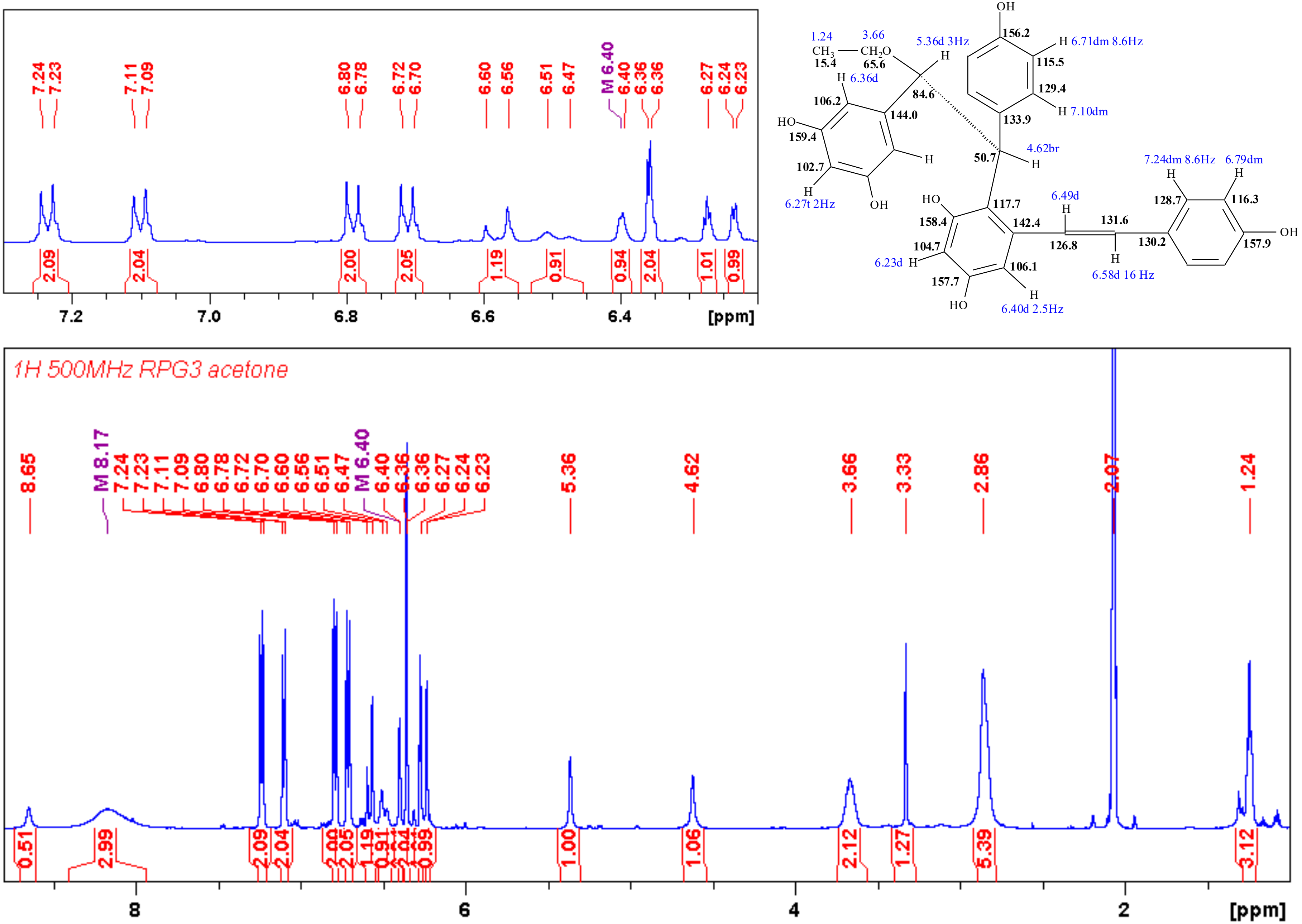

Figure S44. Compound **8**, ^{13}C, APT NMR spectrum.

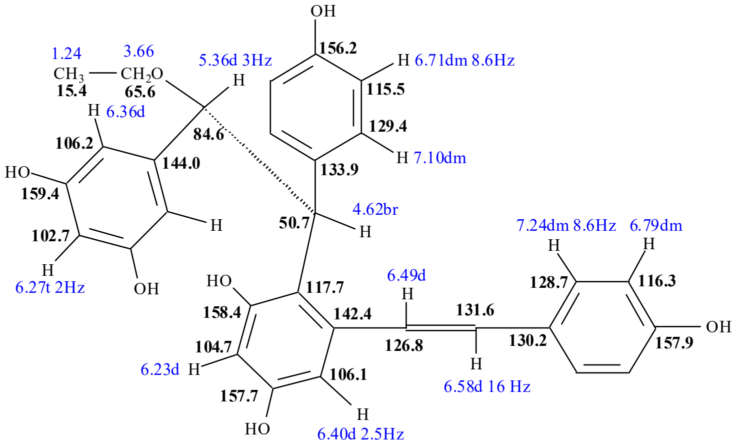

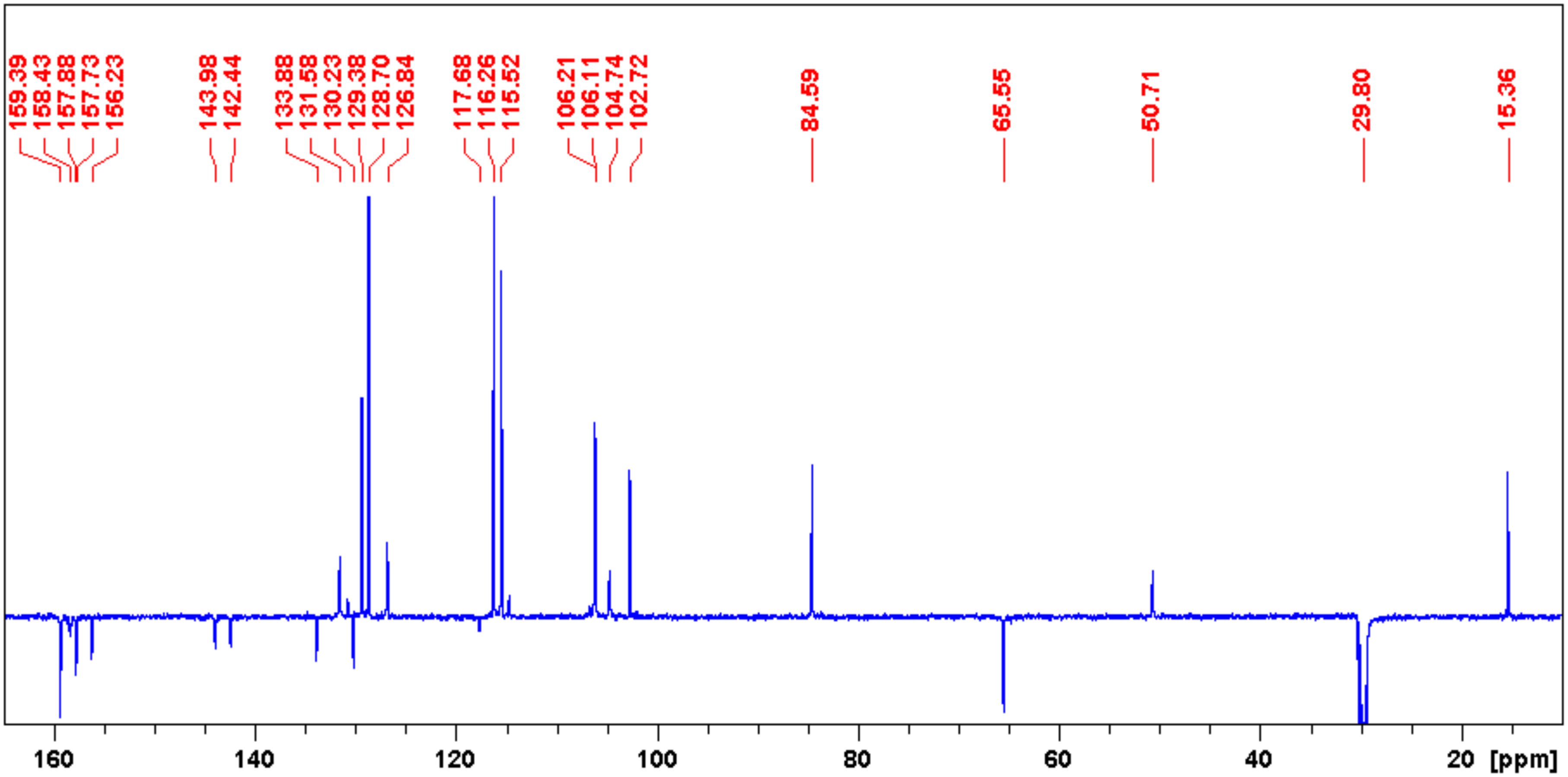

Figure S45. Compound **8**, HSQC spectrum and HSQC section.

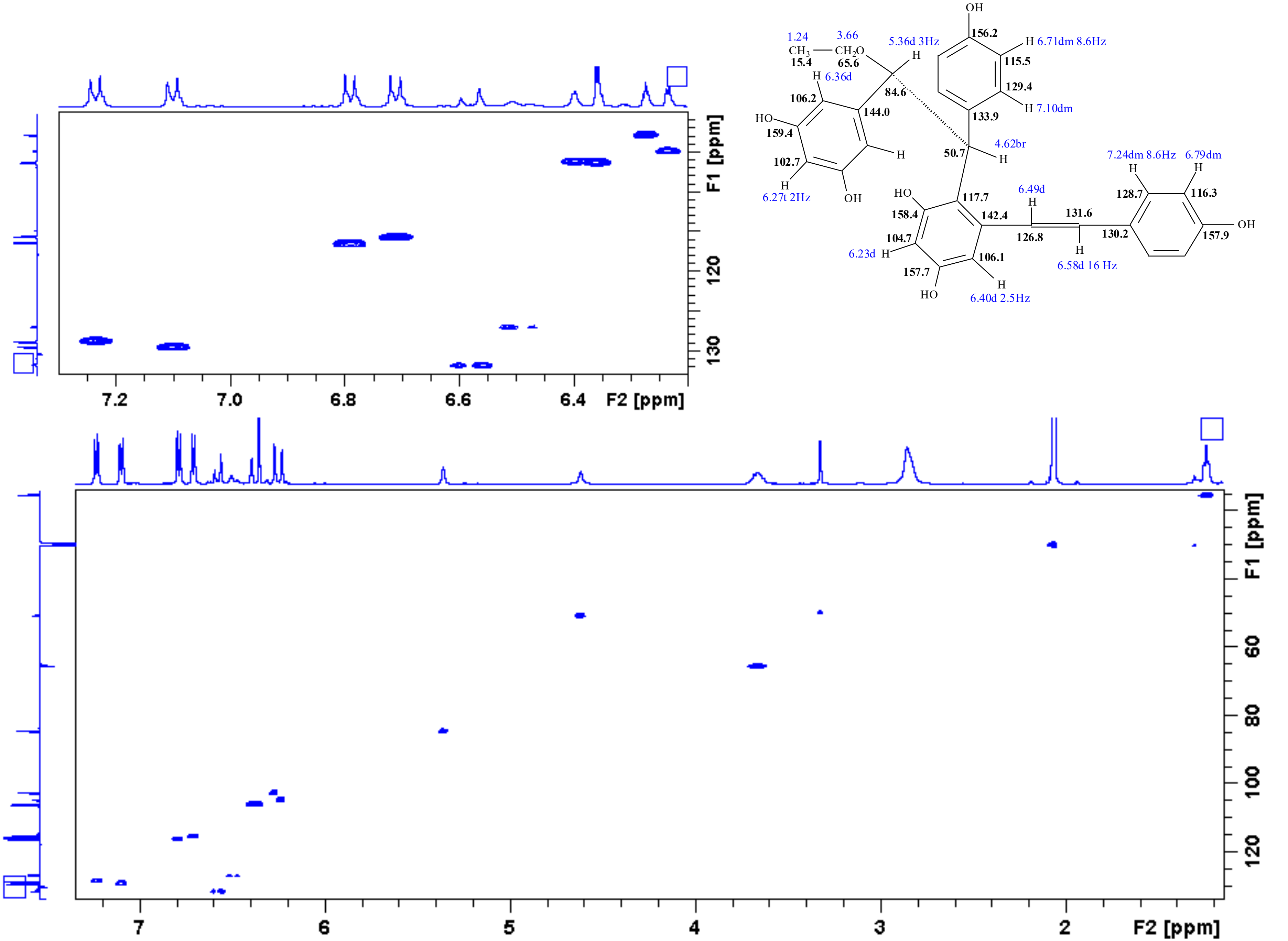

Figure S46. Compound **8**, HMBC spectrum and HMBC section; arrows indicate characteristic responses.

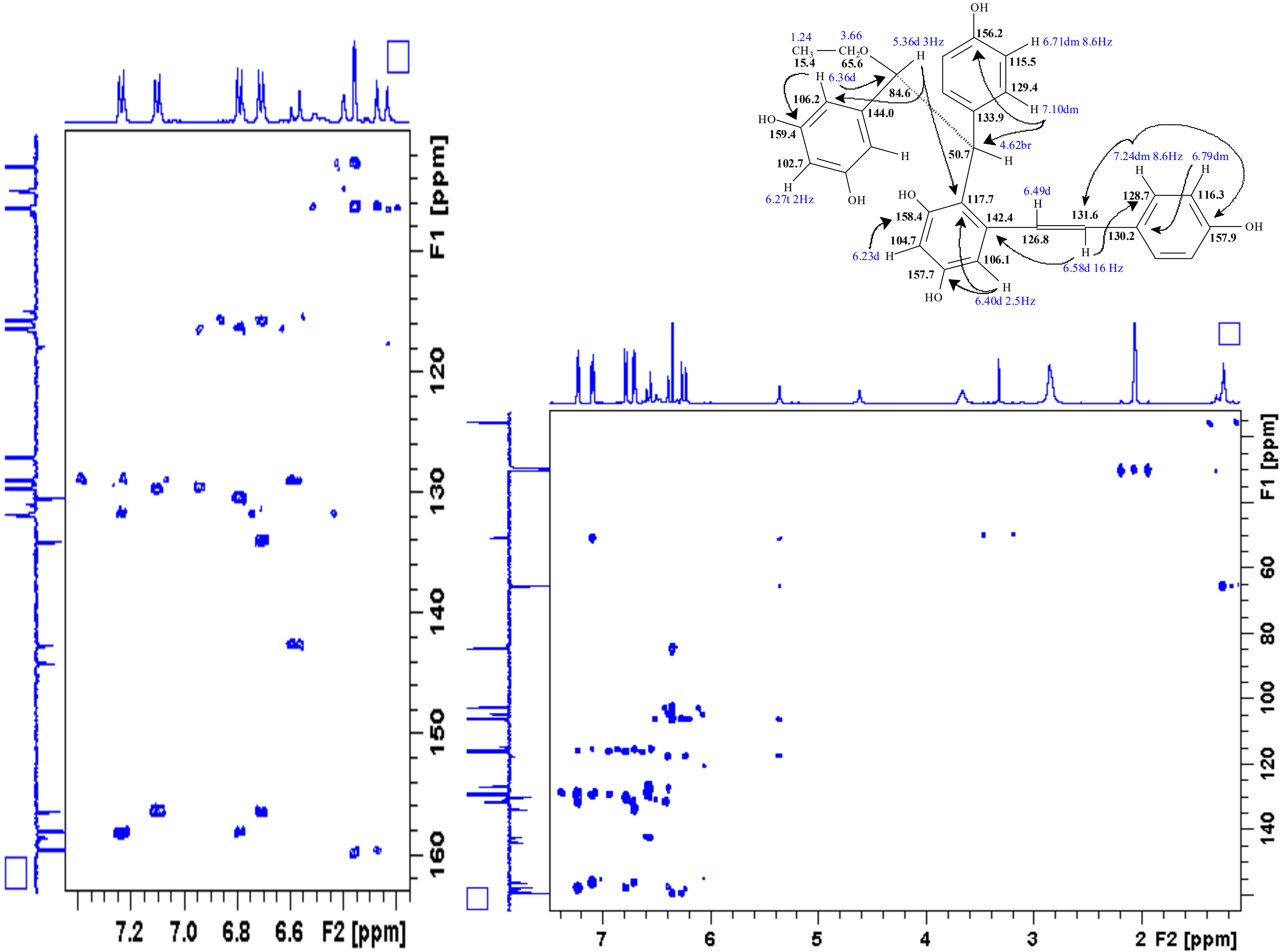

Figure S47. Compound **8**, COSY spectrum and COSY aromatic section.

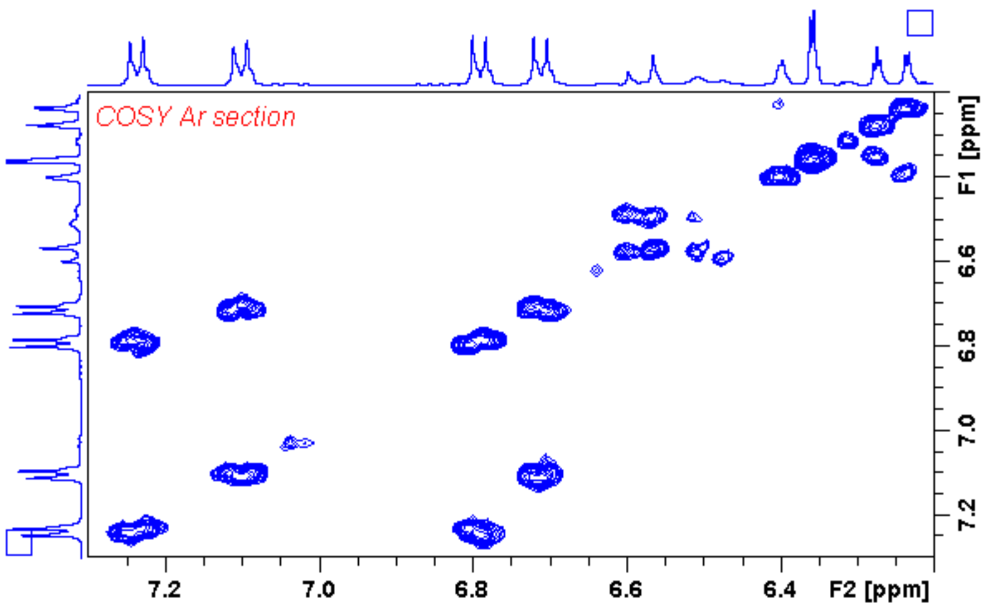

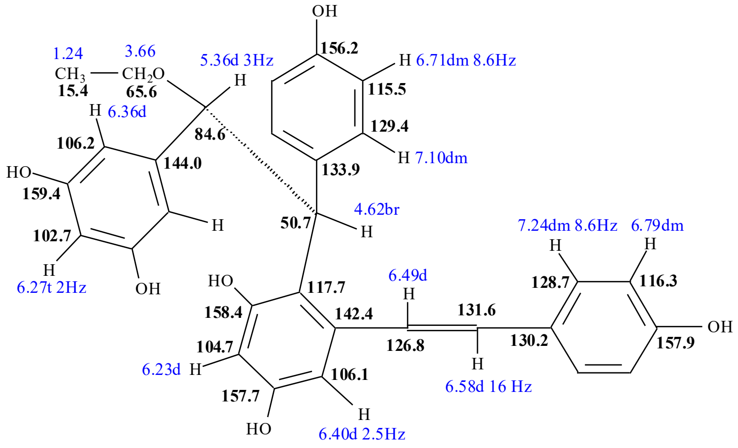

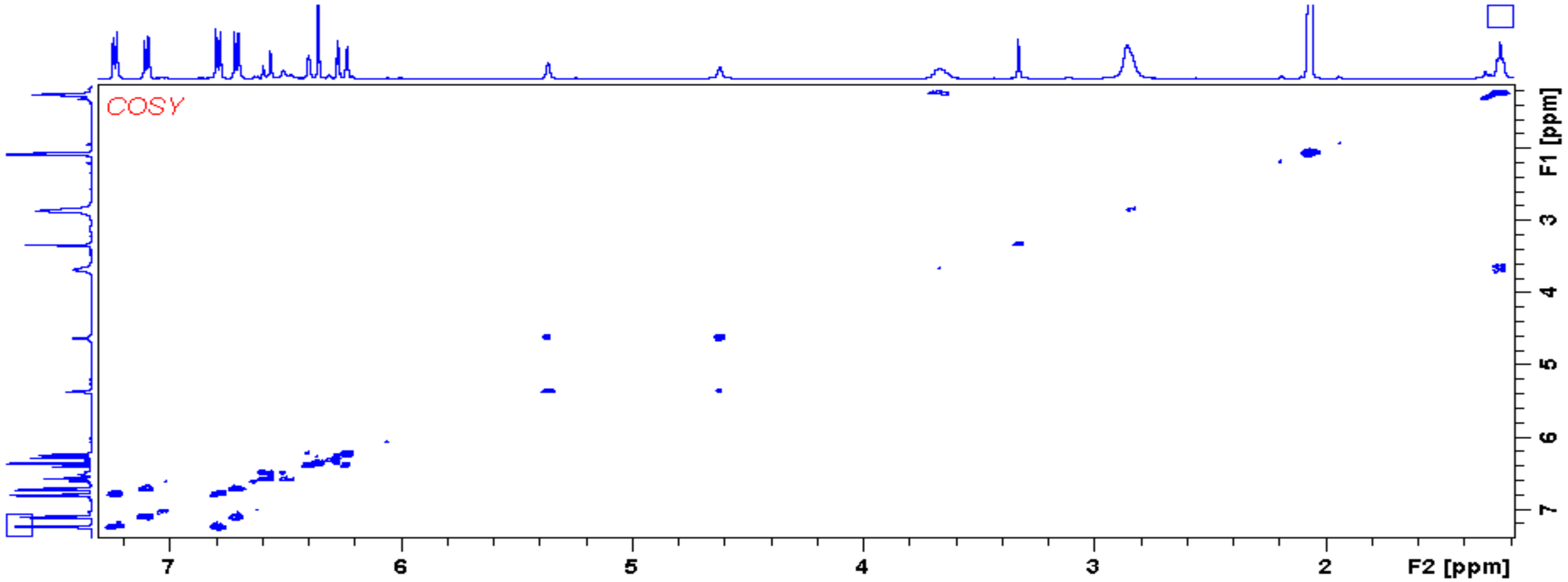

Figure S48. Compound **8**, NOESY; arrows indicate steric proximities, and assign configuration and the dominant conformer.

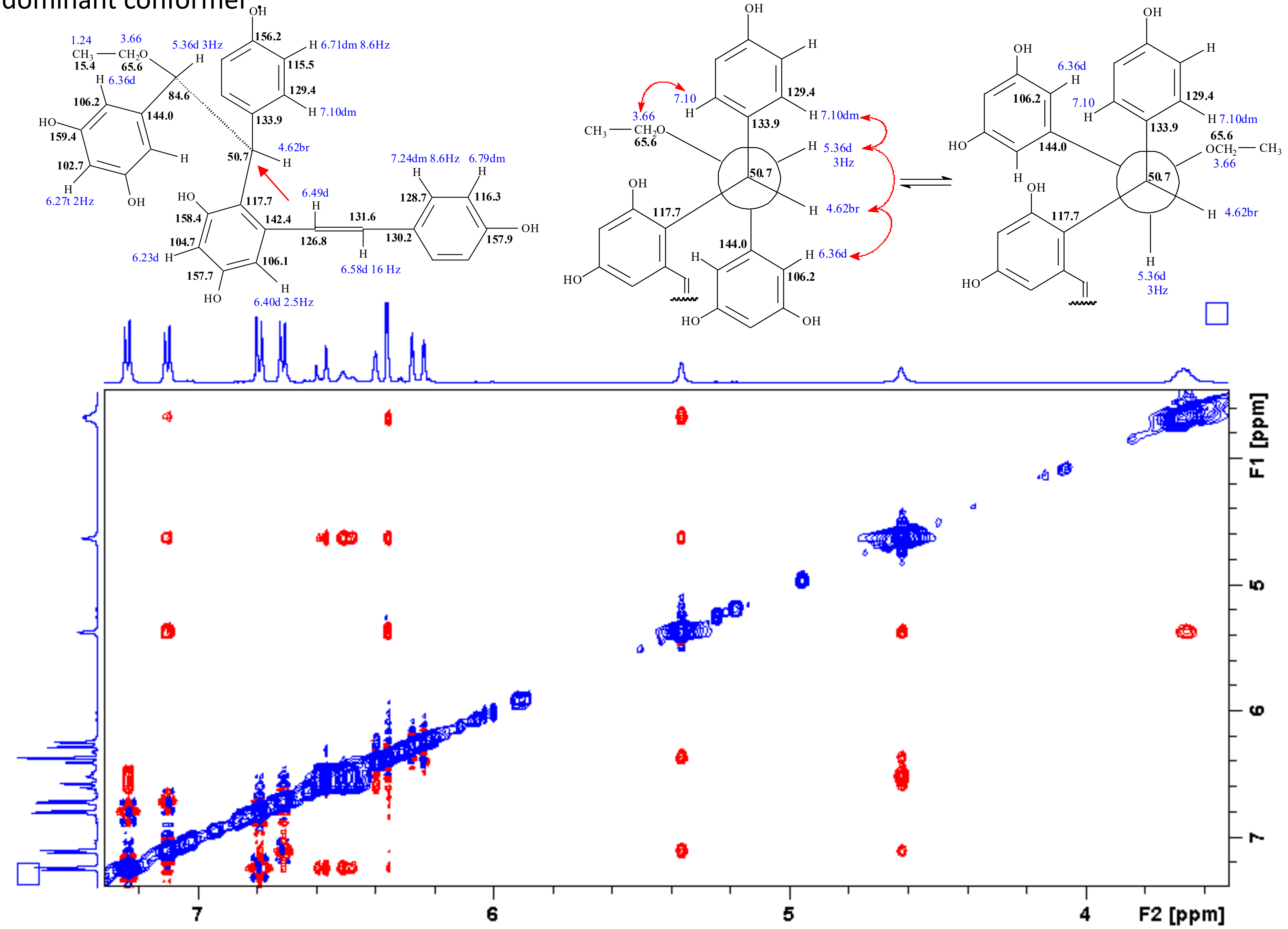

Figure S49. Compound **9**, ^{1}H NMR spectrum.

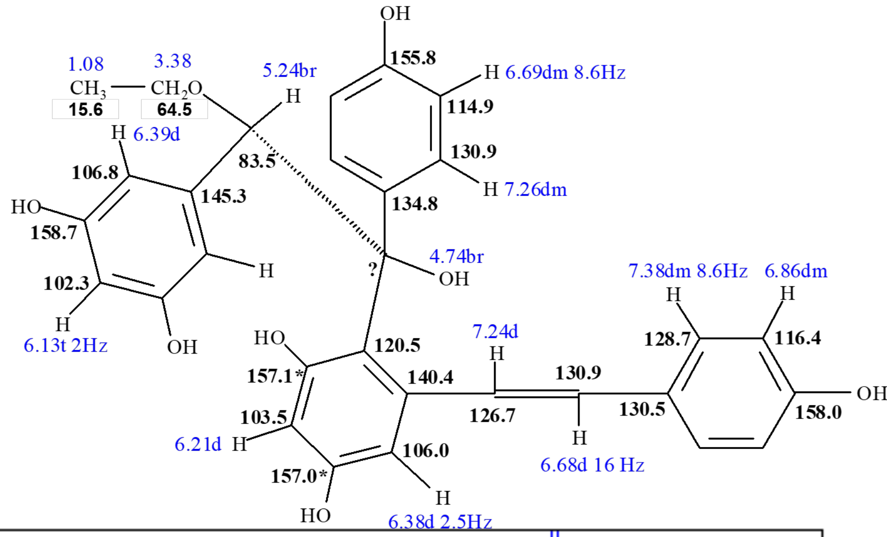

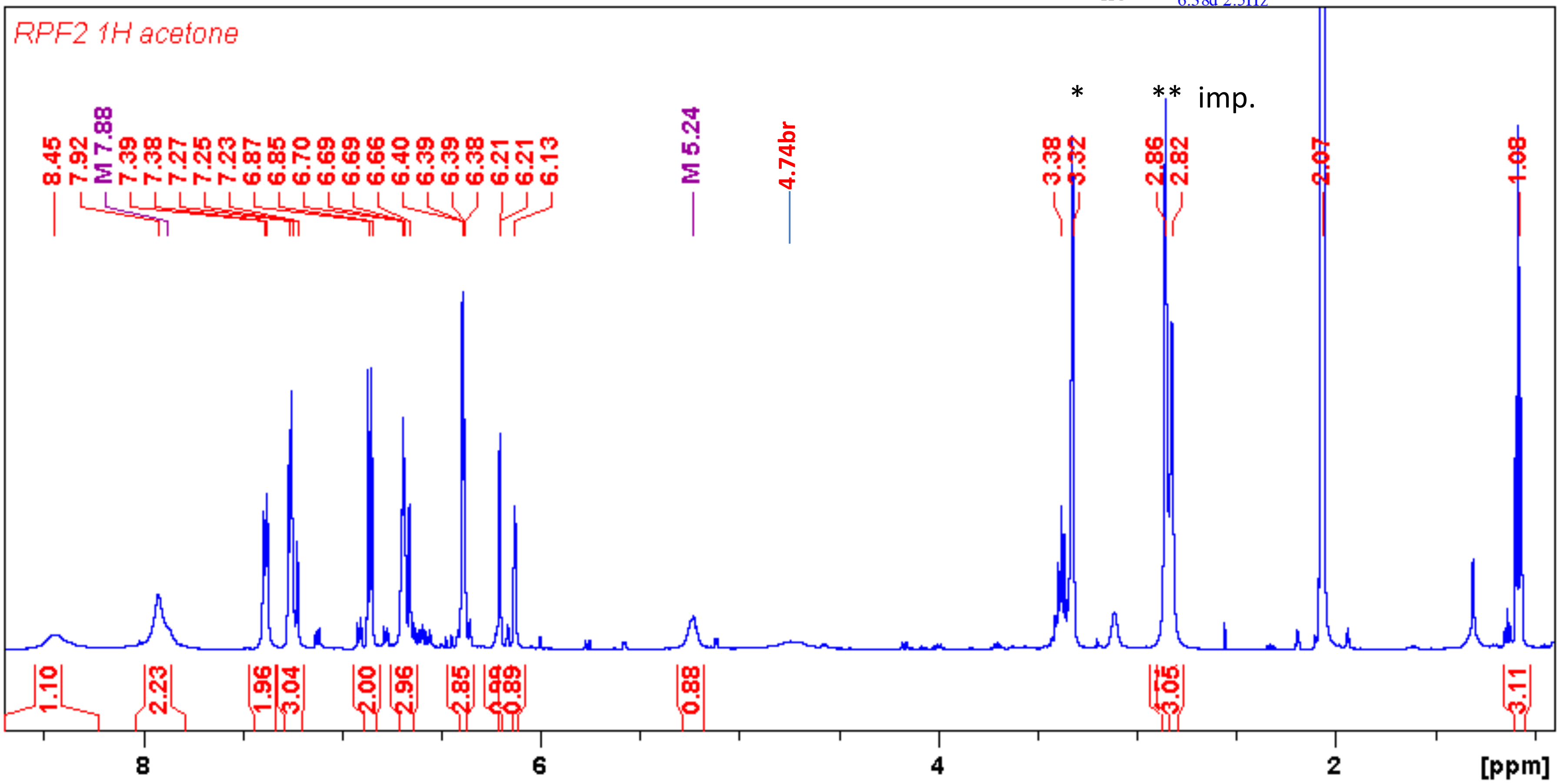

Figure S50. Compound **9**, ^{13}C, APT NMR spectrum.

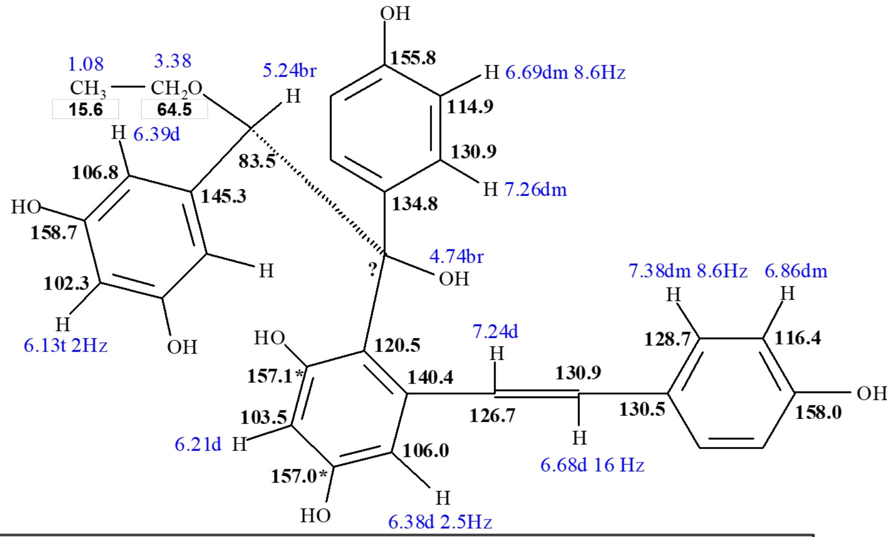

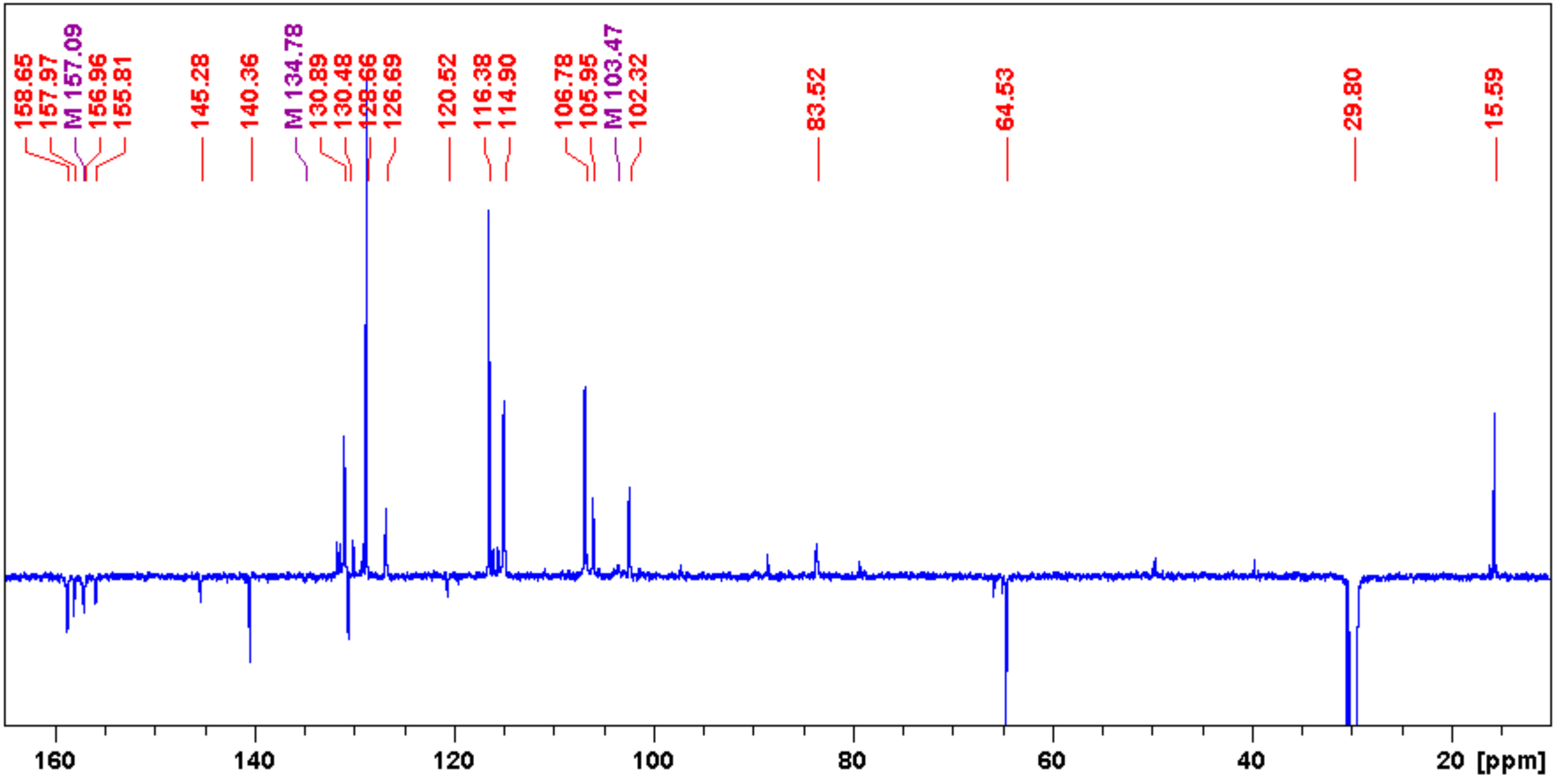

Figure S51. Compound **9**, HSQC spectrum and HSQC section.

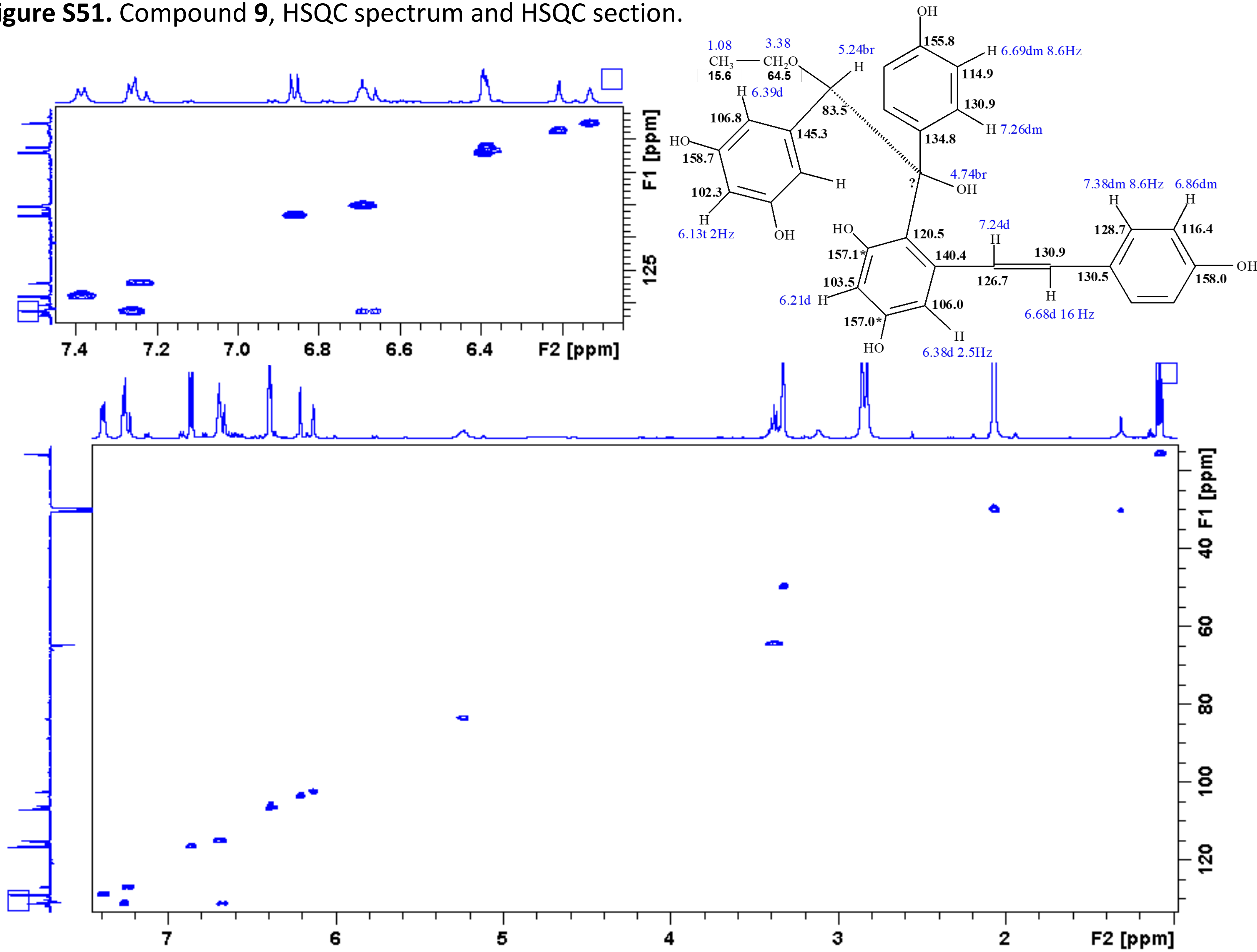

Figure S52. Compound **9**, HMBC spectrum.

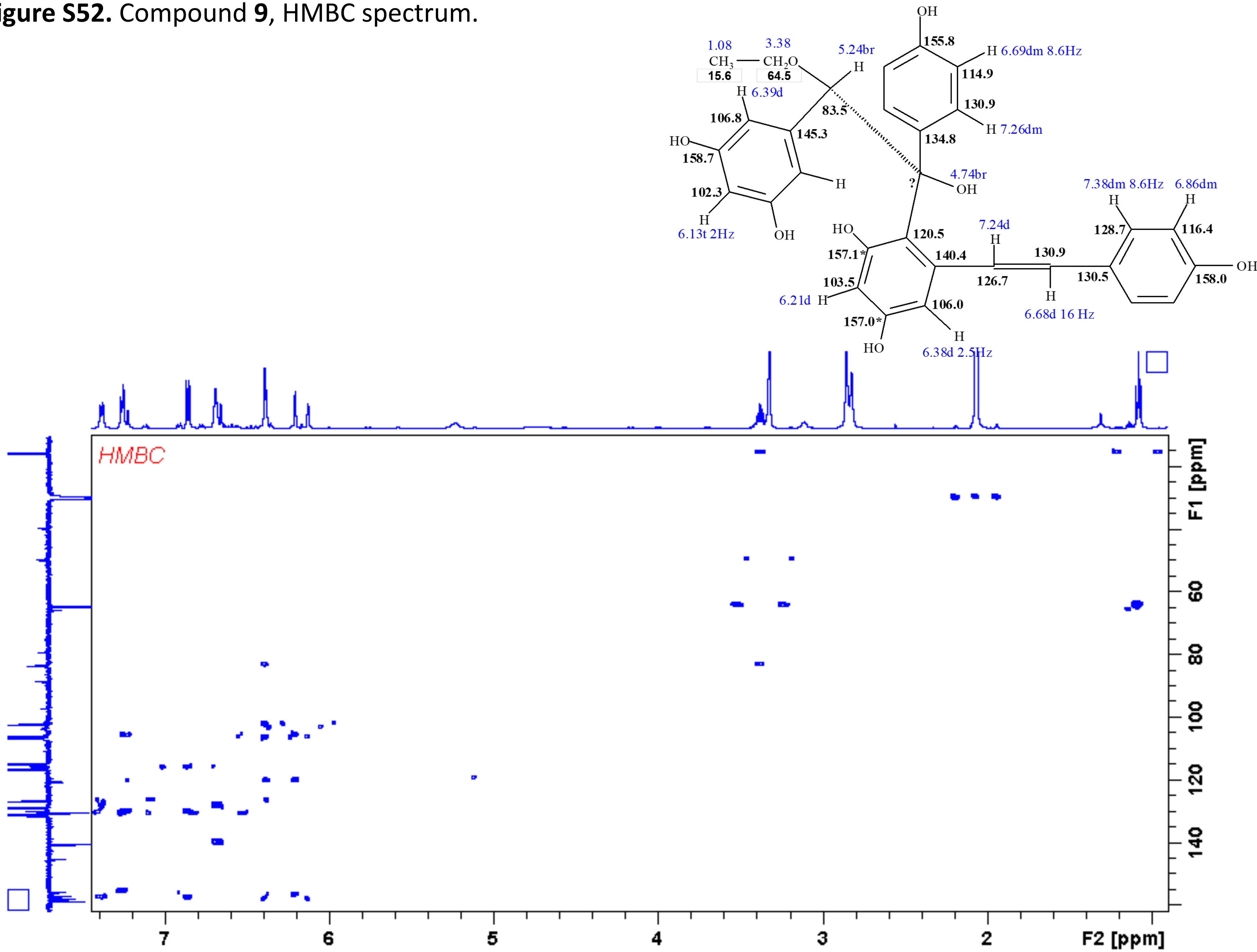

Figure S53. Compound **9**, NOESY spectrum.

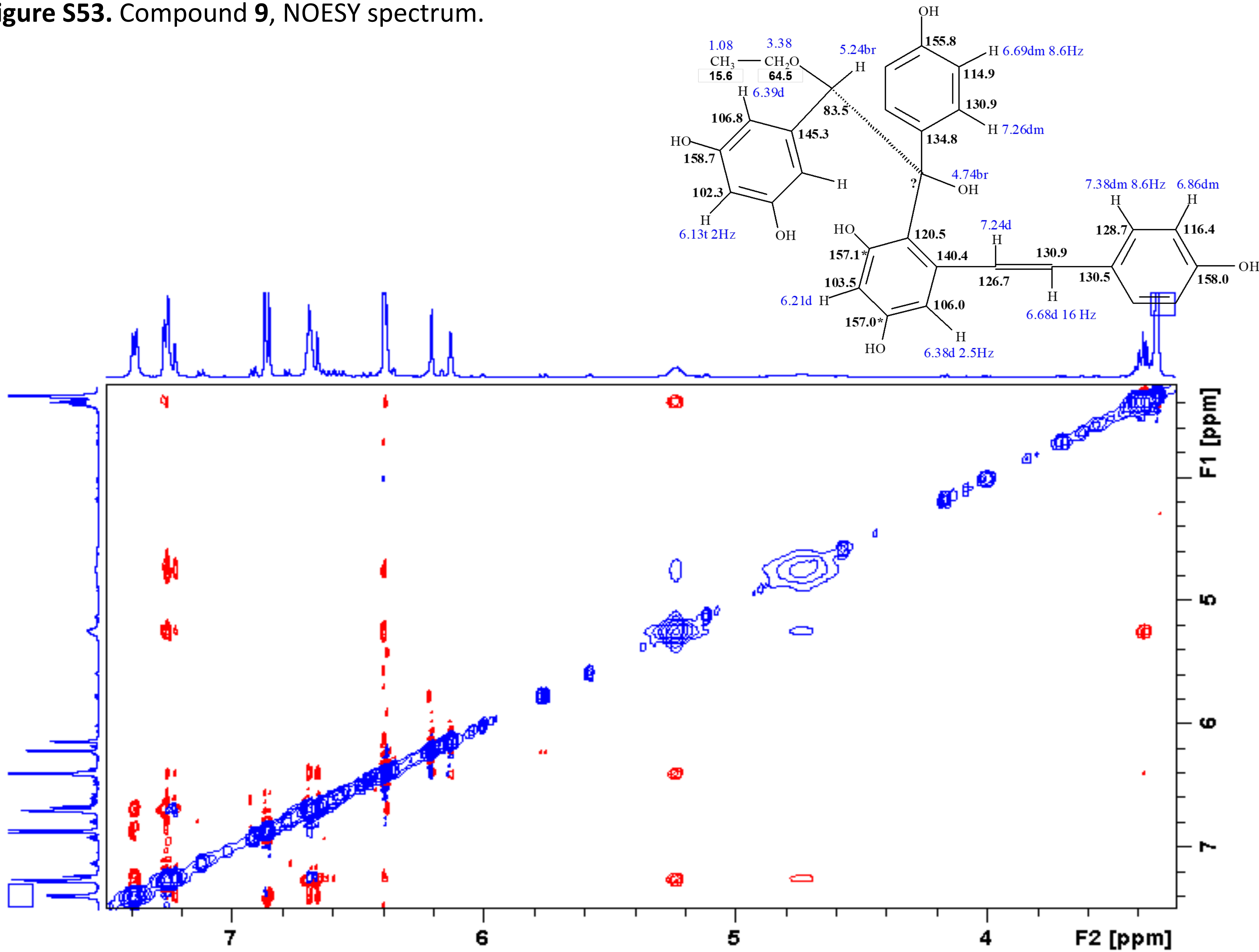

Figure S54. Compound **9**, HSQC spectrum and HSQC section, DMSO-d$_6$, T=80°C.

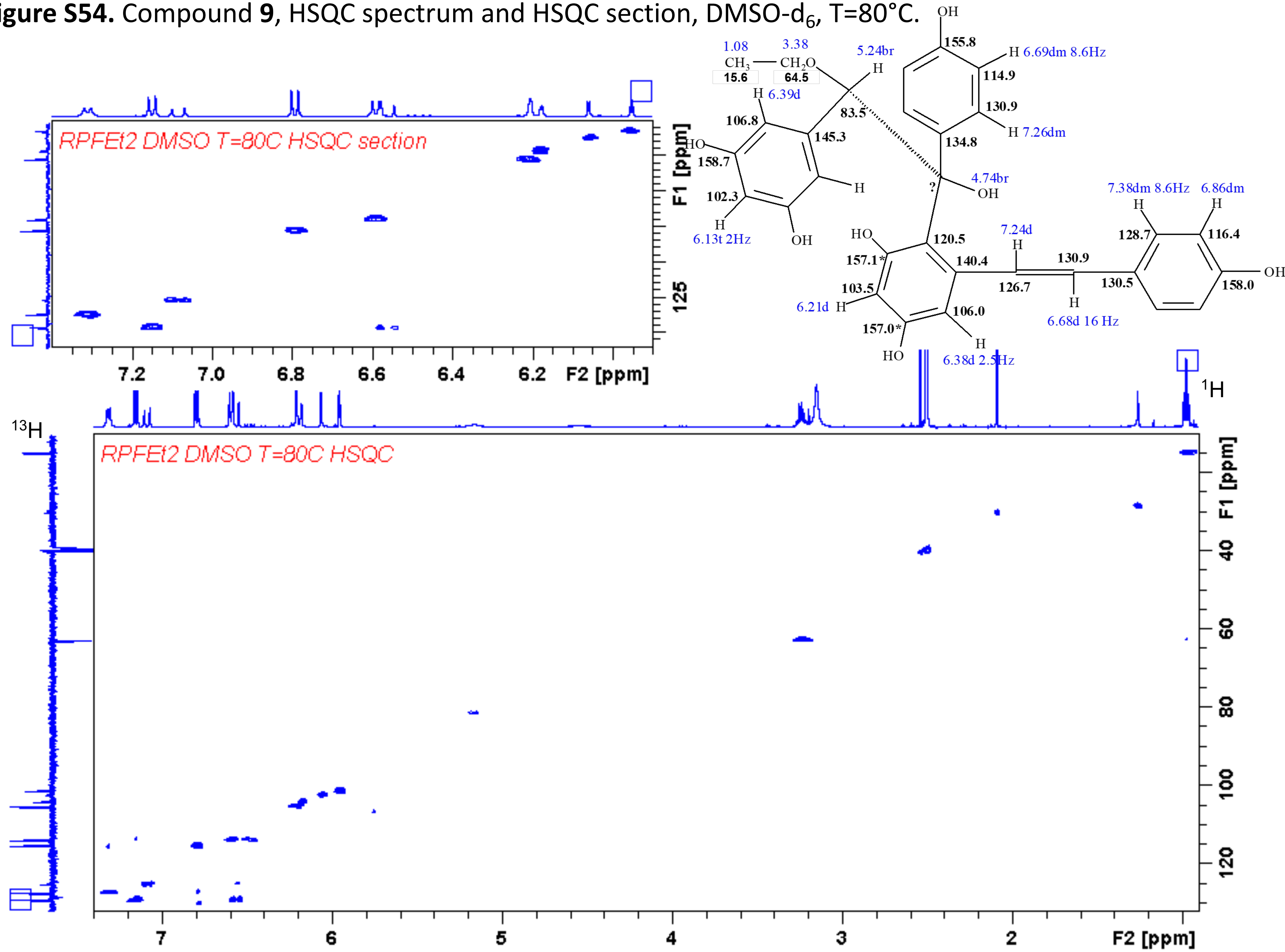

Figure S55. Compound **9**, ^{13}C, APT NMR spectrum, DMSO-d_6, T=80°C.

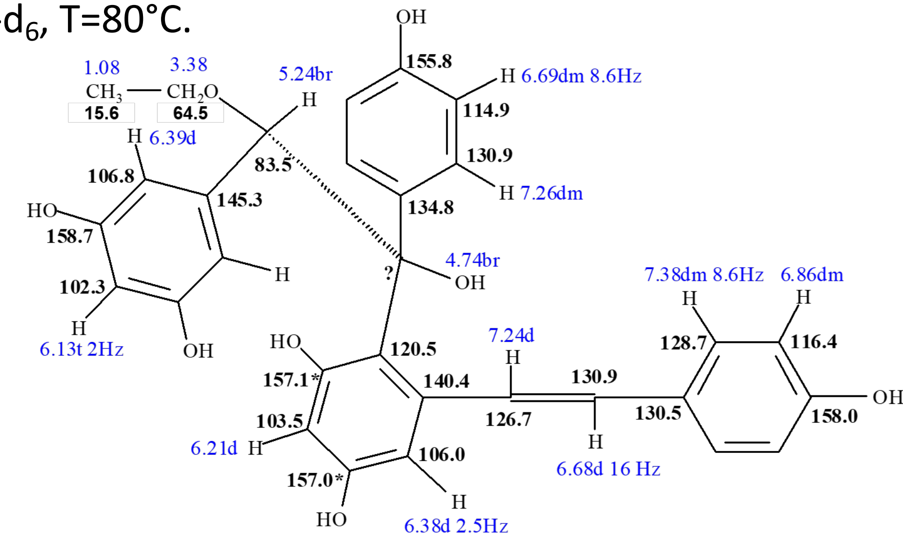

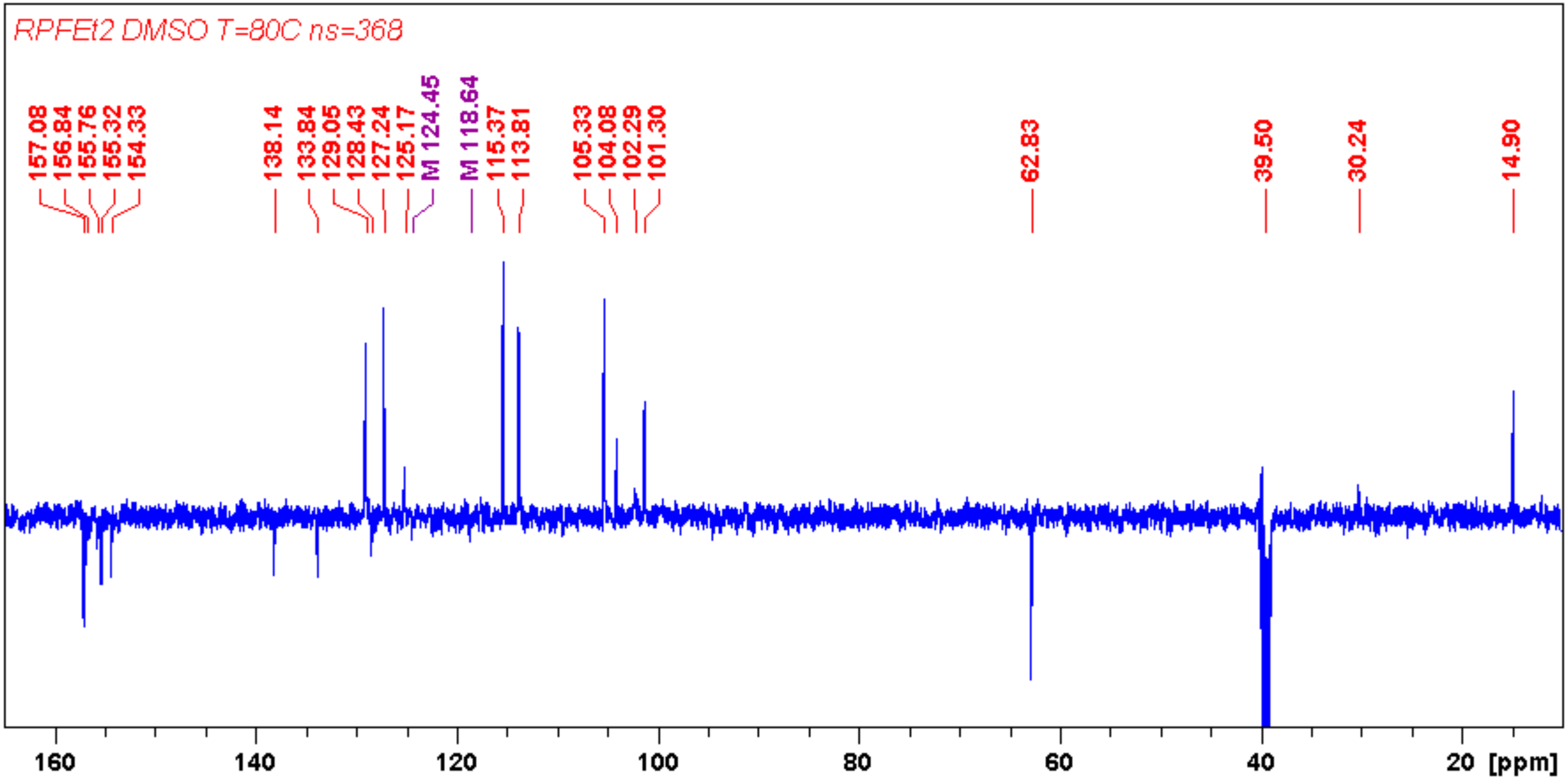

Figure S56. Compound **9**, selROE on CH (5.16 ppm) and OH (4.53 ppm) , DMSO-d_6, T=80°C.

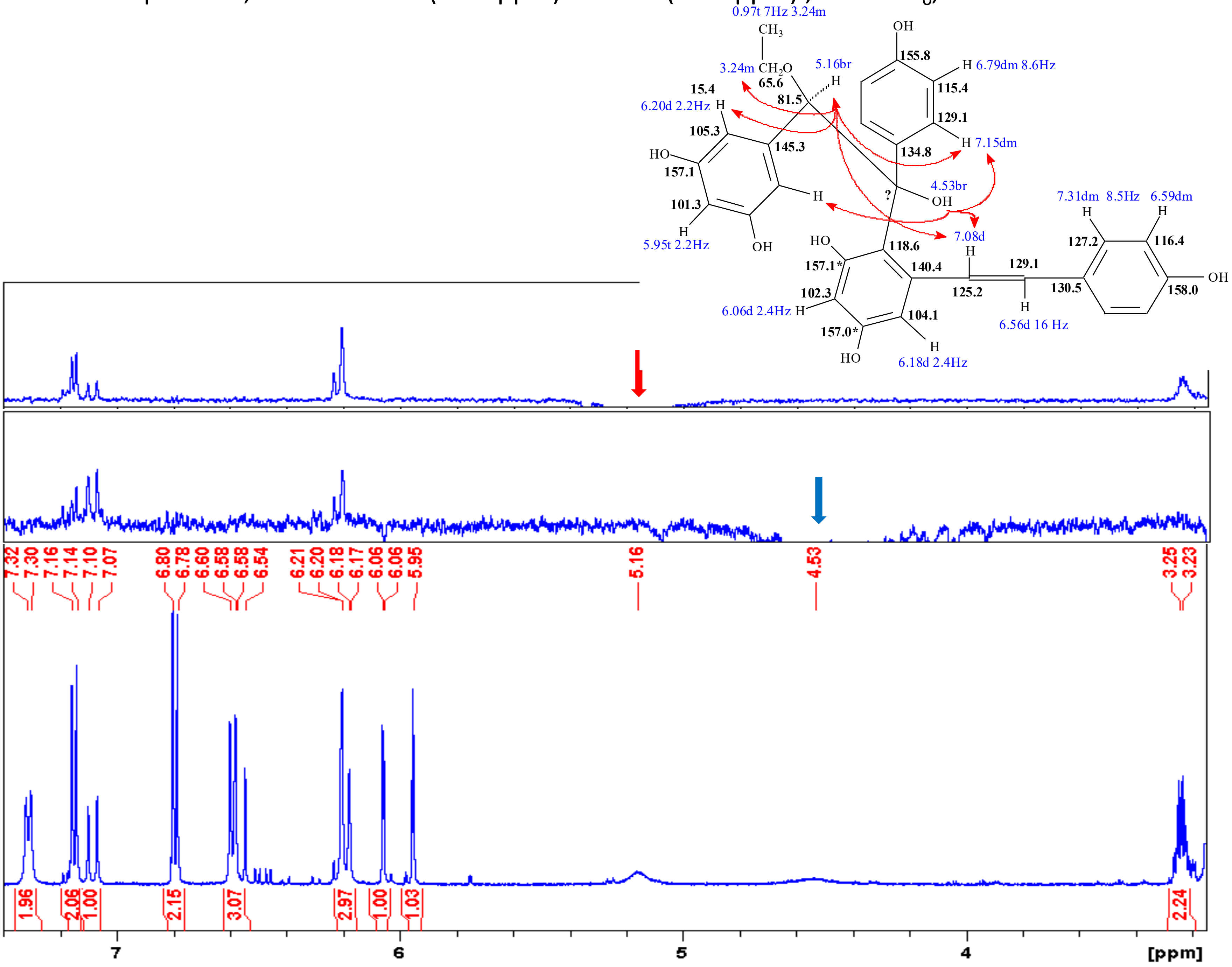

Figure S57 : Lineweaver-Burk Plot of compound 2

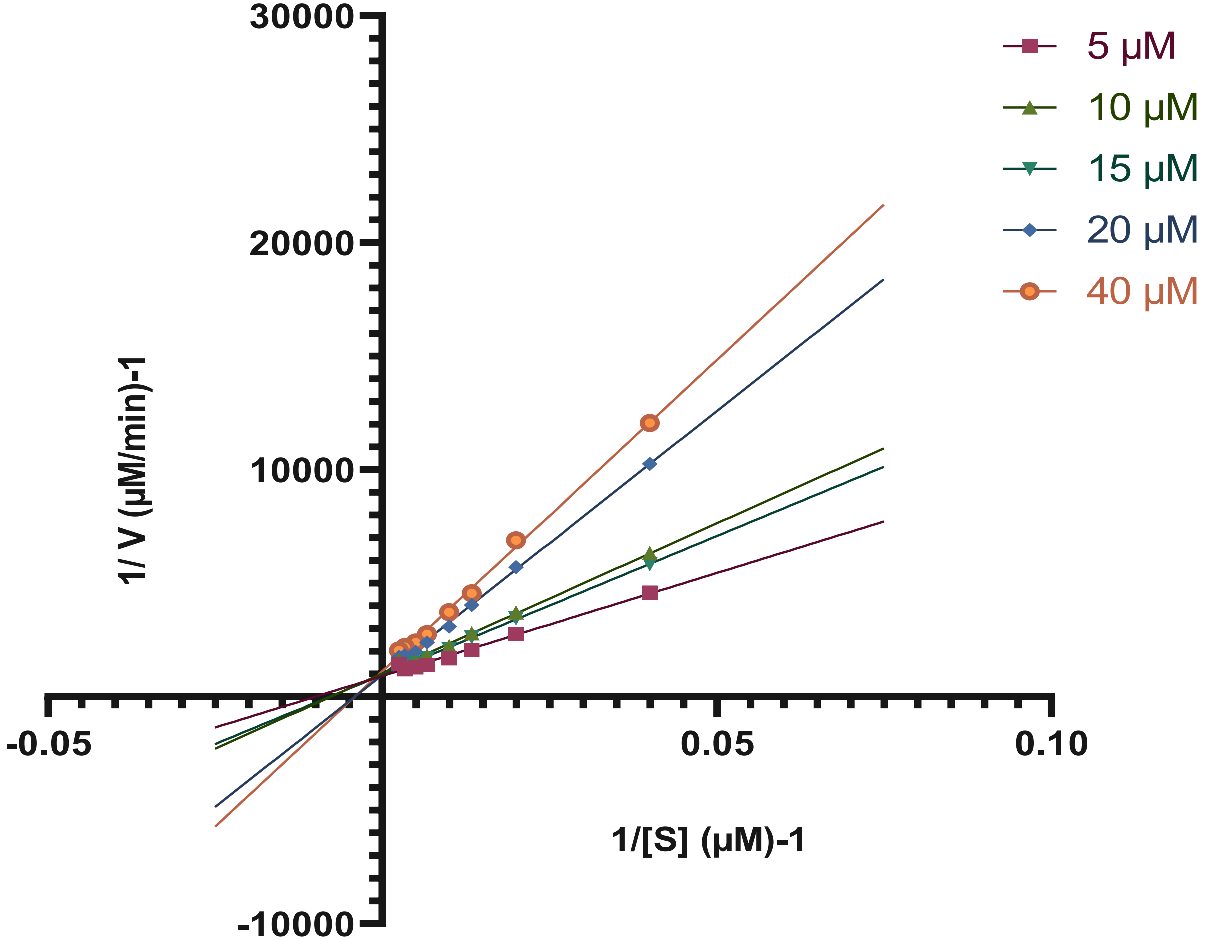

Concentration (µM)	Vmax (x 10^{-3} mM/min)	Km (µM)	Inhibition type
5	1.1	99.5	Competitive inhibition
10	0.98	129.7	
15	1.04	126.5	
20	1.05	244.0	
40	0.89	244.0	

Figure S58 : Lineweaver-Burk Plot of compound 3

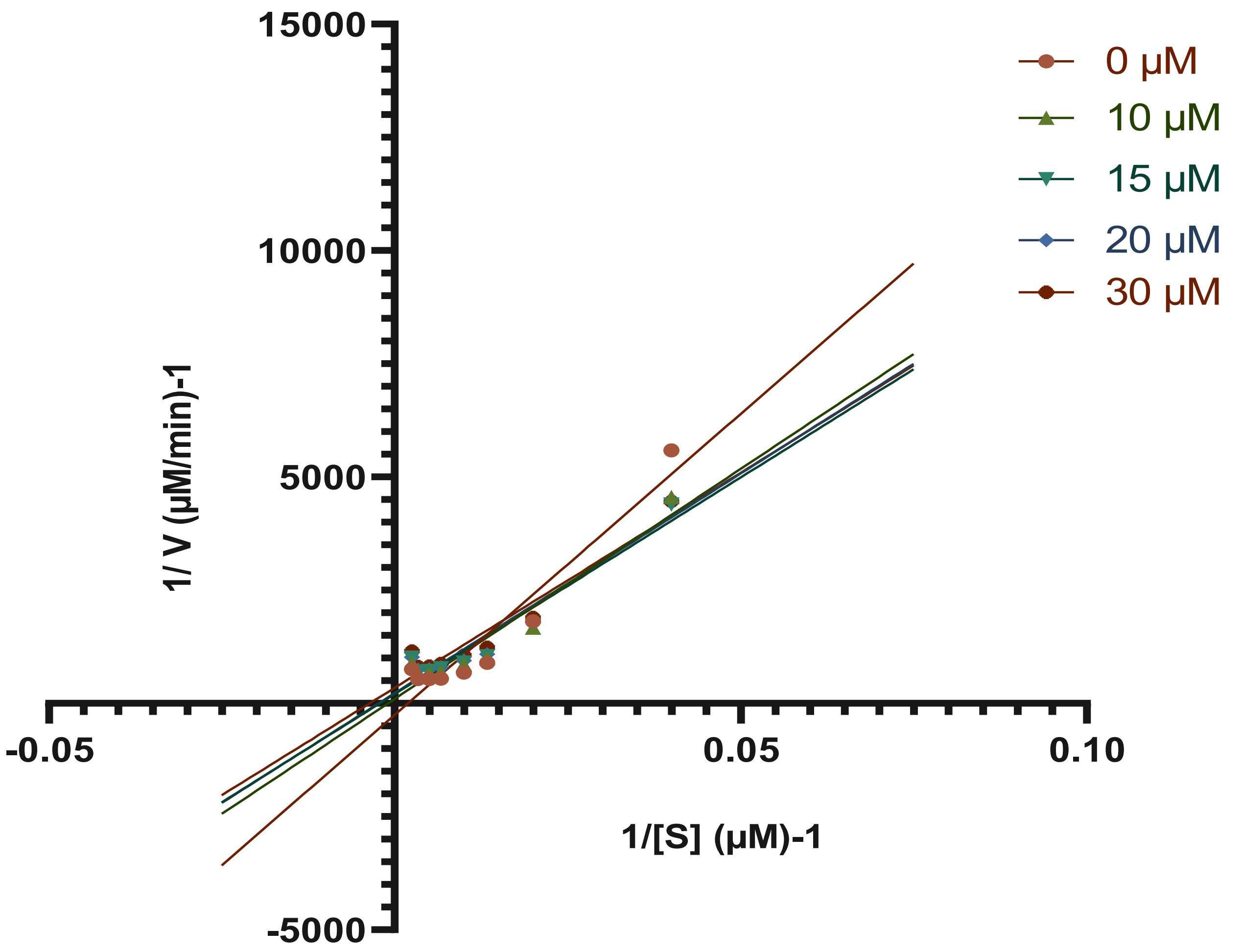

Concentration (µM)	Vmax (x 10^{-3} mM/min)	Km (µM)	Inhibition type
0	-3.9	-521.8	Mixed inhibition
10	10.1	1030.7	
15	4.8	463.1	
20	4.4	427.4	
30	2.9	275.4	

Figure S59 : Lineweaver-Burk Plot of compound 4

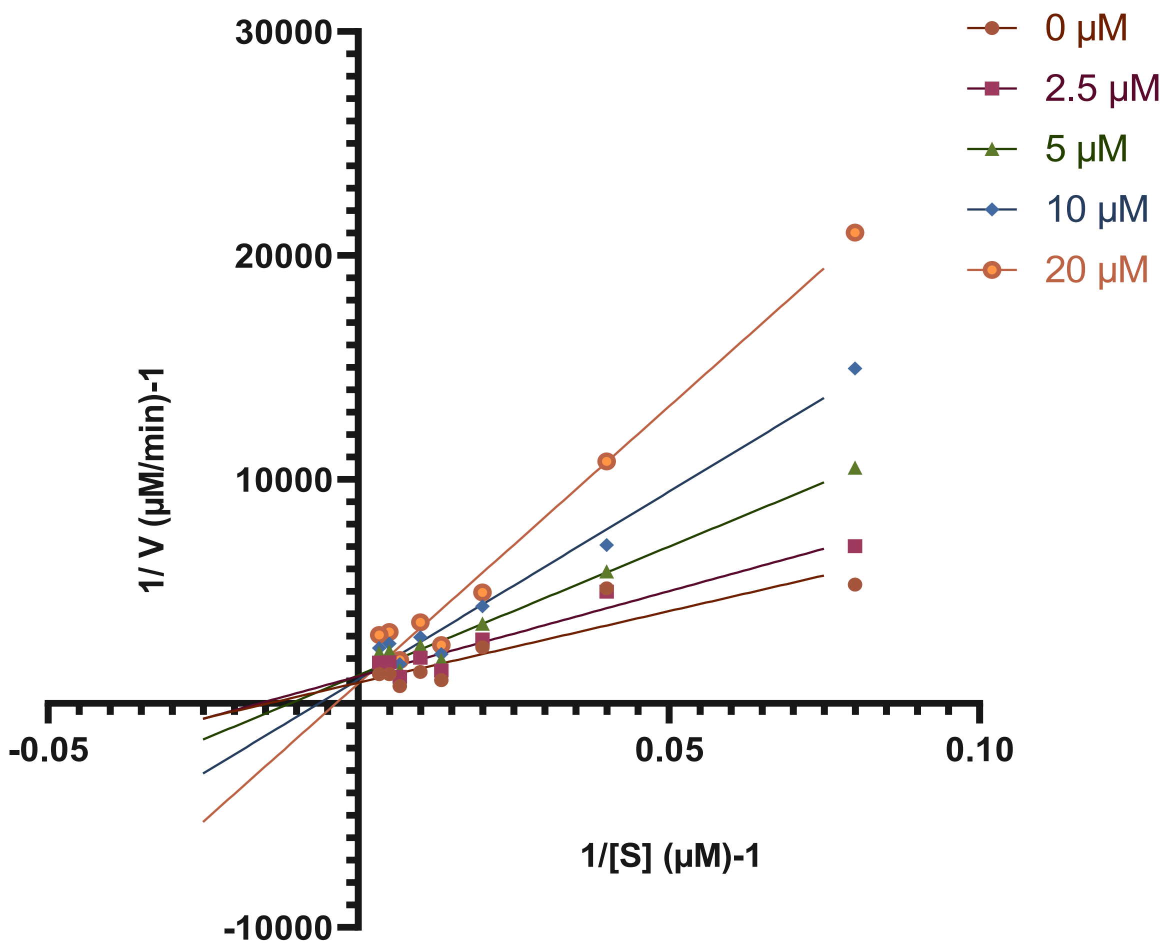

Concentration (µM)	Vmax (x 10^{-3} mM/min)	Km (µM)	Inhibition type
0	1.1	68.7	Competitive inhibition
2.5	0.8	62.5	
5	0.8	91.0	
10	0.9	157.1	
20	1.1	276.9	

Figure S60 : Lineweaver-Burk Plot of compound 5

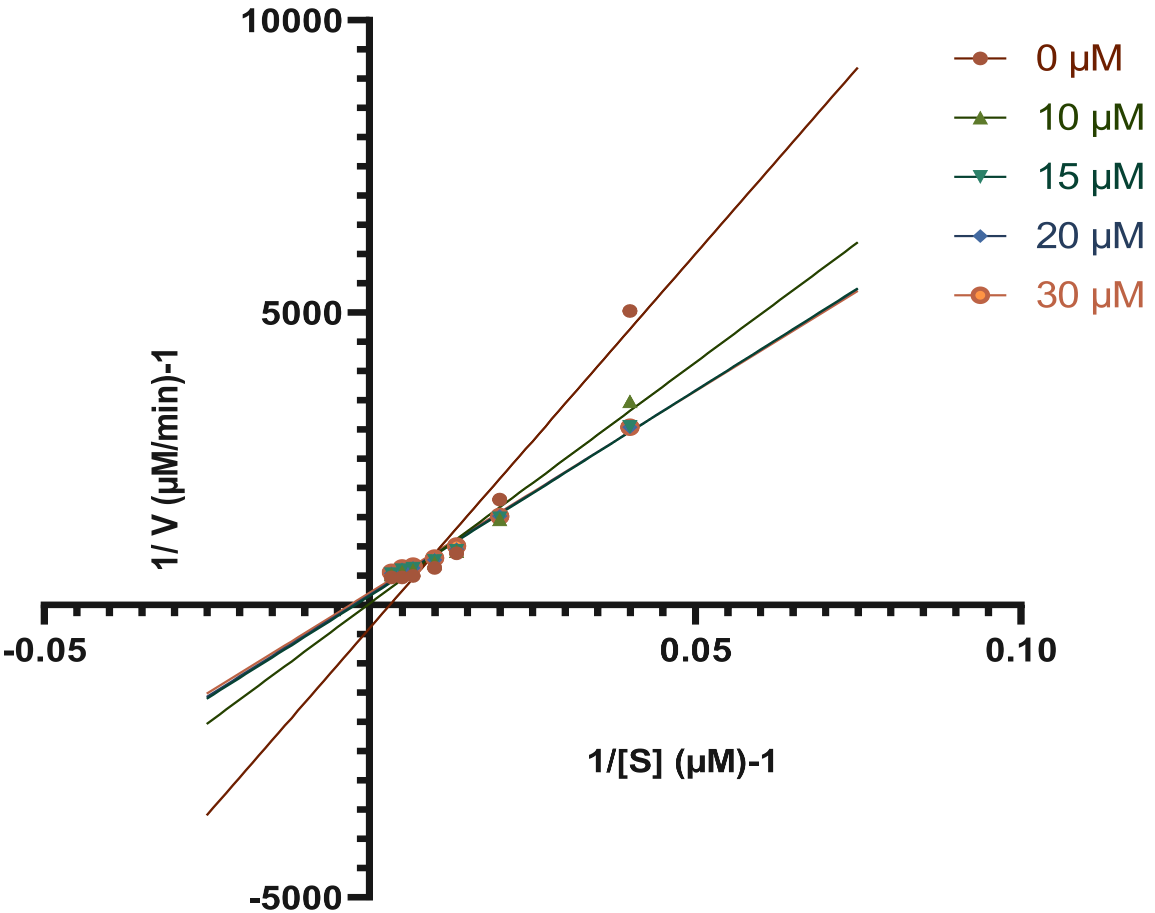

Concentration (µM)	Vmax (x 10^{-3} mM/min)	Km (µM)	Inhibition type
0	-2.5	-320.5	Mixed inhibition
10	40.6	3342.3	
15	6.6	460.8	
20	5.8	406.2	
30	4.8	333.3	

Figure S61 : Lineweaver-Burk Plot of compound 6

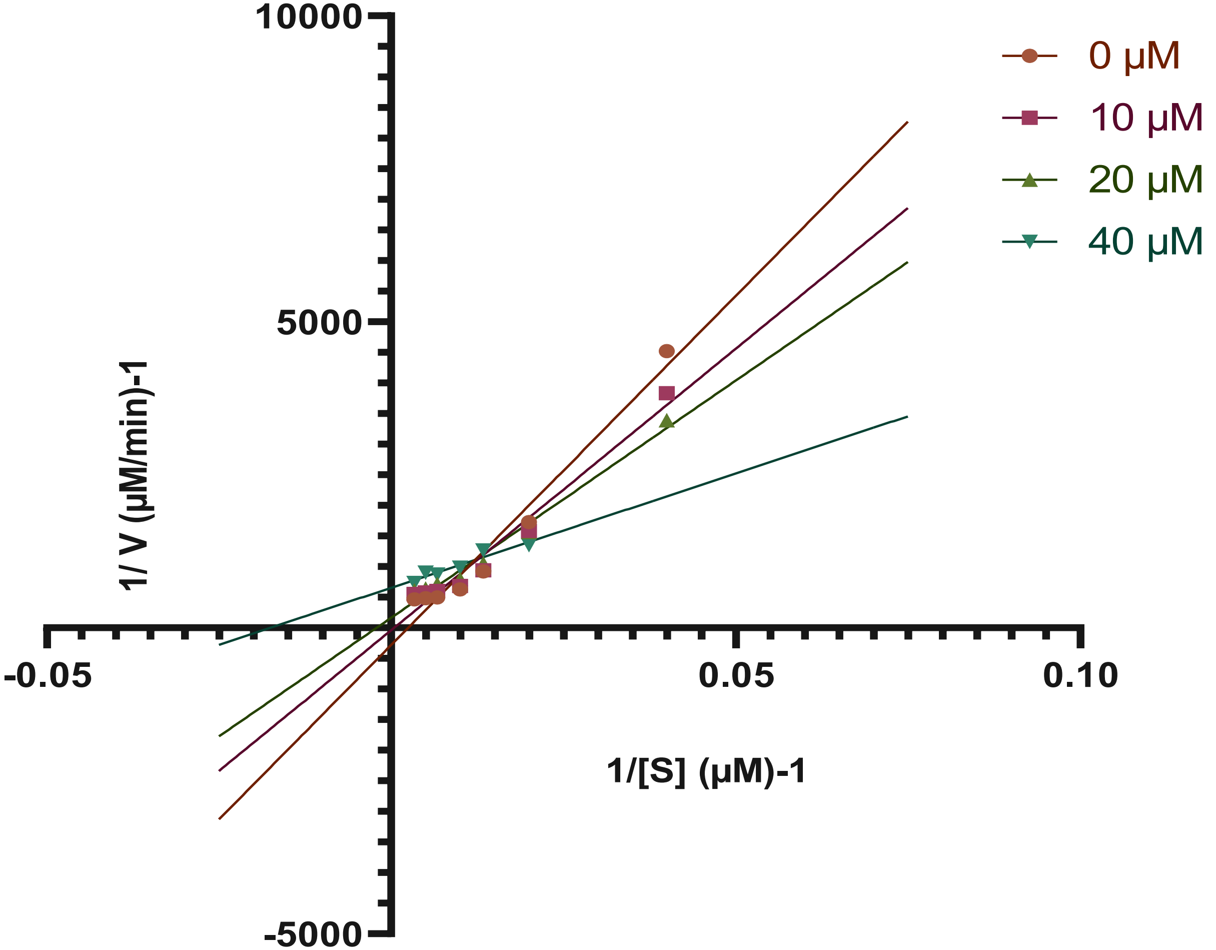

Concentration (µM)	Vmax (x 10^{-3} mM/min)	Km (µM)	Inhibition type
0	-3.6	-407.4	Mixed inhibition
10	-25.8	-2373.9	
20	6.1	471.1	
40	1.5	57.0	
